# Assessing the cardiac autonomic response to bicycle exercise in Olympic athletes with different loads of endurance training: new insights from statistical indicators based on multilevel exploratory factor analysis

**DOI:** 10.3389/fphys.2023.1245310

**Published:** 2023-10-17

**Authors:** Nadia Solaro, Massimo Pagani, Antonio Spataro, Daniela Lucini

**Affiliations:** ^1^ Department of Statistics and Quantitative Methods, University of Milano-Bicocca, Milan, Italy; ^2^ Exercise Medicine Unit, Istituto Auxologico Italiano, IRCCS, Milan, Italy; ^3^ Sports Medicine Institute CONI, Rome, Italy; ^4^ BIOMETRA Department, University of Milan, Milan, Italy

**Keywords:** aerobic training, autonomic heatmap plots, autonomic nervous system, HRV, nonparametric statistics, repeated measures, spectral analysis, cardiometabolic prevention

## Abstract

**Aim:** The mechanisms governing the organism’s response to exercise are complex and difficult to study. Spectral analysis of heart rate variability (HRV) could represent a convenient methodology for studying humans’ autonomic nervous system (ANS). However, difficulties in interpreting the multitude of correlated HRV-derived indices, mainly when computed over different time segments, may represent a barrier to its usage. This preliminary investigation addressed to elite athletes proposes a novel method describing the cardiac autonomic response to exercise based on multilevel exploratory factor analysis (MEFA), which reduces the multitude of HRV-derived indices to fewer uncorrelated ANS indicators capable of accounting for their interrelationships and overcoming the above difficulties.

**Methods:** The study involved 30 Italian Olympic athletes, divided into 15 cyclists (prevalent high-intensity endurance training) and 15 shooters (prevalent technical training with low-intensity endurance component). All athletes underwent a complete test of a dynamic protocol, constituted by a rest-stand test followed by a stepwise bicycle stress test subdivided into a single bout of progressive endurance (from aerobic to anaerobic) exercise and recovery. Then, by spectral analysis, values of 12 ANS proxies were computed at each time segment (9 epochs in all) of the complete test.

**Results:** We obtained two *global* ANS indicators (amplitude and frequency), expressing the athletes’ overall autonomic response to the complete test, and three *dynamic* ANS indicators (amplitude, signal self-similarity, and oscillatory), describing the principal dynamics over time of the variability of RR interval (RRV). Globally, cyclists have significantly higher amplitude levels (median ± MAD: cyclists 69.9 ± 20.5; shooters 37.2 ± 19.4) and lower frequency levels (median ± MAD: cyclists 37.4 ± 14.8; shooters 78.2 ± 10.2) than shooters, i.e., a parasympathetic predominance compared to shooters. Regarding the RRV dynamics, the signal self-similarity and oscillatory indicators have the strongest sensitivity in detecting the rest-stand change; the amplitude indicator is highly effective in detecting the athletes’ autonomic changes in the exercise fraction; the amplitude and oscillatory indicators present significant differences between cyclists and shooters in specific test epochs.

**Conclusion:** This MEFA application permits a more straightforward representation of the complexity characterizing ANS modulation during exercise, simplifying the interpretation of the HRV-derived indices and facilitating the possible real-life use of this non-invasive methodology.

## 1 Introduction

The complexity of mechanisms governing the organism’s response to exercise (Vatner and Pagani, 1976) has intrigued physiologists and clinicians for over a century. Considering individual bouts, it was readily apparent that the appropriate redistribution of the increased blood flow necessary for the augmented muscular activity, according to the specificity of target movements, results from the timely augmentation in cardiovascular performance (Mitchell et al., 2005) sustained by the autonomic nervous system (ANS), in particular an increase in the sympathetic drive. From about a 4:1 ratio between parasympathetic/sympathetic activity directed to the heart at rest, a progressive increase in autonomic excitatory activity would lead to a 1:4 ratio, with a marked sympathetic prevalence (White and Raven, 2014). A rapid recovery toward vagal reactivation would occur at the end of the exercise (Coote, 2010). The possibility of studying ANS during exercise in humans may help in this field of research, and (parametric and non-parametric) spectral analysis of heart rate variability (HRV) may furnish a convenient, non-invasive methodology to investigate ANS control, as the milestone observation by Akselrod et al. (1981) suggested.

Nevertheless, in the field of exercise, this methodology seems to present some pitfalls that limit its usage because of technical limitations and difficulties in interpreting the different variables derived from various types of analysis, besides considering neural coding (Cariani and Baker, 2022) and gender and age issues (Lucini et al., 2017). It is not surprising that the *reduction* of the Low-Frequency (LF) component of HRV (considered a prevalent marker of sympathetic activation to the sino-atrial node) reported by several investigators during intense aerobic exercise (Casadei et al., 1995) aroused a rich debate about the extent to which the LF spectral components (both in absolute power and normalized units) can be used as an index of sympathetic regulation (Pagani et al., 2012). Some papers (Van de Borne et al., 1997) showed that LF spectral components were blunted by heart failure, a disease characterized by sympathetic over-activity. In this context, careful attention must be given to the different aspects of tonic or phasic elements of autonomic activity, as evidenced by simultaneous recordings of nerve activity, RR variability (RRV), and systolic arterial pressure (Pagani et al., 1997). It became clear that the multiple aspects of HRV cannot be interpreted directly as a particular shift in the degree of autonomic activity and tone (Malik and Camm, 1993). Although difficult to obtain, the additional direct information provided by neural recordings with exercise seems to demonstrate a reduction of sympathetic activity with low intensity, which is subsequently overcome by intense exercise (Fisher, Young, and Fadel, 2015). In addition, other domains might play an unsuspected role: e.g., changes in respiration (Bartels et al., 2004) and venous return (Lucini et al., 2000) may play a role of hidden modulators of autonomic indices, even in the absence of exercise.

Motivated by these drawbacks and considering the potential translational value of clarifying the autonomic dynamics during the entire exercise and recovery bout (Jouven et al., 2005), we planned the present feasibility study on a small group of 30 elite athletes from the Italian Olympic team (half characterized by prevalent high-intensity endurance training—cyclists—and half by prevalent technical training with low-intensity endurance component—shooters) to investigate the autonomic dynamics during two physiological models of sympathetic activation: standing up and a stepwise bicycle stress test, which together constituted the complete test of a dynamic protocol. In the present investigation, we utilized a large RRV set of ANS proxies (i.e., HRV-derived indices representing the cardiovascular autonomic modulation, such as RR variance from tachogram and LF and HF (High-Frequency) components of RRV in absolute power and normalized units; Solaro et al., 2021a), formed by time-based and ratio-based variables (Kerkhof et al., 2019), collected over nine time segments (the epochs). Our research question regarded the possibility of assessing the global autonomic profiles and the simultaneous dynamics that govern heart period variations during a single bout of exercise in elite athletes by overcoming the limits of the spectral analysis. Accordingly, we considered the following interconnected objectives addressed in this order:

1) Disclosing and synthesizing the main autonomic domains underlying the athletes’ autonomic responses to standing up and exercise through fewer ANS statistical indicators, capable of better evidencing information that may remain hidden when considering each variable separately;
2) Studying the athletes’ autonomic responses to standing up and exercise based on the ANS indicators and comparing the extremes of the effects of training load, as represented by the two different cyclist and shooter groups, to assess potentially significant differences in the autonomic response to the complete test;
3) Deriving individual autonomic profiles from the obtained ANS indicators to describe the global and dynamic response to the complete test, from which underlying autonomic mechanisms could be inferred.

Since the collected data presented several complexities, i.e., repeated measures obtained over a small athlete set with non-normally distributed ANS proxies, we carried out the statistical analyses inherent to the three study objectives by relying on a set of integrated data-driven and non-parametric statistical methods (Moyé, 2016). We constructed the ANS indicators by applying the Multilevel Exploratory Factor Analysis (MEFA) method (Härnqvist, 1978; Muthén, 1991; Reise et al., 2005), which, besides reducing the number of the ANS proxies into a few latent factors, is capable of accounting for the repeated measures data structure by providing a variance decomposition of the athletes’ individual information into two sources of variation, i.e., between-athletes variation (*between-subjects* analysis, global representation) and within-athletes epoch variation (*within-subjects* analysis, dynamic representation). This way, the obtained ANS indicators were used to describe the athletes’ overall autonomic variation traits over the complete test regarded in its *entirety* (global representation) and the principal RRV dynamics that unfolded over the various test steps (dynamic representation). Moreover, we used non-parametric inferential methods (Hollander et al., 2014) and graphical representations to better depict ANS changes during exercise at both group and individual levels. In particular, we built so-called *autonomic heatmap plots* (also provided in the interactive form), which proved to be potent tools, especially for detecting turning points in the group and individual responses to exercise according to different sports specialties and exercise steps.

## 2 Materials and methods

### 2.1 Study population and protocol

This observational, retrospective study is part of an ongoing series of investigations focusing on using autonomic indices in elite athletes that the Independent Ethics Committee of the University of Milan approved on 23 September 2019. This protocol followed the principles of the Declaration of Helsinki and Title 45, US Code of Federal Regulations, Part 46, Protection of Human Subjects, Revised 13 November 2001, effective 13 December 2001.

In order to test a large training load history, we enrolled 30 elite athletes from the Italian Olympic team, divided into 15 cyclists and 15 shooters (Mitchell et al., 2005). The cyclist group was composed of 7 females and 8 males with a mean age of 25.67 years (±4.51 sd) and age range of 18–33 years; the shooter group of 3 females and 12 males with a mean age of 32.40 years (±5.99 sd) and age range of 23–40 years. Individual good health was ensured by the athletes’ team doctor (following Italian law that prescribes annual pre-participation screening in competing athletes) through history, blood tests, and physical examination, inclusive of an echocardiogram. All subjects had provided informed consent at the visit and agreed that their anonymized data could be used for statistical or scientific projects.

On the day of recording, after an overnight fast and a light breakfast, avoiding caffeine and intense physical activity in the preceding 24 h, subjects arrived at the clinic between 9:00 and 12:00 a.m. After the initial formalities and clinical assessment lasted about an hour, they underwent a rest-stand test followed by a maximal, incremental, stepwise bicycle stress test and an HR recovery. After electrode positioning and 5 min of horizontal rest and standing up (rest-stand test), athletes performed a symptom-limited, incremental, maximum bicycle exercise test individually titrated to reach exhaustion in about 10 min (Oggionni et al., 2021). At the CONI site (*Comitato Olimpico Nazionale Italiano*—Italian National Olympic Committee), the ECG stress test is performed with the cycle ergometer Cardioline, Cubestress XR100 (Trento, Italy). The stepwise protocol is purposely built for elite athletes by CONI as follows: Step 1: Load is set at a number of watts equal to 50% of body weight; Step 2 and beyond: every 2 min, a number of watts equal to 50% of body weight is added until load cannot be sustained any longer. Overall, the average time taken by the athletes for the exercise ramp was 10 ± 2 min. After that, subjects were asked to remain seated on the bicycle with free pedaling for about 8 min. The air conditioning system in the test room was set to 22°C ± 1°C.

### 2.2 Autonomic evaluation

The standard ECG was continuously acquired on a digital electrocardiograph (Cubestress, Cardioline, Italy) and stored on digital media for later analysis. Using dedicated software (Heartscope, AMPS, NY) (Badilini et al., 2005), first, a beat-by-beat RR interval (RRI) series (i.e., tachogram) was obtained, nominally with 1,000 samples/sec, and its quality (absence of artifacts and ectopies) was ascertained. Subsequently, the following epochs were extracted from the tachogram (average total duration 1777 ± 280 beats): 5 min nominal rest (baseline, epoch 1), followed by 5 min upright data (stand, epoch 2), and four successive exercise segments (exercise steps, epochs 3, 4, 5, and 6), a peak (epoch 7), and two final recovery phases (epochs 8 and 9), producing 9 sets of sequential RRI series. These tachogram segments were then analyzed offline with Heartscope, utilizing an autoregressive algorithm for spectral analysis with minimal operator involvement (technical details are in Solaro et al., 2021b). The program automatically computes time and frequency domain indices, selecting the best model order and verifying, in addition, the validity of the autoregressive spectral model (through Anderson’s and Akaike’s tests; Pagani et al., 1986). The frequency range of spectral components was set at >0.03 Hz for Low Frequency (LF), as usual in our laboratory, and at 0.15–0.40 for High Frequency (HF), as suggested by the Task Force of the European Society of Cardiology and the North American Society of Pacing and Electrophysiology, 1996, Table 2, for both rest and dynamic conditions. Occasional spectral components beyond this range were nominally set to 0. Recordings of subjects with recognized arrhythmias or low-frequency breathing (below 12 cycles/min) were discarded, as previously pointed out (Lucini et al., 2017). Our program applies a standard linear detrending procedure to limit the impact of nonstationarities before performing spectral analysis, as Porta et al. (2000) suggested.

The usual autonomic evaluation in our laboratory (Solaro et al., 2021b) considers a multiplicity of ANS proxies. In this work, we considered the 12 variables defined in Table 1 (several remarks about the ANS proxy selection are in Section 4.1). According to their nature, they are distinguished into the two typologies of “time-based” and “ratio-based” variables (Kerkhof et al., 2019), which broadly fit the hypothesis of two neural coding modalities (amplitude and frequency) (Pagani et al., 1997). Specifically, time-based variables have values expressed in time measurement units; ratio-based variables have values expressed in normalized numbers (in the ranges [0–1] or [0–100]) derived from their frequency in the power spectrum.

**TABLE 1:** Definition of the ANS proxies used for the study.

Time-based variables (values expressed in time measurement units)		
Variables	Units	Description
HR	beat/min	Heart rate
RR RMS	ms	Root mean square of successive RR intervals (RRIs)
RR TP	ms^2^	RR power from the autoregressive spectrum of tachogram
RR LFa	ms^2^	Absolute (a) power of LF spectral component of RRV
RR HFa	ms^2^	Absolute (a) power of HF spectral component of RRV
RMSSD	ms	Root mean square of successive RRI differences
AC	ms	Acceleration capacity, i.e., phase-rectified signal averaging (PRSA) of the RRI series with acceleration anchor point
DC	ms	Deceleration capacity, i.e., PRSA of the RRI series with deceleration anchor point

Ratio-based variables (values expressed in normalized numbers)		
Variables	Units	Description
RR LFnu	nu	Normalized unit (nu) power of LF spectral component of RRV
RR HFnu	nu	Normalized unit (nu) power of HF spectral component of RRV
RR Ro	[0–1]	Regularity index, ranging from zero (maximum complexity) to one (maximum regularity) ^(*)^
P0v	%	Pattern of no change in three-beat symbolic dynamics (in percent) ^(*)^

*Note:* This distinction of the ANS proxies into the two “time-based” and “ratio-based” typologies does not follow the classification in “time-domain” and “frequency-domain” measurements of HRV proposed by the Task Force of the European Society of Cardiology and the North American Society of Pacing and Electrophysiology (1996). It intends to differentiate the ANS proxies whose values are provided with a temporal unit of measurement (time-based) from those expressed in normalized numbers (ratio-based), thus also accounting for the complexity and non-linearity of HRV (Shaffer and Ginsberg, 2017). Note that RR LF and RR HF are thought to reflect phasic components of parasympathetic or sympathetic activity (Malik and Camm, 1993).

(*)Computational details on the regularity index RR Ro and definitions of the symbolic dynamic categories related to the number of heart period changes (among which P0v) are given in Porta et al., 2001.

### 2.3 Statistical methodology

We had to meet the three interconnected objectives listed in the Introduction by dealing with several issues of the collected data, above all, the repeated measure data structure consisting of T=9 consecutive observations of the p=12 ANS proxies listed in Table 1 collected over a small set of n=30 Olympic athletes and the non-normality of several ANS proxies. Parenthetically, the ANS proxies we used were not commensurable, i.e., not straight comparable in magnitude, variability, and unit of measurement. Moreover, being strictly correlated at both the inter-individual and intra-individual levels (due to the repeated measure structure), the ANS proxies needed to be handled in a multivariate sense rather than as single variables, aiming to reduce their number and, at the same time, preserve their informative content as best as possible.

To account for all the above and address the three objectives, we designed the statistical methodology as a set of integrated data-driven and non-parametric methods so that no *a priori* conjecture was required on the data. That represented the advanced part of the statistical data analysis in the study.

**FIGURE 1:**
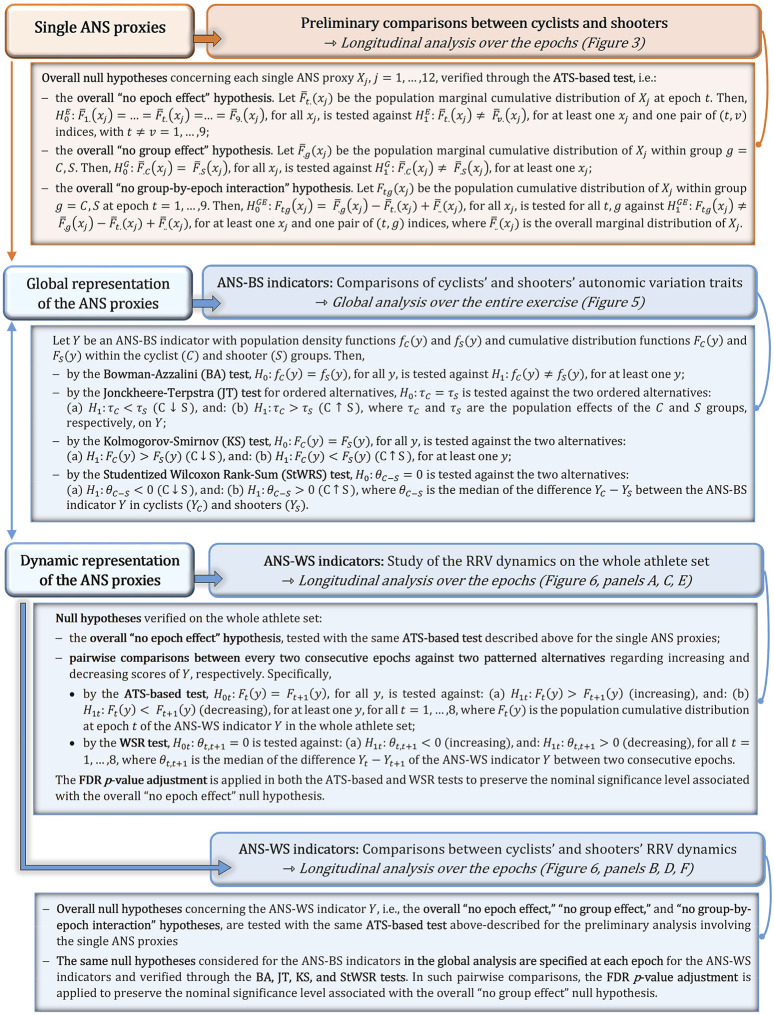
Description of the non-parametric testing procedures applied in the study.

Before that, however, we conducted a preliminary analysis of the single 12 ANS proxies to explore potentially shared trends over time and the principal differences between the cyclist and shooter groups. We focused mainly on the median profile plots of the ANS proxies depicted separately for cyclists and shooters. Such profiles were set up over the nine epochs with error bars around the medians given by the Median Absolute Deviation (MAD). Moreover, we tested the hypotheses of “no group effect,” “no epoch effect,” and “no group-by-epoch interaction” for each ANS proxy using the non-parametric ATS-based (Anova-Type Statistics) test for longitudinal data by Brunner et al. (2002). Further details are in the methodological Figure 1.

**FIGURE 2:**
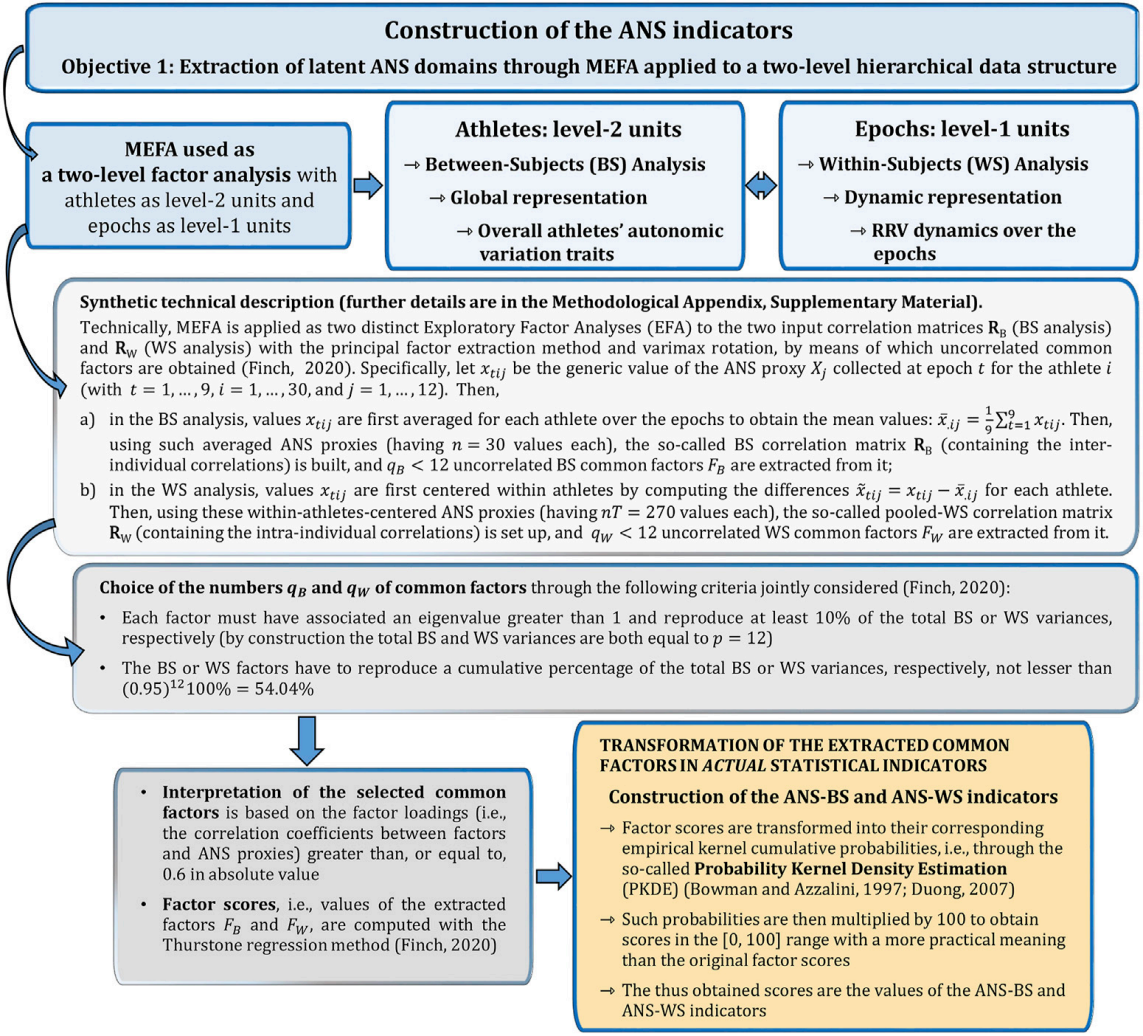
Synthetic description of the MEFA approach used to set up the ANS indicators.

Subsequently, the first step in addressing the three objectives was the application of the Multilevel Exploratory Factor Analysis (MEFA) (Härnqvist, 1978; Muthén, 1991; Reise et al., 2005), i.e., a multivariate statistical analysis method capable of detecting the main latent autonomic domains accounting for the repeated measure data structure. Given the two-level hierarchical data structure, we used MEFA in practice as a two-level factor analysis, with athletes as the level-2 units [Between-Subjects (BS) analysis] and epochs as the level-1 units [Within-Subjects (WS) analysis] (technical details on MEFA are given in the Methodological Appendix, Supplementary Material). Specifically, the advanced part of the statistical analysis was performed in the following three steps, one for each objective:

*Objective 1: Disclosing and synthesizing the main autonomic domains underlying the athletes’ autonomic responses to standing up and exercise through ANS indicators.* By MEFA, we derived a few uncorrelated common latent factors capable of disclosing the latent domains underlying the athletes’ autonomic response to the complete test. Two sets of latent factors were detected (further details are in Figure 2), each representing respectively:

a) The main latent domains describing the autonomic response to the test considered *in its entirety*, i.e., the overall autonomic variation traits characterizing the athletes (BS analysis). This MEFA part directly referred to the athletes (the level-2 units) and provided a global representation of the athletes’ autonomic response over the entire test. In practice, this analysis referred to the so-called BS correlation matrix **R**_B_ (containing the inter-individual correlations) to obtain $q_{B}<12$ uncorrelated BS common factors;
b) The main latent domains describing the RRV dynamics over the nine epochs of the complete test. That implicitly allowed the main individual autonomic profiles to be derived over time (WS analysis). This MEFA part directly referred to the epochs (the level-1 units) and provided a dynamic representation of the athletes’ autonomic response over time. In practice, this analysis referred to the so-called pooled-WS correlation matrix **R**_W_ (containing the intra-individual correlations) to obtain $q_{W}<12$ uncorrelated WS common factors.

In both BS and WS analyses, MEFA was applied to **R**_B_ and **R**_W_ as two distinct Exploratory Factor Analyses (EFA) with the principal factor extraction method and varimax rotation, through which uncorrelated common factors are derived (Reise et al., 2005; Finch, 2020). Data adequacy for the two EFA applications was initially assessed through the Kaiser-Meyer-Olkin (KMO) measure, a normalized index with values between 0 and 1. KMO values below 0.6 typically indicate poor or unacceptable factorial solutions, thus requiring some action (e.g., removing/substituting one or more variables from the initial set) (Dziuban and Shirkey, 1974). Besides this, Figure 2 summarizes the criteria employed to choose the numbers $q_{B}$ and $q_{W}$ of common latent factors to keep in analysis and the way such factors were interpreted and then expressed in *actual* statistical indicators with values in the [0, 100] range. Throughout the study, we labeled these indicators as ANS-BS or ANS-WS, depending on the analysis type. The values of the ANS indicators were called “scores.”

We assessed the accuracy of the main findings concerning the ANS indicators through the following steps. First, we checked for potential sex and age effects on the ANS indicators by fitting a series of quantile regression models (Koenker, 2005), each having an ANS indicator in turn as the dependent variable and sex (included as a dummy variable, with 0 = female and 1 = male), age, and their interaction as independent variables. Then, we tested the hypotheses of the absence of sex, age, and their interaction effects. As for the ANS-WS indicators, this procedure was applied at each epoch. Second, we applied a specific resampling technique, i.e., the non-parametric stratified balanced bootstrap (Davison et al., 1986; Davison and Hinkley, 1997). In practice, 1,000 bootstrap samples (with repetition) were generated for both the BS and WS analyses such that cyclists and shooters entered each sample with the same proportion, and the bootstrap distributions of the ANS indicators were obtained. The uncertainty extent of the main results (e.g., factor loadings) was then assessed through non-parametric 95% bootstrap confidence intervals (C.I.s) computed with the BC_a_ method and the option “infinitesimal jackknife” (DiCiccio and Efron, 1996).

*Objective 2: Studying the autonomic response to standing up and exercise in the whole athlete set and comparing cyclists and shooters through the ANS indicators.* As a first analysis step, the ANS-BS and ANS-WS indicator distributions were dealt with by estimating their within-group density curves with the Bowman-Azzalini (BA) method (Bowman and Azzalini, 1997). In the specific case of the ANS-WS indicators, this estimation was carried out at each epoch. At the same time, to better display the disclosed RRV dynamics over time, the median profile plots of the ANS-WS indicators (with error bars around the medians given by 95% bootstrap C.I.s) were built for the whole athlete set and the cyclist and shooter groups.

Figure 1 resumes the non-parametric testing procedures applied to both the global ANS-BS indicators and the dynamic ANS-WS indicators. Regarding the inspection of potential differences between cyclists and shooters based on the estimated within-group density curves, we performed two complementary analyses, by which we compared:

a) The cyclists’ and shooters’ ANS-BS indicator distributions to detect significant overall differences in their autonomic traits;
b) The cyclists’ and shooters’ ANS-WS indicator distributions to disclose significant differences in their RRV dynamics at each epoch.

In particular, to test the hypothesis of “no group difference” (or also, “no group effect”), we relied on the BA permutation test (Bowman and Azzalini, 1997), the Jonckheere-Terpstra (JT) permutation test for ordered alternatives, the Kolmogorov-Smirnov (KS) bootstrap test, and the Studentized Wilcoxon Rank-Sum (StWRS) permutation test (Hollander et al., 2014; Helwig, 2019). In the case of the ANS-WS indicators, the False Discovery Rate (FDR) *p*-value adjustment (Benjamini and Hochberg, 1995) was applied to preserve the nominal significance level associated with the overall “no group effect” null hypothesis tested over all the epochs together.

Besides this, we studied the trends of the RRV dynamics over time based on the median profile plots of the ANS-WS indicators. We tested the overall hypothesis of “no epoch effect” in the whole athlete set using the non-parametric ATS-based test (Brunner et al., 2002). Then, we studied the presence of increasing or decreasing trends between every two consecutive epochs by applying the ATS-based test and the permutation Wilcoxon signed-rank (WSR) test (Helwig, 2019) with the FDR *p*-value adjustment (Benjamini and Hochberg, 1995). At the same time, we compared the cyclists’ and shooters’ RRV dynamic trends by testing the hypotheses of “no group effect,” “no epoch effect,” and “no group-by-epoch interaction” for each ANS-WS indicator using the non-parametric ATS-based test (Brunner et al., 2002).

*Objective 3: Deriving individual autonomic profile.* We set up the so-called *autonomic heatmap plots* to describe each athlete’s autonomic profile as his/her response to the complete test considered in its entirety and at every epoch. Such profiles represent the most detailed description because they combine the BS and WS analyses. The athletes’ scores on a specific ANS-BS indicator were paired with those on the related-meaning ANS-WS indicator and depicted in the same heatmap plot. Graphic cells were then colored by gradually increasing their tonality according to the score magnitude, i.e., lighter colors for lower scores and darker colors for higher scores. Numeric data underneath the graphic cells were reported unencoded in the interactive heatmap plots, where the individual scores can be displayed by mouse hovering. This way, one can easily visualize, also for comparisons, each athlete’s general autonomic state over the exercise entirety (BS analysis) and each athlete’s autonomic response to exercise along the epochs (WS analysis).

Throughout the study, the nominal test significance level was set at 0.05. We performed the statistical analyses with the R software, version 4.3.0 (R Core Team, 2023), together with the following contributed packages: “corrplot” for the correlation plots of the correlation matrices **R**_B_ and **R**_W_ (Wei and Simko, 2021); “psych” (Revelle, 2022) for the implementation of the two-level factor analysis; “ks” (Duong, 2007; Duong, 2022) for PKDE; “quantreg” (Koenker, 2023) for the quantile regression models; “boot” (Canty and Ripley, 2021) for the bootstrap; “nparLD” (Noguchi et al., 2012) for the ATS-based test; “nptest” (Helwig, 2021) for the permutation version of the WSR and StWRS tests; “sm” (Bowman and Azzalini, 2021) for the BA test and smoothed empirical density curves; “DescTools” (Signorell et al., 2021) for the permutation JT test; “ggplot2” (Wickham, 2016) for the construction of all the other graphs; “plotly” (Sievert, 2020) for the interactive autonomic heatmap plots.

## 3 Results

**FIGURE 3:**
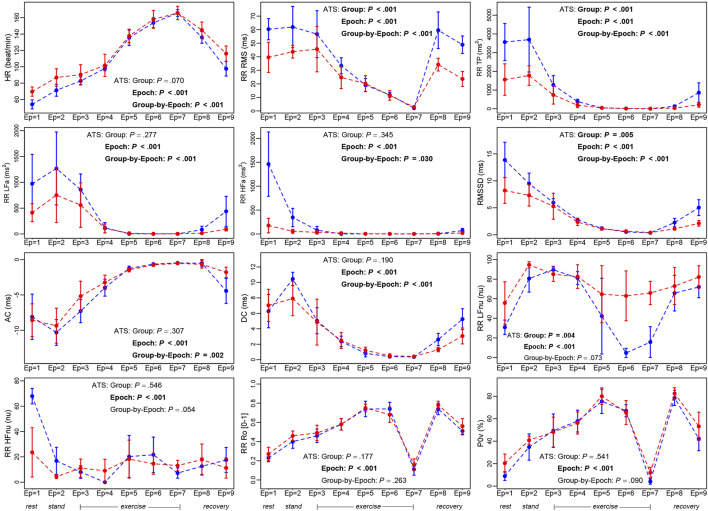
Within-group median profile plots of the study ANS proxies set up for the cyclist and shooter groups over the nine epochs. *Legend:* In each panel, blue profiles regard the cyclist group, and red profiles the shooter group. Error bars around the median are given by ± MAD (Median Absolute Deviation). Numerical data are reported in Supplementary Table S1, with the median and MAD computed over all the epochs, and in Supplementary Tables S3, S4, with the median and MAD computed at each epoch. The median profile plots built over the whole athlete set are displayed in Supplementary Figure S1, with numerical data in Supplementary Table S2. Supplementary Table S5 reports the test results of several pairwise comparisons of interest (i.e., stand-rest, peak-rest, and recovery last phase-peak) considered in both the whole athlete set and the cyclist and shooter groups. Meaning of the ATS-based test (see description in Figure 1):− “Group” intends the ATS-based test for the null hypothesis of “no group effect,”− “Epoch” intends the ATS-based test for the null hypothesis of “no epoch effect,”− “Group-by-Epoch” intends the ATS-based test for the null hypothesis of “no interaction between groups and epochs,” evaluated for each ANS proxy. Significant results at the 0.05 level are written in bold.

Descriptive statistics of the 12 ANS proxies and several non-parametric test results (Supplementary Tables S1–S5) indicate the presence of trend patterns that are worth examining further. On this point, Figure 3 displays the within-group median profile plots for each ANS proxy referred to the cyclist and shooter groups, along with the ATS-based test results. Two remarks are worth making. Firstly, regardless of the group, some trend patterns shared by specific ANS proxies are visible over the epochs (also see Supplementary Figure S1). Several median profiles have a similar increasing (e.g., RR Ro and P0v) or decreasing trend (e.g., RR RMS, RMSSD, and DC) until peak exercise (epoch 7) with reversal afterward. Moreover, RR TP, RR LFa, and RR HFa median values come close to zero during the exercise up to epoch 7, then slightly increase during the recovery phases; AC and DC have a similar trend but opposite directions. Besides this, in addition to the well-known excitatory effects of standing up, RR LFnu increases until epochs 3 and 4; then, it dramatically decreases till the exercise peak (epoch 7) and returns to previous higher levels during recovery (Supplementary Figure S1). A similar inverse profile is present for RR HFnu. In order then to better understand the athletes’ hemodynamic changes during exercise at the various epochs, we referred to the guidelines by Pelliccia et al., 2021, Table 4, and calculated the percentages of the maximal heart rate (reached at epoch 7) for each athlete (Supplementary Figure S6) along with descriptive statistics (Table 2).

**TABLE 2:** Descriptive statistics (mean ± sd and median ± MAD) of the cyclists’ and shooters’ percentages of maximal heart rate (HR) recorded at rest and stand and during the bicycle stress test.

Cyclists									
	HR.1%	HR.2%	HR.3%	HR.4%	HR.5%	HR.6%	HR.7%	HR.8%	HR.9%
Mean	*32.34*	43.49	50.58	60.49	81.09	**93.93**	100	80.43	56.65
sd	4.19	7.53	4.35	4.64	4.25	2.18	0	5.92	7.06
Median	*32.61*	42.51	50.11	59.44	81.56	**93.05**	100	81.33	57.65
MAD	1.42	4.25	3.18	2.06	3.39	1.42	0	2.16	4.23

Shooters									
	HR.1%	HR.2%	HR.3%	HR.4%	HR.5%	HR.6%	HR.7%	HR.8%	HR.9%
Mean	*42.20*	52.63	54.73	63.46	82.31	**94.58**	100	86.18	69.57
sd	6.96	9.24	8.05	7.86	4.53	2.19	0	3.16	4.81
Median	*39.56*	52.74	55.33	65.38	82.01	**94.61**	100	85.93	69.66
MAD	3.18	7.46	5.38	5.03	3.15	1.07	0	1.79	3.69

low intensity HR%< 55	moderate intensity 55 ≤ HR% < 75	high intensity 75 ≤ HR% ≤ 90	very high intensity HR% > 90

*Legend*: Exercise intensity levels based on the guidelines in Pelliccia et al. (2021, Table 4):

*Note:* Individual percentages of maximal heart rate at each epoch (Supplementary Table S6) are given by $\text{HR}.t\%=\left(\text{HR}.t/{\text{HR}}_{\max}\right)100\%$ , where HR.t is the heart rate at epoch *t* ( t=1,…,9 ) and ${\text{HR}}_{\max}$ is the individual maximal heart rate recorded during the bicycle stress test. For each group, 100% corresponds to the mean or median ${\text{HR}}_{\max}$ . The minimum mean and median HR.t% over the epochs are written in italics; the highest mean and median HR.t% smaller than 100% are written in bold.

As a second remark, the ATS-based test supports the presence of a significant epoch effect on all the studied ANS proxies, while in almost all the cases, there is a significant group-by-epoch interaction, thus suggesting that, overall, cyclists and shooters have different autonomic responses to the complete test (Figure 3).

**FIGURE 4:**
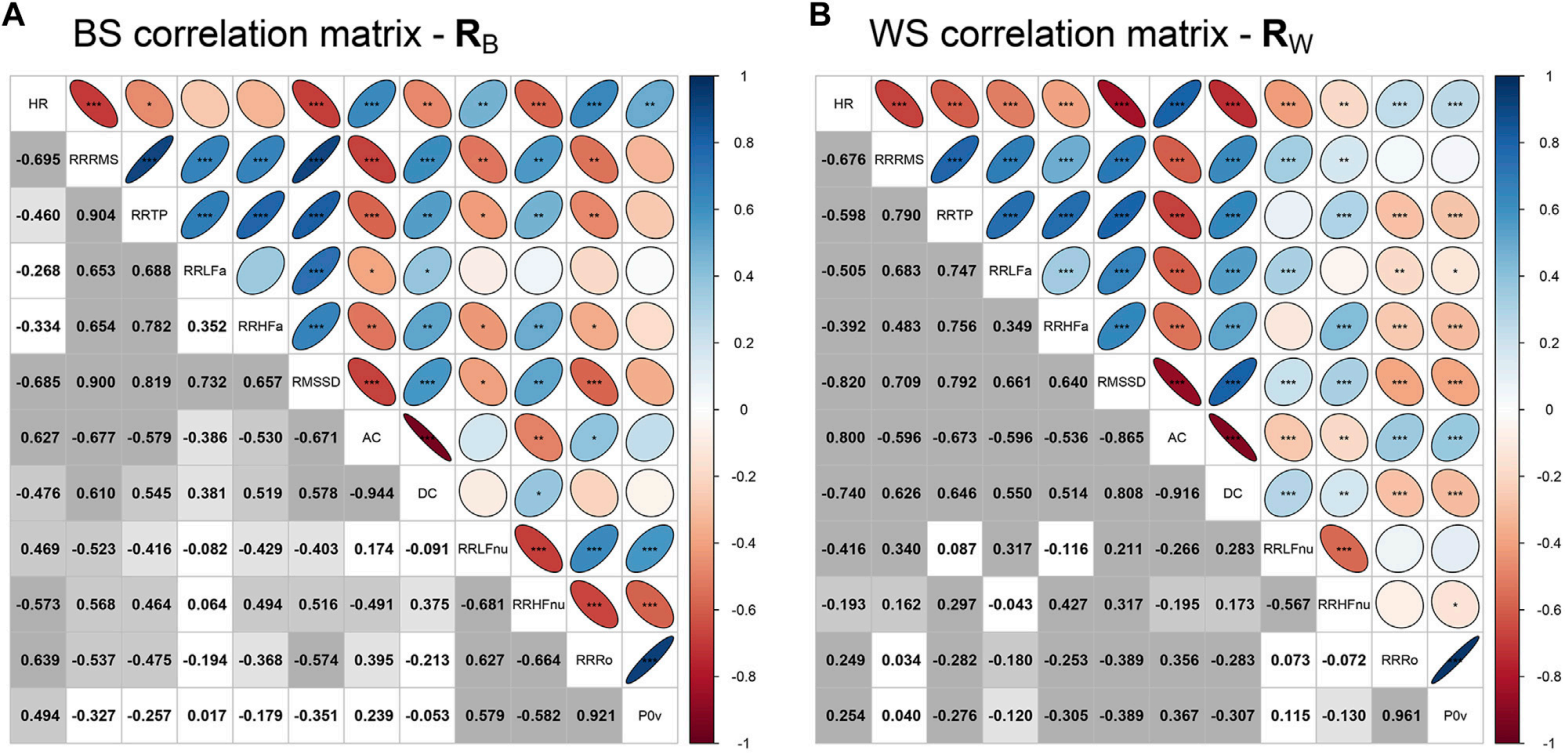
Correlation plots of the between-subjects correlation matrix **R**_B_ (containing the inter-individual correlation coefficients) and the within-subjects correlation matrix **R**_W_ (containing the intra-individual correlation coefficients). *Legend.* Panel A: In the lower triangular part of the correlation plot of **R**_B_, displayed values are the Pearson correlation coefficients computed for every pair of the averaged ANS proxies $\left({\bar{X}}_{j},{\bar{X}}_{l}\right)$ , for all j,l=1,…,12 with j≠l , (Methodological Appendix in Supplementary Material). Panel B: In the lower triangular part of the correlation plot of **R**_W_, displayed values are the Pearson correlation coefficients computed for every pair of the within-athletes-centered ANS proxies $\left({\tilde{X}}_{j},{\tilde{X}}_{l}\right)$ , for all j,l=1,…,12 with j≠l , (Methodological Appendix in Supplementary Material). In each numerical cell, background gray shades denote the empirical significance level of every performed test for null correlation: 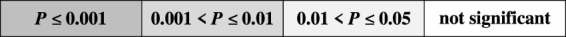
Specifically, the tested null hypotheses are: in Panel A, $H_{0}:\rho \left({\bar{X}}_{j},{\bar{X}}_{l}\right)=0$ vs. $H_{1}:\rho \left({\bar{X}}_{j},{\bar{X}}_{l}\right)\neq 0$ , for all j≠l ; in Panel B, $H_{0}:\rho \left({\tilde{X}}_{j},{\tilde{X}}_{l}\right)=0$ vs. $H_{1}:\rho \left({\tilde{X}}_{j},{\tilde{X}}_{l}\right)\neq 0$ , for all j≠l . In the upper triangular part of the two correlation plots, the correlation coefficients are represented as ellipses, including the empirical significance level code internally: *** = *P* ≤ .001. ** = .001 < *P* ≤ .01. * = .01 < *P* ≤ .05. KMO factor adequacy measure: KMO in BS analysis = 0.704 [95% bootstrap C.I.: [0.686, 0.762]; KMO in WS analysis = 0.778 [95% bootstrap C.I.: [0.739, 0.816].

The above-noted similar trends shared by specific ANS proxies are indicative of the presence of intense correlations among them. On this point, Figure 4 displays the correlation plots of the BS correlation matrix **R**_B_ (with the inter-individual correlations) and the WS correlation matrix **R**_W_ (with the intra-individual correlations) together with the significance test results for the hypotheses of null correlations. Many significant and high (negative or positive) correlations are apparent in both matrices, e.g., the high positive BS and WS correlation coefficients of RR TP and RR RMS or the high negative BS and WS correlation coefficients of AC and RR RMS. Moreover, concerning the adequacy of both **R**_B_ and **R**_W_ for factor analysis, the KMO measure equals 0.704 in BS analysis and 0.778 in WS analysis, thus denoting that the two correlation matrices are sufficiently suitable for MEFA application.

All the above considerations fully justify the MEFA application to synthesize the 12 ANS proxies in fewer indicators. The following describes the construction of such ANS indicators and their main findings.

*Objective 1: Construction of the ANS indicators*. Regarding the BS analysis, Table 3 reports the factor loadings of the ANS proxies and the first $q_{B}=2$ common factors extracted from **R**_B_ (Figure 4, panel A) and kept in the study according to the criteria listed in Figure 2. The two factors together explain 68.01% of the total BS variance (95% C.I.: [62.35%, 79.13%]). BS factor 1 (38.89% of total BS variance; 95% C.I.: [34.16%, 49.51%]) represents the Amplitude domain because it is highly positively correlated with almost all the time-based variables, except AC, for which the correlation is highly negative. At the same time, having a loading not exceeding the fixed threshold, HR has a negligible link with this factor. BS factor 2 (29.12% of total BS variance; 95% C.I.: [20.59%, 36.89%]) denotes the Frequency domain since it is strongly correlated with the ratio-based variables (positively with RR LFnu, RR Ro, and P0v, and negatively with RR HFnu). After extraction, the two BS factors are re-expressed by PKDE into the amplitude and frequency BS indicators (labeled in the following with AMP-BS-Ind and FRE-BS-Ind, respectively) with scores in the [0, 100] interval.

**TABLE 3:** Between-subjects (BS) factor analysis with the principal factor extraction method arrested to the first two common factors: Rotated factor loadings with the varimax method and 95% bootstrap confidence intervals (C.I.s.).

Variables	BS factor 1			BS factor 2		
	Loadings	95% bootstrap C.I.s(*)		Loadings	95% bootstrap C.I.s(*)	
RR RMS	0.867▲	0.723	0.954	−0.405	−0.817	−0.241
RR TP	0.840▲	0.430	0.947	−0.290	−0.915	−0.167
RR LFa	0.681▲	0.329	0.892	0.031	−0.235	0.569
RR HFa	0.654▲	0.324	0.797	−0.275	−0.789	−0.145
RMSSD	0.854▲	0.721	0.987	−0.380	−0.868	−0.108
AC	−0.760▲	−0.937	−0.294	0.231	0.019	0.975
DC	0.752▲	0.102	0.926	−0.068	−0.979	0.106
HR	−0.494	−0.805	−0.266	0.559	0.229	0.853
RR LFnu	−0.185	−0.833	0.014	0.707▲	0.386	0.881
RR HFnu	0.340	0.158	0.844	−0.708▲	−0.878	−0.323
RR Ro	−0.229	−0.867	−0.097	0.915▲	0.801	0.976
P0v	0.014	−0.142	0.471	0.904▲	0.739	0.997
% of total BS variance	38.89%	34.16%	49.51%	29.12%	20.59%	36.89%
cumulative % of total BS variance	38.89%	34.16%	49.51%	68.01%	62.35%	79.13%
% of total BS communality	57.18%	50.98%	71.42%	42.82%	31.63%	49.67%
cumulative % of total BS communality	57.18%	50.98%	71.42%	100.00%	---	---

*Note:* Total BS communality (i.e., total reproduced BS variance) = 8.161, total BS variance = 12, percentage of total BS variance explained = 68.01%, (95% bootstrap C.I.: [62.35%, 79.13%]). Printed values are the factor loadings, i.e., correlation coefficients between the ANS proxies and the first $q_{B}=2$ BS common factors. Black triangles mark the loadings greater than, or equal to, 0.6 in absolute value. Interpretation underlying the first two BS factors: BS Factor 1 = Amplitude BS factor (variables and loadings colored in light blue), BS Factor 2 = Frequency BS factor (variables and loadings in yellow).

(*)Due to the small number of observations (*n* = 30 values for each averaged ANS, proxy), numerical problems were encountered during the bootstrap procedure applied to the BS factor analysis. So-called “ultra Heywood” cases occurred in which one or more communalities were computed greater than 1, so the corresponding solutions (123 in all) were discarded from the study. The bootstrap analyses are then based on 877 out of 1,000 bootstrap samples.

Complementary to the BS analysis, the WS analysis provides the principal descriptors of the RRV dynamics unfolded during the complete test. Table 4 displays the factor loadings of the first $q_{W}=3$ common factors extracted from **R**_W_ (Figure 4, panel B), which together reproduce 75.83% of the total WS variance (95% C.I.: [73.47%, 80.14%]). Once again, the first factor, WS factor 1 (45.62% of total WS variance; 95% C.I.: [43.09%, 48.93%]), represents the Amplitude domain because it strongly correlates with the same time-based variables linked to the BS factor 1, in addition to HR (high negative correlation). Regarding the ratio-based variables, WS factor 2 (17.66% of total WS variance; 95% C.I.: [17.39%, 18.64%]) represents the Signal Self-Similarity domain, given its high positive correlations with RR Ro and P0v, while WS factor 3 [12.55% of total WS variance; 95% C.I.: (11.45%, 14.61%)] denotes the Oscillatory domain since it is highly positively correlated with RR LFnu and negatively with RR HFnu. As before, the amplitude, signal self-similarity, and oscillatory WS factors are transformed by PKDE into the corresponding indicators with scores in the [0, 100] interval. These three ANS-WS indicators are labeled in the following: AMP-WS-Ind (amplitude), SSS-WS-Ind (signal self-similarity), and OSC-WS-Ind (oscillatory), respectively.

**TABLE 4:** Within-subjects (WS) factor analysis with the principal factor extraction method arrested to the first three common factors: Rotated factor loadings with the varimax method and 95% bootstrap confidence intervals (C.I.s.).

Variables	WS factor 1			WS factor 2			WS factor 3		
	Loadings	95% bootstrap C.I.s(*)		Loadings	95% bootstrap C.I.s(*)		Loadings	95% bootstrap C.I.s(*)	
HR	−0.810•	−0.847	−0.772	0.149	0.103	0.312	−0.105	−0.721	−0.022
RR RMS	0.857•	0.814	0.903	0.189	0.134	0.267	0.005	−0.065	0.469
RR TP	0.854•	0.823	0.887	−0.107	−0.196	−0.008	−0.248	−0.412	−0.144
RR LFa	0.723•	0.661	0.798	−0.034	−0.139	0.029	0.131	0.001	0.267
RR HFa	0.603•	0.517	0.716	−0.156	−0.237	−0.109	−0.442	−0.527	−0.368
RMSSD	0.914•	0.859	0.949	−0.257	−0.355	−0.221	−0.123	−0.213	−0.059
AC	−0.856•	−0.903	−0.779	0.271	0.215	0.434	−0.011	−0.243	0.073
DC	0.829•	0.771	0.894	−0.203	−0.442	−0.151	0.022	−0.087	0.287
RR Ro	−0.153	−0.205	−0.121	0.953•	0.899	0.968	0.041	−0.012	0.138
P0v	−0.150	−0.203	−0.108	0.962•	0.948	0.973	0.110	0.077	0.322
RR LFnu	0.355	0.276	0.496	0.091	0.034	0.189	0.782•	0.702	0.868
RR HFnu	0.199	0.131	0.513	−0.027	−0.116	0.021	−0.761•	−0.843	−0.624
% of total WS variance	45.62%	43.09%	48.93%	17.66%	17.39%	18.64%	12.55%	11.45%	14.61%
cumulative % of total WS variance	45.62%	43.09%	48.93%	63.28%	61.14%	66.85%	75.83%	73.47%	80.14%
% of total WS communality	60.16%	58.03%	62.16%	23.29%	22.17%	25.32%	16.55%	15.27%	18.71%
cumulative % of total WS communality	60.16%	58.03%	62.16%	83.45%	81.79%	85.26%	100.00%	---	---

*Note:* Total WS communality (i.e., total reproduced WS variance) = 9.099, total WS variance = 12, percentage of total WS variance explained = 75.83%, (95% bootstrap C.I.: [73.47%, 80.14%]). Printed values are the factor loadings, i.e., correlation coefficients between the ANS proxies and the first $q_{W}=3$ WS common factors. Black circles mark loadings greater than, or equal to, 0.6 in absolute value. Interpretation underlying the first three WS factors: WS Factor 1 = Amplitude WS factor (variables and loadings colored in light blue), WS Factor 2 = Signal Self-Similarity WS factor (variables and loadings in yellow), WS Factor 3 = Oscillatory WS factor (variables and loadings in green).

(*)Bootstrap analyses are based on 1,000 samples (i.e., no numerical problem occurred during the bootstrap procedure).

After construction, these ANS indicators are checked for potential sex and age bias. The analysis based on the quantile regression models does not indicate the presence of significant sex, age, and sex-by-age effects on any indicator (Supplementary Tables S7, S8). Accordingly, the ANS indicators have not been adjusted for sex and age effects before proceeding to the subsequent analyses [by applying, e.g., the statistical methodology in Solaro et al. (2021a)].

**FIGURE 5:**
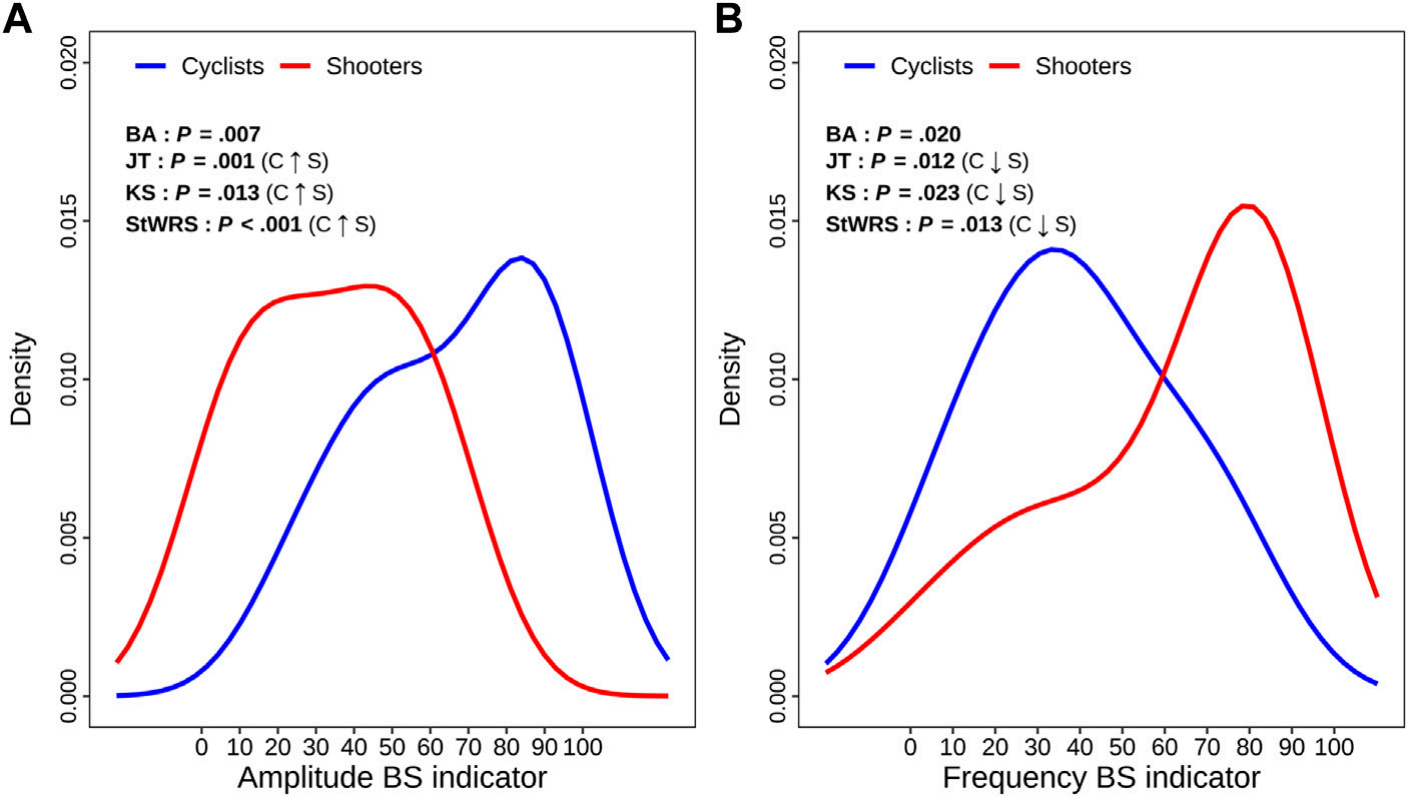
Panel plot of the estimated density curves of the amplitude and frequency BS indicator distributions in comparing the cyclist and shooter groups. *Legend:* The meaning of the ANS-BS indicator scores is reported below in Supplementary Figure S2, which displays the total and within-group beeswarm plots (along with box plots on the background) of the amplitude and frequency BS indicator distributions. The meaning of the statistical tests reported in each panel is given in Figure 1. Significant results at the 0.05 level are written in bold.

*Objective 2: Statistical analyses based on the ANS indicators.* Figure 5 displays the two within-group density curves estimated for AMP-BS-Ind (panel A) and FRE-BS-Ind (panel B), along with the non-parametric test results (Figure 1). This analysis compares cyclists and shooters based on their principal overall autonomic variation traits captured over the entire test according to a global representation. The BA test confirms that the cyclists’ and shooters’ curves differ significantly on both indicators. Moreover, the JT, KS, and StWRS tests indicate that the cyclists’ AMP-BS-Ind distribution (blue curve) is significantly more concentrated on higher scores than shooters (red curve), while the cyclists’ FRE-BS-Ind distribution is significantly more concentrated on lower scores than shooters.

**FIGURE 6:**
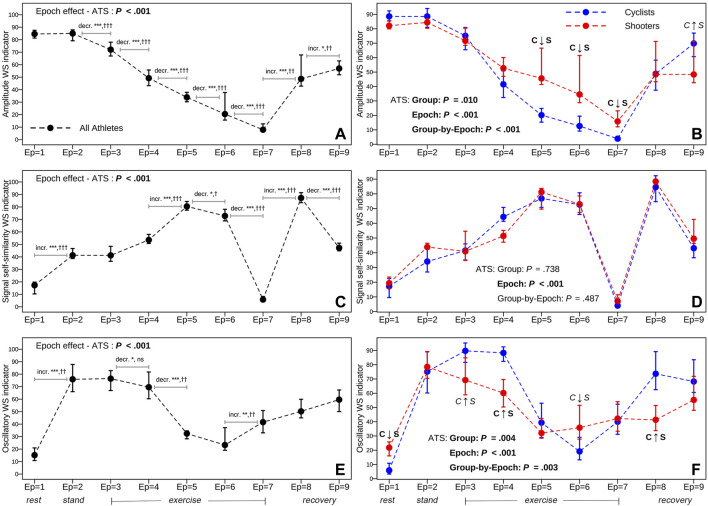
Total (first column of panels) and within-group (second column) median profile plots, plus 95% bootstrap confidence intervals, of the amplitude, signal self-similarity, and oscillatory WS indicator scores over the nine epochs. *Note:* Numerical data concerning the median scores and 95% bootstrap C.I.s are reported in Supplementary Table S9 for the whole athlete set and Supplementary Table S10 for the cyclist and shooter groups. The meaning of the ANS-WS indicator scores is reported below in Supplementary Figure S3, which displays the total and within-group beeswarm plots (along with box plots on the background) of the three ANS-WS indicator distributions. The ANS-WS indicators are ordered decreasingly according to their percentage of reproduced total WS variance (Table 4). The meaning of the statistical tests reported in each panel is given in Figure 1. Significant results at the 0.05 level regarding the overall null hypotheses: “no epoch effect,” “no group effect,” and “no group-by-epoch interaction,” tested with the ATS-based test, are written in bold. Significance level code for the comparisons between two consecutive epochs in the first column of panels:−ATS-based test: *significant at 0.05 level, **significant at 0.01 level, ***significant at 0.001 level (numerical data are in Supplementary Table S11); Wilcoxon signed-rank (WSR) test: ^†^significant at 0.05 level, ^††^significant at 0.01 level, ^†††^significant at 0.001 level; ns: not significant (numerical data are in Supplementary Table S12). Meaning of the labels “C ↑ S” and “C ↓ S” in the second column of panels (numerical data are in Supplementary Tables S13–S16; Supplementary Figures S4–S6): C ↑ S and C ↓ S in bold indicate that all the BA, JT, KS, and StWRS tests, together with 95% bootstrap C.I.s, agree in indicating that cyclists generally have higher (C ↑ S) or lower scores (C ↓ S) than shooters; the same labels in italics indicate that not all the tests produce significant results. The absence of the labels indicates that at least two significant results have not been found.

The two columns of panels in Figure 6 display two types of analyses concerning the three RRV dynamics over time expressed by the respective ANS-WS indicators (dynamic representation). These analyses are based on the median profile plots built on the whole athlete set (first column of panels) and the within-group median profile plots for the cyclist and shooter groups (second column). Regarding the whole athlete set, the ATS-based test proves the presence of a significant epoch effect in all the cases. However, it can be immediately noted that the three RRV dynamics have very different trends over the epochs. AMP-WS-Ind (panel A) has a U-like median trend, starting at similar highest levels in rest and stand (epochs 1–2), then significantly decreasing during the exercise steps until the peak (epochs 3–7) and subsequently significantly increasing during the recovery steps (epochs 8–9). On the other hand, SSS-WS-Ind (panel C) has a wave-like median trend, increasing from rest to stand until the third exercise step (epochs 1–5), then decreasing until the peak (epoch 7), lastly increasing again in the first recovery step (epoch 8) and subsequently decreasing in the last recovery step (epoch 9). Finally, OSC-WS-Ind (panel E) has an inverted U-like median trend in the first six epochs, in particular with a significant increase from rest to stand (epochs 1–2), and then a monotonic increase from epoch 6 until the last recovery step (epoch 9).

Regarding the comparisons between cyclists and shooters (Figure 6, second column of panels), the ATS-based test signals a significant group-by-epoch interaction on AMP-WS-Ind and OSC-WS-Ind (the group and epoch main effects are also significant), i.e., there is evidence that cyclists and shooters differ significantly on the amplitude and oscillatory dynamics during the test. In contrast, no significant group or group-by-epoch effects are present on SSS-WS-Ind; the only significant effect concerns the epochs. A more in-depth investigation is performed at each epoch by examining the non-overlapping bootstrap C.I.s along with the two within-group estimated density curves (Supplementary Figures S4–S6) with the tests mentioned in Section 2.3 and Figure 1. Significant results of all these procedures are resumed in the second-column panels through the labels “C ↑ S” (cyclists with higher scores) and “C ↓ S” (cyclists with lower scores). In the AMP-WS-Ind case (panel B), the two groups differ mainly in the high-intensity exercise steps (epochs 5–7; see Table 2), where the shooters’ density curve significantly concentrates on higher scores than cyclists. In contrast, at epoch 9, the JT and StWRS tests indicate that the cyclists’ density curve significantly concentrates on higher scores than shooters (Supplementary Figure S4). The same analysis carried out for SSS-WS-Ind (panel D and Supplementary Figure S5) confirms the ATS-based test results: No significant difference between the two groups is found. Moreover, in the OSC-WS-Ind case (panel F and Supplementary Figure S6), all the considered tests agree in indicating that the shooters’ density curve significantly concentrates on higher scores than cyclists at epoch 1 (at epoch 6, only the KS test is significant), and on lower scores at epochs 3 (with the only exception of the bootstrap C.I.s), 4, and 8.

**FIGURE 7:**
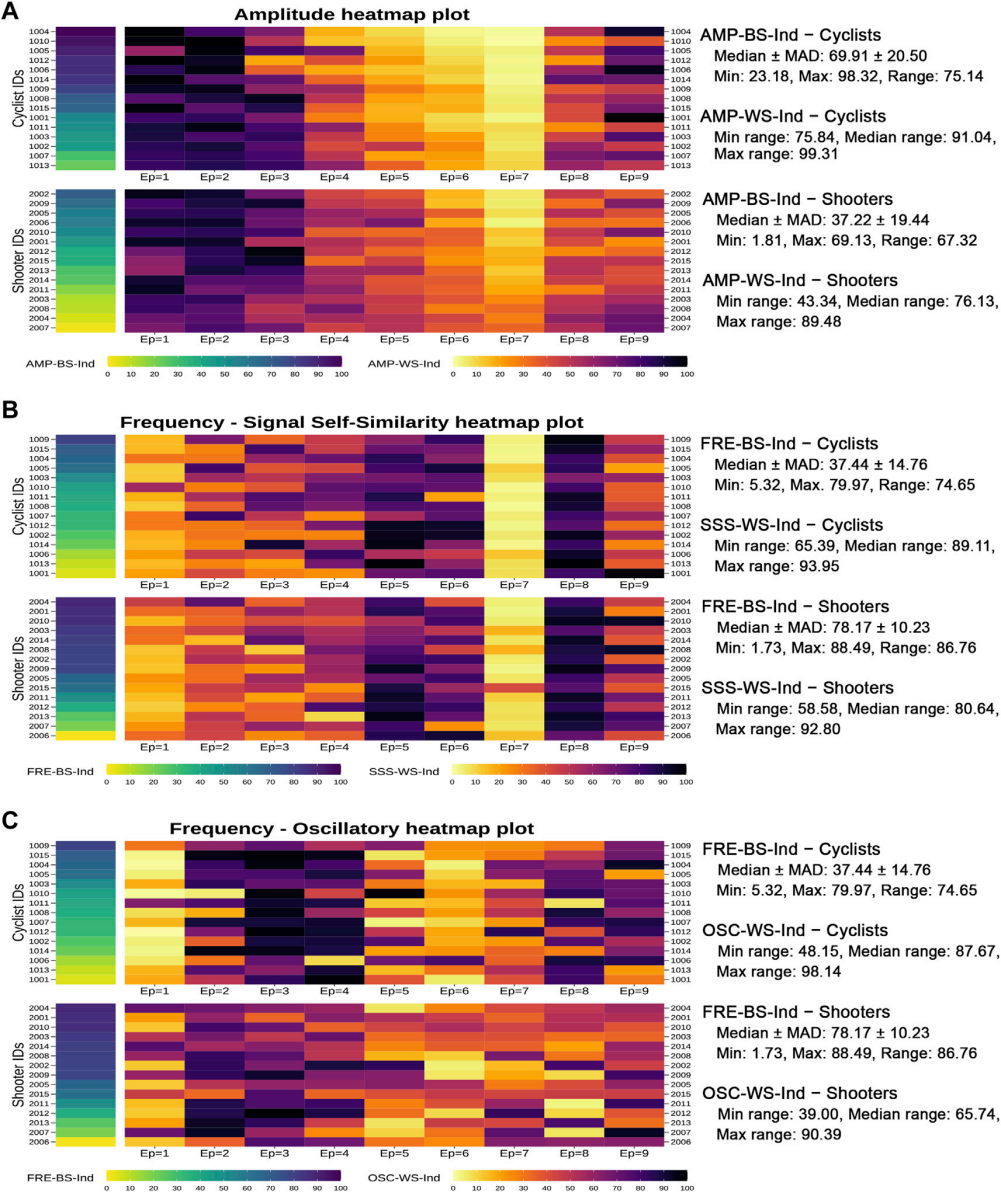
Autonomic heatmap plots of the cyclists’ and shooters’ individual autonomic profiles. *Note:* The heatmap plots in panels B and C contain the same first graphical column, which refers to the frequency BS indicator scores. The IDs of cyclists and shooters are ordered consistently with their increasing amplitude BS indicator scores (panel A) and frequency BS indicator scores (panels B-C). Some descriptive statistics within the two groups are reported on the right hand of the heatmap plots. Specifically, in the case of the two ANS-BS indicators (AMP-BS-Ind and FRE-BS-Ind), denoted generically by Y , $\text{median}\left(Y\right)\pm \text{MAD}\left(Y\right)$ , $\min\left(Y\right)$ , and $\max\left(Y\right)$ , together with $\text{range}\left(Y\right)=\max\left(Y\right)-\min\left(Y\right)$ , are computed within the cyclist and shooter groups. In the case of the three ANS-WS indicators (AMP-WS-Ind, SSS-WS-Ind, and OSC-WS-Ind), given their scores $Y_{i}$ over the nine epochs for each athlete, the range is first computed for each athlete over the epochs: $\text{range}\left(Y_{i}\right)=\max\left(Y_{i}\right)-\min\left(Y_{i}\right)$ , with i=1,…,30 . Then, the minimum, median, and maximum ranges are provided within the cyclist and shooter groups.

*Objective 3: Individual autonomic profile.* Figure 7 displays the individual profiles in the form of three autonomic heatmap plots obtained by combining, respectively, the AMP-BS and AMP-WS indicators, the FRE-BS and the SSS-WS indicators, and the FRE-BS and the OSC-WS indicators. This way, each athlete’s autonomic response to the complete test can be examined by looking simultaneously at his/her level on the specific overall amplitude or frequency domain (first column of cells) and his/her progression level on the corresponding RRV dynamic (i.e., amplitude, signal self-similarity, or oscillatory; rectangles of cells on the right). Moreover, cyclists and shooters are separated in each autonomic heatmap plot to provide clearer insights into their main differences. An interactive version of each autonomic heatmap plot in Figure 7 is reported in Supplementary Material, where individual scores can be viewed on mouseover.

The cells in Figure 7 are colored by increasing tonality according to the low/high athletes’ ANS indicator scores. As an instance of reading, in the first heatmap plot concerning the amplitude (panel A), the shooter with ID 2007 has the lowest AMP-BS-Ind score (equal to 1.81, the cell with the lightest yellow), i.e., the lowest amplitude level over the entire test. Interestingly, his/her amplitude profile over the epochs (given by the AMP-WS-Ind cells on the right) is associated with color tonality variations among the most limited ones. In other words, his/her amplitude levels tend to vary little during the test (range of ID 2007 AMP-WS-Ind scores: 43.34, the minimum observed one) compared to the whole athlete set (min range: 43.34, median range: 82.89, max range: 99.31). A similar remark holds for the shooter with ID 2004 (range of AMP-WS-Ind scores: 46.04), the athlete with the second-lowest AMP-BS-Ind score (equal to 5.66). Conversely, when moving towards higher AMP-BS-Ind scores (cells with darker colors), the athletes tend to have color tonality variations on AMP-WS-Ind among the widest ones. For instance, the cyclist with ID 1004 has the highest AMP-BS-Ind score (equal to 98.32, the cell with the darkest blue) and a variation of his/her AMP-WS-Ind scores over the epochs among the most elevated ones (range of AMP-WS-Ind scores: 98.19). In general, hence, cyclists have darker colors on AMP-BS-Ind (i.e., higher scores with median ± MAD: 69.91 ± 20.50) than shooters (median ± MAD: 37.22 ± 19.44) and stronger color tonality variations on AMP-WS-Ind over the epochs than shooters, as expressed by the highest median range over the epochs (cyclists: 91.04 vs. shooters: 76.13).

The second autonomic heatmap plot (panel B) combines frequency (FRE-BS-Ind) and signal self-similarity (SSS-WS-Ind). In contrast to amplitude, there is no visible correspondence between low/high frequency levels and low/high variations in the individual signal self-similarity profiles. As already observed (Figure 5, panel B), the shooters have higher FRE-BS-Ind levels (median ± MAD: 78.17 ± 10.23) than cyclists (median ± MAD: 37.44 ± 14.76). However, the variations observed in the SSS-WS-Ind profiles over the epochs are very similar across the two groups, as expressed by the similar min, median, and max ranges. This finding is consistent with the previous analysis results (Figure 6, panel D).

The third autonomic heatmap plot (panel C) combines frequency (FRE-BS-Ind) and oscillatory (OSC-WS-Ind). Unlike before, there is a more apparent correspondence between low/high frequency levels and high/low variations in the individual oscillatory profiles. Specifically, cyclists having lower FRE-BS-Ind levels are characterized by wider OSC-WS-Ind variations (median range: 87.67) than shooters (median range: 65.74). This finding aligns with the previous analysis results (Figure 6, panel F).

**FIGURE 8:**
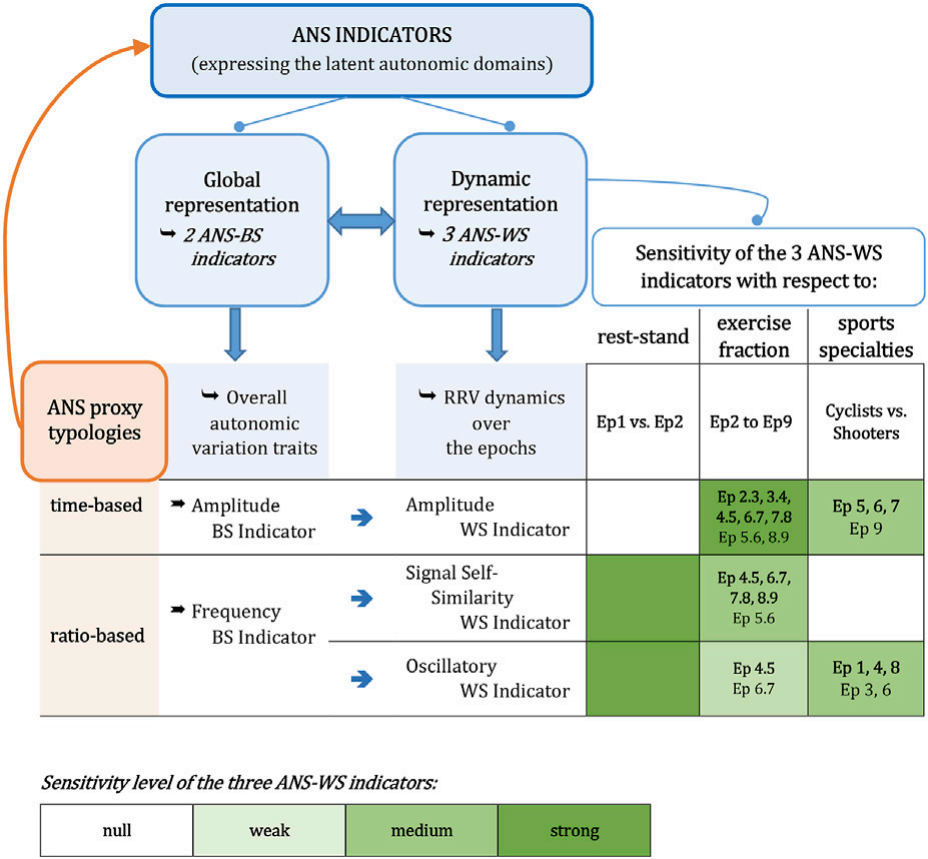
Synoptic figure summing up the meaning and main findings concerning the ANS indicators. *Note*. The sensitivity level of the ANS-WS indicators in detecting the three aspects of interest (i.e., the rest-stand postural change, the step changes in the exercise fraction, and the differences between cyclists and shooters) is based on the number of jointly significant results achieved on the considered non-parametric tests. All the details concerning such evaluations are reported in Supplementary Tables S17–S19. Specifically:−As for the changes in rest-stand and exercise fraction, the sensitivity level is evaluated over the whole athlete set through the 95% bootstrap C.I.s, the ATS-based test, and the WSR test (Supplementary Tables S17, S18). The “Ep *t.t*+1” indications reported in the green cells under the “exercise fraction” column refer to the changes from epoch *t* to epoch *t*+1 that have received at least two significant test results. In particular, bold-written “Ep *t.t*+1” indicates that all the test results are significant;−as for the cyclists vs. shooters comparison, the sensitivity level is evaluated through the overall ATS-based tests for group or group-by-epoch effects, 95% bootstrap C.I.s, and the BA, JT, KS, and StWRS tests (Supplementary Table S19). The “Ep *t*” indications reported in the green cells under the “sports specialties” column refer to the comparisons that have received at least two significant test results at epoch *t.* In particular, bold-written “Ep *t*” indicates that all the test results are significant at epoch *t.*

As a final summary, the synoptic Figure 8 reports, in essence, the methodological framework, the main findings concerning the ANS-BS and ANS-WS indicators, and the sensitivity level of the ANS-WS indicators in detecting the rest-stand postural change (epochs 1–2), the step changes in the exercise fraction (epochs 2–9), and sports specialties differences (cyclists vs. shooters). The strength of sensitivity for each ANS-WS indicator is provided by the number of concordant significant test results (see the legend below in Figure 8). Summing up, SSS-WS-Ind and OSC-WS-Ind have the strongest sensitivity in detecting the rest-stand change, while AMP-WS-Ind has no strength. Nonetheless, AMP-WS-Ind has the strongest sensitivity in detecting the athletes’ autonomic changes between every two consecutive steps in the exercise fraction, while SSS-WS-Ind has a medium sensitivity level, and OSC-WS-Ind has the weakest sensitivity. Finally, AMP-WS-Ind and OSC-WS-Ind have a medium level of sensitivity in capturing the difference between cyclists and shooters in specific epochs of the stress test, while SSS-WS-Ind has no strength.

## 4 Discussion

In this preliminary investigation, we describe the cardiac autonomic response in elite athletes to a single bout of progressive endurance exercise (epochs 3–7) and a recovery (epochs 8–9) preceded by a rest-stand test (epochs 1–2), considering both global (Between-Subjects − BS) and dynamic (Within-Subjects − WS) views of an integrated process (Malliani et al., 1991). An important aspect of exercise regards the dynamics of ANS changes with various levels of exercise intensity (from rest to recovery, as shown by the within-group median profile plots of the single ANS proxies in Figure 3), which requires an *ad hoc* method of analysis also capable of accounting for the inter-individual and intra-individual correlations (Figure 4). In particular, we observe that an integrated statistical approach based on MEFA is capable of catching the differences in ANS responses to a dynamic protocol between two different training modalities. Instead of analyzing individually the information distributed among numerous ANS indices (Figure 3), MEFA reduces the total information to two ANS-BS indicators (for the inter-individual correlations) and three ANS-WS indicators (for the intra-individual correlations), respectively. Such indicators provide two different, though complementary, representations of the autonomic regulation of the sino-atrial node by capturing the latent autonomic domains underlying the complete test of the dynamic protocol (Figure 8). These findings should be combined with the compelling observation, derived from electroneurographic experiments, that changes in the relative balance between oscillations may serve as a marker of functional, inhibitory/excitatory states (Pagani et al., 1997). This effect may be reached through changes in the average activity of vagal and sympathetic central neurons but also the balance between LF and HF oscillations, thus emphasizing the different nature of multiple coding modalities, like, in this case, amplitude and oscillatory codes.

### 4.1 Criteria adopted for selecting the studied ANS proxies

One crucial aspect of the study was the selection of the ANS proxies. Thousands of studies have addressed HRV as a proxy of vagal and sympathetic control, utilizing various algorithms and experimental models and considering myriad HRV variables, which “applied in individual studies hinders easy and reliable comparisons of methods and results. Some of the indices are redundant, and others discriminative only with respect to particular influences or disturbances” (Hoyer et al., 2019). Related-HRV indices may be organized in categories reflecting specific fields of application: e.g., in ANS development (amplitude, complexity, and patterns) (Hoyer et al., 2019), in cardiovascular clinics (from simple statistics to non-linear estimates) (Malik and Camm, 1993; Task Force of the European Society of Cardiology and the North American Society of Pacing and Electrophysiology, 1996), or specific applications like the physiology of exercise (low, moderate, and elevate intensity). The still ongoing debate relates to the importance of various parameters of HRV, which continued to grow in indices and clinical relevance, and the capacity of HRV to better reflect autonomic tone (signal amplitude) or responsiveness (phasic/oscillatory activity) (Malik and Camm, 1993).

In the early 1980s, we started to find a way to assess autonomic evaluation from RRV based on a dual feedback model (Pagani et al., 1986; Malliani et al., 1991) suggested by experiments demonstrating the existence of positive feedback reflexes and through a simple monovariate approach utilizing RRV short-term and autoregressive (parametric) spectral analysis. With bioengineering terms, we considered HR, time and frequency domain indices, and linear/non-linear models that were approximately divided into vagal (HF) and sympathetic (LF) linked parameters according to a duality in neural circuitry (Schwartz et al., 1973). In order to account for major non-linearities of the exercise protocol and maintain a direct appreciation of novel techniques, we combined the indices we started with (Pagani et al., 1986) with newer ones (non-linear, complexity, symbolic, and phased rectified signal average) (Hoyer et al., 2019), so as to select the 12 ANS proxies in Table 1. In particular, eight are the unitary variables we usually consider in our laboratory (both as amplitude and as normalized units: HR, RR RMS and RMSSD, RR TP, LF, and HF; these latter two are traditionally both amplitude and purely ratio-based). The other considered four variables derived from novel techniques: the phase rectified indices AC and DC (Bauer et al., 2006) and two representations from entropy (RR Ro) and symbolic dynamic (P0v) categories (Porta et al., 2001). As pointed out in Section 2.2, we distinguished these 12 ANS proxies into the two “time-based” and “ratio-based” variable typologies (Table 1) to differentiate the proxies preserving the time measurement unit (“time-based”) from the proxies expressed in normalized numbers (“ratio-based”); this dichotomy can be regarded as a broad fitting to the hypothesis of two neural coding modalities: amplitude and frequency (Pagani et al., 1997).

Besides this, several statistical technical questions were considered in selecting the 12 ANS proxies. Firstly, factor analysis methods (such as MEFA) require the presence of a *sufficient level* of multicollinearity (i.e., roughly medium/high correlations) among the input observed variables, as reflected by the KMO index (see the note below Figure 4), to achieve satisfactory results in terms of latent constructs. The more the observed variables are correlated, the better the results derived from the application of factor analysis will be in terms of data dimensionality reduction (i.e., a few latent factors capable of reproducing the observed correlations with limited information loss) and interpretability of the extracted latent factors (i.e., latent factors highly correlated with a few, possibly distinct observed variables). Consequently, selecting these specific ANS proxies gave *a priori* internal consistency to the set of input variables and allowed us to obtain a few statistical indicators with clear meaning and high cumulative percentages of reproduced (BS and WS) total variance (Tables 3-4). Secondly, we confined our selection to no more than 12 ANS proxies to avoid numerical problems in executing the statistical procedures, particularly the bootstrap. Given the small size of the athlete set ( n=30 ), numerical problems already occurred in the BS analysis (see the note below Table 3), while the WS analysis did not experience this issue because it is based on a higher number of observations ( nT=270 ) (Section 2.3; Figure 2).

### 4.2 An integrated statistical approach based on MEFA and ANS

This MEFA application has yielded a more straightforward representation of the complexity that characterizes ANS modulation during stand and exercise by reducing the total information to two ANS-BS indicators and three ANS-WS indicators. The ANS-BS indicators, deriving from the averaged ANS proxies (Figure 2), give a *global representation* of the athletes’ autonomic characteristics, expressing the main overall athletes’ autonomic variation traits over the entire test. These indicators are also used to discover the autonomic traits that might distinguish cyclists and shooters globally. The ANS-WS indicators, deriving from the within-athletes-centered ANS proxies (i.e., ANS proxies with values adjusted for each athlete’s means computed over the epochs, Figure 2), allow for a representation of the athletes during the complete test net of their overall magnitudes reached on the ANS proxies. Hence, they can provide a *dynamic representation* of the RRV mechanisms underlying the entire test independently of the athletes’ overall ANS proxy averages and, accordingly, can be used to compare cyclists and shooters based on such obtained RRV dynamics. In this sense, it is not contradictory to see overall higher amplitude BS indicator levels in cyclists than shooters (Figure 5, panel A) and, at the same time, the cyclists’ blue profile of the amplitude WS indicator significantly more shifted toward lower median scores at epochs 5–7 than the shooters’ red profile, without other significant differences at the remaining epochs (excepted epoch 9, Figure 6, panel B. See also Supplementary Figure S4).

Besides that, several crucial aspects regarding these indicators and the statistical methodology employed for their construction are worth stressing. Firstly, for reasons similar to those advanced in Solaro et al. (2021a), the ANS indicators we built through the MEFA application and the PKDE transformation are not to be intended as *measurement indicators* of the various aspects involved in the ANS control during exercise, e.g., the magnitude of the ANS changes over the exercise epochs. Constructing measurement indicators requires, first of all, the availability of larger sets of subjects representing more comprehensive ranges of different characteristics. Moreover, roughly speaking, the metric property inherent in the original ANS proxies should be conveyed to the statistical indicators as far as possible to have actual measurement indicators. On this point, one can assume that the extracted common factors represent a new coordinate system of smaller dimensions than the one given by the original observed variables, on which basis subjects can be inspected more readily. In this sense, the common factors represent a sort of new metric system in which the original values of variables are replaced and condensed by factor scores. Let us assume that this new coordinate system well represents the subjects (e.g., roughly, subjects with similar variable values should have similar factor scores or subjects with very different variable values should have very different factor scores). If transformations like the ones based on cumulative distribution functions, such as PKDE, are applied to factor scores, then the rank order of scores does not change, but the intervals between scores do. In particular, small differences at the center of the factor distributions tend to be amplified, while large differences in the distribution tails tend to be compressed (see the remarks by Murphy and Davidshofer, 2004, chap. 5, on the “area transformations”). In other words, the transformation we applied to the ANS latent factors does not preserve the metric system induced by the ANS latent factors. Nonetheless, within the scope of this preliminary investigation, we aimed, above all, to capture the ANS changes over the epochs by representing the main latent autonomic domains with fewer indicators and, on these bases, to compare two athlete groups characterized by different training modalities. From this point of view, the ANS indicators we built are to be regarded more appropriately as *process indicators* capable of capturing the transitions of the ANS control from one epoch to another during the entire test and between different training modalities.

Secondly, MEFA turned out to be a very flexible methodology to meet the objectives listed in the Introduction and Section 2.3. Unlike traditional dimensionality reduction techniques such as EFA or Principal Component Analysis (PCA), MEFA is capable of providing a variance decomposition into sources of variation linked to the various levels of a hierarchical data structure. In this study, having a two-level data structure, we provided a decomposition of the athletes’ individual information into a between-athletes variation and within-athletes epoch variation, from which two different representations (global and dynamic) of the athletes’ cardiac autonomic response to the complete test have been derived. Moreover, unlike PCA and similar to EFA (of which MEFA is an extension), MEFA meets the primary goal of the factor analysis methods, i.e., seeking the latent causes (expressed by the common latent factors) that could explain the linear relationships among the observed variables. In our study, such linear relationships are expressed by the BS and WS correlation matrices (Figure 4), for which two sets of ANS-BS and ANS-WS latent factors were derived (Tables 3–4) [see the corresponding factor models (14)–(15) in Methodological Appendix, Supplementary Material].

Thirdly, strictly related to the above remarks, MEFA shares the same flexibility as EFA in extracting latent factors with specific statistical properties, such as the uncorrelation of the ANS latent factors we obtained. The principal factor (or also principal axis) extraction method, along with the varimax rotation (or variance maximizing rotation), is the most conventional technique in factor analysis, which allows for the extraction of uncorrelated common factors such that they can be more easily interpreted based on their loadings with the observed variables (Finch, 2020). Simplifying the interpretation of the ANS-BS and ANS-WS latent factors derived in a preliminary investigation was our main reason in favor of the uncorrelation property. However, it is generally possible to obtain correlated common factors by applying alternative rotation methods, such as the “oblique rotations” (e.g., the oblimin rotation, which is widely used in psychometrics), instead of “orthogonal rotations,” such as the varimax rotation (Finch, 2020), or even to obtain statistically independent factors by applying Independent Component Analysis (ICA), which, despite being an alternative dimensionality reduction technique, can be regarded as another factor rotation method (Hastie, Tibshirani, and Friedman, 2009, chap. 14).

As a final consideration, the ANS indicators used in the study are the extracted BS and WS latent factors to which the PKDE transformation was applied. Since the PKDE is not a linear transformation, from a theoretical point of view, there is no certainty that the obtained indicators have correlations precisely equal to zero (or numerically very close to zero). It might occur that, after transformation, the correlation coefficients slightly increase in absolute value. Nonetheless, their values are typically of small magnitude and then statistically negligible, as we checked in our case.

### 4.3 The bicycle exercise: Global and dynamic representations of cardiac autonomic regulation

Exercise is a powerful excitatory stimulus to ANS, which may be examined non-invasively utilizing several autonomic indices that are frequently assessed individually. For example, the dynamics of the increase in the sympathetic drive during exercise could be assessed by the NU power of the LF component of RRV, at least to an extent (Rimoldi et al., 1990; Casadei et al., 1995; Lucini et al., 2004). Our novel approach provides two major advancements, i.e., a global and a dynamic representation of the athletes’ cardiac autonomic response to exercise with few informationally rich statistical ANS indicators.

In the global representation, the ANS-BS indicators, representative of the entire test, are limited to two domains, i.e., amplitude (linked to the time-based proxies) and frequency (linked to the ratio-based proxies) (Table 1), and carry about 39% and 29% of the BS total variance, respectively (Table 3). As shown in Figure 5, cyclists globally have significantly higher amplitude and lower frequency levels than shooters. Hence, considering the two different sports specialties as extremes of global components, the overall profiles of amplitude and frequency are accordingly different: prevailing amplitude in cyclists (vagal/parasympathetic predominance) and prevailing frequency in shooters (sympathetic predominance), a part of which is constituted by a high LF oscillatory component. This difference between the groups represents most likely the effects of long-term physiological remodeling (Oggionni et al., 2021) due to the different loads of endurance training, as exemplified by cyclists and shooters (Mitchell et al., 2005).

Low-frequency neural rhythms may also exert influences outside the cardiovascular domain, e.g., affecting fine muscular control (Lodha and Christou, 2017). Stress and sympathetic drive may also influence these rhythms, possibly through the locus coeruleus (Mather et al., 2017) or other central autonomic nuclei.

Regarding the dynamic representation, Figure 6 synthesizes the profiles of the single 12 ANS proxies represented in Figure 3 using only the three obtained ANS-WS indicators, i.e., the amplitude, signal self-similarity, and oscillatory WS indicators, which reproduce, respectively, nearly 46%, 18%, and 13% of total WS variance (Table 4). This graphical representation of the median profiles built over the whole athlete set (first column of panels) and within the cyclist and shooter groups (second column) furnishes a novel representation of the exercise dynamics described as a response to posture, several steps of exercise, and recovery. Notably, the percentages of the maximal heart rate (reached at epoch 7) obtained at epochs 3 and 4 (Table 2) suggest that both cyclists and shooters were exercising at low-moderate intensities (corresponding to a prevalent aerobic metabolic pathway (Pelliccia et al., 2021; Table 4)), while the percentages at epochs 5 and 6 suggest that they were exercising at high or very-high intensities (corresponding to a prevalent anaerobic metabolic pathway (Pelliccia et al., 2021; Table 4)).

By focusing on the whole athlete set, we first observe that the rest-stand response (epoch 1 vs. epoch 2) is characterized by no change in the amplitude indicator (Figure 6, panel A), a moderate increase in the signal self-similarity indicator (panel C), and a marked shift from low to high in the oscillatory indicator (panel E). The performed tests indicate that the signal self-similarity and oscillatory indicators have a strong sensitivity level in capturing the rest-stand transition, while amplitude has no strength (Figure 8).

Particular trends are observed for the exercise fraction (epochs 2–9). First, in amplitude (Figure 6, panel A), there is a graded stepwise reduction from a high level in stand to nearly zero at peak exercise and then an increase in the recovery steps, according to a U-like trend. This indicator has the strongest sensitivity level to exercise bout; it is the most responsive to the athletes’ autonomic changes between every two consecutive stress test steps (Figure 8). In signal self-similarity (panel C), a wave-like trend is observed with a dip at peak exercise. This indicator has a medium sensitivity level because it recognizes the athletes’ autonomic changes at every two consecutive steps starting from moderate-intensity exercise (epoch 4) to the last recovery step (epoch 9) (Figure 8). In oscillatory (panel E), we observe an inverted U-like trend in the first six epochs, followed by a monotonic increase until the last recovery step. This indicator has the lowest sensitivity level; it signals only fewer step transitions, in particular, the transition from moderate to high intensity (epochs 4–5) and from very high intensity to peak exercise (epochs 6–7) (Figure 8).

Regarding the comparisons between cyclists and shooters, changes in the amplitude and oscillatory WS indicators are different in the two athletes’ groups across the dynamic protocol, being more evident in cyclists (Figure 6, second column of panels). In particular, the progressive reduction of the amplitude WS indicator during exercise epochs and its increases in the recovery phases are more pronounced in cyclists (Figure 6, panel B), thus suggesting that high-intensity endurance training is characterized by more evident dynamic changes in parasympathetic control. Also, the increase of the oscillatory WS indicator during the first phases of exercise (characterized by a prevalent aerobic metabolism, Table 2) is more evident in cyclists, suggesting that high-intensity endurance training is more characterized by an evident sympathetic activation during aerobic exercise (Figure 6, panel F).

Of particular clinical interest is this capability of the oscillatory indicator to catch the different response profiles of two different training modalities: Athletes with a prevalent high-intensity endurance training (cyclists) present an evident increase on this indicator in the first (epochs 3–4) (aerobic) steps of exercise, a paradoxical reduction at epochs 5–7 (characterized by a prevalent anaerobic metabolism) (Pelliccia et al., 2021), and an increase during recovery, while athletes with a less endurance training present from the beginning of exercise a paradoxical reduction on this indicator (Figure 6, panel F). Other papers present in the literature (Casadei et al., 1995; Lucini et al., 2004) show a progressive increase of LFnu in normal subjects performing a low intense exercise (from 10% to 30% of maximal heart rate), and many papers show that aerobic endurance (long-term) training is capable of positively affecting ANS control (Joyner and Green, 2009; Lucini et al., 2020a) inducing a shift toward a prevalent parasympathetic control. These findings point out the importance of aerobic endurance training (not maximal prevalent anaerobic exercise) in modulating ANS control and the capability of autoregressive HRV to depict this clinical effect.

The lowest sensitivity level of the oscillatory WS indicator achieved in the exercise fraction (Figure 8) and the above-mentioned paradoxical reduction pattern need a special comment. This indicator increases with orthostatic stimulus in both athletes’ groups; during the first steps (aerobic endurance exercise), it increases in cyclists while it starts decreasing in shooters; during high intensities-maximal exercise steps (prevalent anaerobic endurance exercise), it paradoxically decreases in both athletes’ groups, being more evident in cyclists; then it increases during recovery (Figure 6, panel F). This paradoxical pattern is obviously evident also considering the single ANS variables derived from frequency analysis of HRV (Figure 3), and it was already observed by other researchers (Casadei et al., 1995), stimulating a great discussion regarding the usefulness of variables (in particular LFnu) derived from autoregressive HRV frequency domain in describing sympathetic responses to high-intensity exercise. Moreover, similar behavior of LFnu is observed in athletes performing strength exercises (exercise modality typically characterized by prevalent anaerobic metabolism) (Iellamo et al., 2019). Also, heart failure patients present similar paradoxical reduction variables derived from frequency analysis of HRV (in particular LFnu) (Van de Borne et al., 1997), while more direct ANS measures, such as MSNA (Katayama and Saito, 2019), show elevated overall sympathetic activity. This paradoxical pattern may be, albeit only in part, explained considering that conditions characterized by high levels of sympathetic activity (such as high intensity/maximal exercise or heart failure) present an extreme afferent involvement from the periphery (muscle reflexes, chemoreflexes, hyperventilation, etc.), which disturb the complex interaction of the multiple mechanisms involved in determining the final rhythms that are analyzed using the autoregressive spectral analysis approach (Malliani et al., 1991). However, the clear, dynamic pattern and its differences between the two athlete groups, characterized by different endurance load training, corroborate the importance of considering this indicator as a useful parameter to depict the ANS responses during exercise. In fact, the momentum when it paradoxically decreases with the increase of exercise load differs per the two athlete groups (Figure 6, panel F).

Nonetheless, using more complex analyses that also consider non-linear variables may offer an opportunity to manage, albeit in part, this pitfall (Porta et al., 2001). Moreover, the combination of the high-frequency rate of spikes with the critical ephaptic transmission of unmyelinated fibers might determine a narrow bandwidth performance. In the present study, the signal self-similarity WS indicator identified by the MEFA approach aggregates the HRV indices P0v and RR Ro derived from the non-linear pattern analysis and complexity analysis (Porta et al., 2001). This indicator (Figure 6, panels C–D) progressively increases from rest to high-intensity exercise (epoch 5), then drastically (and paradoxically) decreases with maximal exercise (epoch 7). It seems more robust in suggesting the physiological increases of sympathetic outflow during exercise than the oscillatory WS indicator (Figure 6, panels E–F), which aggregates the HRV frequency-domain variables RR LFnu (considered a marker of prevalent sympathetic modulation to the sino-atrial node) and RR HFnu (considered a marker of prevalent vagal modulation to the sino-atrial node) derived from the linear analysis.

### 4.4 Autonomic heatmap plots

A further in-depth statistical analysis offers the possibility to depict the individual dynamic response to orthostatic or exercise stimuli. In fact, a deeper perusal of data considering the athletes’ individual autonomic profiles indicates clearly that the two athlete groups respond differently to bicycling stress. These profiles are well described by color-coded, autonomic heatmap plots (Figure 7), which, combining the global and the dynamic representations of the ANS indicators, allow for a complete description of the athletes’ autonomic response to the entire test evidencing changing amplitude and frequency (in its signal self-similarity and oscillatory components) over time.

In particular, the amplitude dynamic underlying the response to exercise (Figure 7, panel A) appears with the AMP-BS scores that tend to be higher in cyclists (darker colors in the first cell column). Then, the exercise steps show higher AMP-WS scores for cyclists at rest and stand (darker colors) that rapidly transform into lower scores (lighter colors) at peak exercise, followed by a rapid (albeit incomplete) recovery (darker colors). Overall, cyclists’ amplitude profiles appear to have higher variations than shooters.

The frequency dynamic is shown subdivided into its two components, signal self-similarity and oscillatory, in the heatmap plots in panels B and C, respectively. Regarding panel B, no visible difference in changing signal self-similarity over time is appreciated between cyclists and shooters, although shooters tend to have higher FRE-BS scores (darker colors in the first cell column). Therefore, this heatmap plot reveals the part of the overall frequency domain not sensitive to cyclist and shooter differences. In contrast, in the frequency-oscillatory dynamic (panel C), cyclists appear with lower FRE-BS scores (lighter colors in the first cell column) and wider OSC-WS variations than shooters (colors with greater tonality changes). Therefore, this heatmap plot evidences the part of the overall frequency domain sensitive to cyclist and shooter differences. Notably, the oscillatory WS indicator appears particularly sensitive to standing up, which is substantially not signaled by the other two amplitude and signal self-similarity WS indicators. This behavior suggests that the non-linear profile of LFnu with increasing intensity of exercise rather than a flaw of the algorithm is a reflection of an intrinsic non-linear code.

All the above indicates that different athletic fitness translates into different athletic phenotypes characterized by different mechanisms underlying the autonomic response to standing up and exercise. Such an autonomic response should then be more adequately intended as an overall reaction to the exercise in its entirety, on the one hand, and in its single dynamics unfolding during the exercise execution, on the other hand. In particular, the difference between rest (epoch 1) and peak exercise (epoch 7) in the amplitude WS indicator (panel A) may be taken as the essence of the individual autonomic response to exercise, which might be viewed as a proxy of increasing performance.

### 4.5 Study limitations

This study presents some limitations.

Firstly, the study population is limited to a small set of athletes. Nevertheless, they are elite athletes representing the extreme in endurance training loads: prevalent high-intensity endurance training (cyclists) and prevalent technical training with a low-intensity endurance component (shooters).

Secondly, the clinical routine did not comprise a cardiopulmonary stress test and/or lactate evaluation to precisely define the anaerobic threshold and then the exercise steps characterized by prevalent aerobic or anaerobic metabolism. However, for each subject, we calculated the value corresponding to the percentage of maximal heart rate (reached at epoch 7) (Table 2; Supplementary Table S6) and, referring to ranges reported by international guidelines (Pelliccia et al., 2021), we verified that the exercise performed in epochs 3 and 4 could be considered of low/moderate intensity (prevalent aerobic metabolism) and the exercise performed in epochs 5, 6, and 7 could be considered of high/very high/maximal intensity (prevalent anaerobic metabolism).

Thirdly, autoregressive spectral analysis of HRV does not “measure” nerve activity but provides indirect indices of sino-atrial autonomic control. On the other hand, this methodology nowadays may be considered the *de facto* methodology (Shaffer and Ginsberg, 2017) to study cardiac autonomic control non-invasively. Moreover, the use of advanced algorithms (considering linear and non-linear indices) and advanced statistics, such as the definition of statistical indicators based on MEFA, contribute to corroborating the validity of using this technique in the clinical field.

Finally, we only studied the autonomic response of heart rate and postponed our interest to other important targets, in particular arterial vessels. This may be a valuable topic, as documented by a few investigations on exercise in humans (Lucini et al., 2004) or dogs (Rimoldi et al., 1992), whereby the reduction of LF power of RRV with increasing exercise intensity is associated with an increase of LF power of arterial pressure variability and a reduction of LF of RRV. The availability of multiple signals (RRV and Arterial Pressure Variability) may also permit the analysis of complex closed-loop control mechanisms, like baroreflexes (Baselli et al., 2001).

## 5 Conclusion

This study offers a novel view regarding the importance of the autoregressive spectral analysis of HRV as a non-invasive methodology to describe the dynamic of exercise responses, mainly focusing on the differences between exercise levels characterized by prevalent aerobic or anaerobic metabolic pathways, thus rendering this methodology well-suited to a clinical endeavor. The application of an integrated data-driven and non-parametric statistical approach based on MEFA permits a more straightforward representation of the complexity that characterizes ANS modulation during exercise, summarizing and differentiating the different contributions of many HRV-derived indices and simplifying the interpretation of results. In this sense, the proposed approach may be regarded as a novel way to consider the spectral analysis of RRV during exercise, which aims at overcoming its limits and dealing with the possible inconsistencies of some indices (e.g., the paradoxical reduction of LFnu in high-intensity exercise) observed by several researchers (e.g., Casadei et al., 1995). MEFA can detect, synthesize, and separate the total information content into common latent factors that, through a convenient transformation, can be expressed in statistical process indicators capable of being analyzed separately since they are set up to be uncorrelated. This procedure represents a sort of *a posteriori* treatment of the HRV-derived indices in assessing the athletes’ autonomic response during incremental exercise, allowing for a better comprehension of the RRV dynamics, including those deriving from the most debated ANS proxies, and more immediate comparisons among athletes from different sports disciplines, thus facilitating the possible real-life and clinical use of this non-invasive methodology. This approach based on MEFA has, in fact, the potential to be applied in all clinical contexts where a multiplicity of quantitative variables is repeatedly observed, e.g., over time, and the objective is to study the evolution of specific pathological and physiological subjects’ conditions (e.g., in the presence of administered treatments or various interventions) through the construction of synthetic statistical indicators.

Of particular interest is the issue of how coaches and athletes could implement the study of the autonomic nervous system using HRV in their everyday practice. Nowadays, this methodology might be helpful to detect the ANS modifications during routine training (Lucini et al., 2021), to define different ANS profiles characterizing different training routines corresponding, for instance, to different roles in soccer (Lucini et al., 2020b), and to show the benefic effect of mental training to manage stress in female elite soccer players (Pagani et al., 2023). The complex approaches to HRV analysis employing *ad hoc* statistics also seem (Lucini et al., 2018) to be helpful: “*to understand the differences in autonomic regulation between excellent athletes and those skilled enough to qualify for the Olympics*” (Miglis and Muppidi, 2018). In this study, we presented a further step in these directions using an advanced statistical analysis approach for constructing statistical indicators, which might help translate the non-invasive study of ANS employing HRV into sport everyday practice.

## Data Availability

The original contributions presented in the study are included in the article/Supplementary Material. The raw data supporting the conclusions of this article are not readily available because, though wholly anonymized, they refer to a small set of Italian Olympic athletes who might be recognized given their unique characteristics. Requests to access the dataset should be directed to the corresponding author.

## Abbreviations

AMP-BS-Ind, Amplitude Between-Subjects Indicator; AMP-WS-Ind, Amplitude Within-Subjects Indicator; FRE-BS-Ind, Frequency Between-Subjects Indicator; OSC-WS-Ind, Oscillatory Within-Subjects Indicator; SSS-WS-Ind, Signal Self-Similarity Within-Subjects Indicator.
