# Supplementary material for: Assessing the cardiac autonomic response to bicycle exercise in Olympic athletes with different loads of endurance training: new insights from statistical indicators based on multilevel exploratory factor analysis
Source: Front Physiol. 2023 Oct 17;14:1245310. doi: 10.3389/fphys.2023.1245310 (PMC10616979; doi:10.3389/fphys.2023.1245310)
Supplement: Supplementary file 2 [file Presentation1.PDF]

Supplementary Material

Assessing the cardiac autonomic response to bicycle exercise in Olympic athletes with different loads of endurance training: New insights from statistical indicators based on multilevel exploratory factor analysis

Nadia Solaro, Massimo Pagani, Antonio Spataro, Daniela Lucini*

*** Correspondence:** Daniela Lucini: daniela.lucini@unimi.it

1 Methodological Appendix

1.1 Multilevel Exploratory Factor Analysis

The Multilevel Exploratory Factor Analysis (MEFA) (Härnqvist, 1978; Hox, 1993; Reise et al., 2005) can be regarded as a generalization of the standard one-level Exploratory Factor Analysis (EFA) (Finch, 2020) for data with a nested or hierarchical structure. This type of structure intends that a set of elementary statistical units (so-called level-1 units) is observed within categories or groups (level-2 units) that, in turn, are classified into macro-categories or macro-groups (level-3 units) and so on. MEFA can also be viewed as a purely exploratory factorial approach in the more general class of multilevel covariance structure models (Muthén, 1991, 1994).

Within the scope of the present work, we provide methodological details on MEFA regarding a two-level repeated measure data structure, where the level-2 units are represented by a population of n subjects, and the level-1 units are given by the time points on which observations are collected for each subject. Specifically, let $\boldsymbol{X} = (X_1, \ldots, X_p)'$ be a vector of p quantitative variables X_j with overall mean vector $\mathrm{M}(\boldsymbol{X}) = \boldsymbol{\mu} = (\mu_1, \ldots, \mu_j, \ldots, \mu_p)'$ and covariance matrix $\mathrm{V}(\boldsymbol{X}) = \boldsymbol{\Sigma}_{\mathrm{T}}$ [1]. These variables are repeatedly observed on the n subjects for T_i time points each, $i = 1, \ldots, n$. The collected

[1] $'$ is the transposition symbol.

observations x_{tij}, which are $N=\sum_{i=1}^{n}T_i$ in all[2], are organized in p-dimensional individual vectors $\mathbf{x}_{ti}=\left(x_{ti1}\ldots x_{tij}\ldots x_{tip}\right)'$, with $t=1,\ldots,T_i$ and $i=1,\ldots,n$. Accordingly, matrix $\boldsymbol{\Sigma}_{\mathrm{T}}$ is defined as:

$$\boldsymbol{\Sigma}_{\mathrm{T}}=\mathrm{V}(\boldsymbol{X})=\frac{1}{N}\sum_{i=1}^{n}\sum_{t=1}^{T_i}(\mathbf{x}_{ti}-\boldsymbol{\mu})(\mathbf{x}_{ti}-\boldsymbol{\mu})'.$$

The starting theoretical point is the decomposition of the total covariance matrix $\boldsymbol{\Sigma}_{\mathrm{T}}$ into two matrices, one representing the variability at the inter-individual level, i.e., the between-subjects (BS) variability, and the other representing the variability at the intra-individual level, i.e., the within-subjects (WS) variability. Formally, the decomposition is:

$$\boldsymbol{\Sigma}_{\mathrm{T}}=\boldsymbol{\Sigma}_{\mathrm{B}}+\boldsymbol{\Sigma}_{\mathrm{W}}, \tag{1}$$

where $\boldsymbol{\Sigma}_{\mathrm{B}}$ is the BS covariance matrix and $\boldsymbol{\Sigma}_{\mathrm{W}}$ is the WS covariance matrix.

The main objective of MEFA is to derive two small sets of common latent factors through which $\boldsymbol{\Sigma}_{\mathrm{B}}$ and $\boldsymbol{\Sigma}_{\mathrm{W}}$ can be reproduced with the possible minor loss of information. To this aim, the basic conceptualization is that vector $\boldsymbol{X}$ can be decomposed into three additive components:

$$\boldsymbol{X}=\boldsymbol{\mu}+\boldsymbol{X}_{\mathrm{B}}+\boldsymbol{X}_{\mathrm{W}}, \tag{2}$$

where in (2), besides the overall mean vector $\boldsymbol{\mu}$, $\boldsymbol{X}_{\mathrm{B}}$ and $\boldsymbol{X}_{\mathrm{W}}$ represent two different contributions to the vector $\boldsymbol{X}$. Specifically, $\boldsymbol{X}_{\mathrm{B}}$ is the BS component; it describes the subjects' global contribution over all the time points as expressed by the differences between the within-subject means $\bar{\mathbf{x}}_i$ and the overall mean $\boldsymbol{\mu}$: $\bar{\mathbf{x}}_i-\boldsymbol{\mu}=[\bar{x}_{.ij}-\mu_j]'_{j=1,\ldots,p}$, with $\bar{x}_{.ij}=\frac{1}{T_i}\sum_{t=1}^{T_i}x_{tij}$, for all $i=1,\ldots,n$. The variability of $\boldsymbol{X}_{\mathrm{B}}$ is thus represented by matrix $\boldsymbol{\Sigma}_{\mathrm{B}}$ in (1), which is given by:

$$\boldsymbol{\Sigma}_{\mathrm{B}}=\mathrm{V}(\bar{\boldsymbol{X}})=\frac{1}{N}\sum_{i=1}^{n}(\bar{\mathbf{x}}_i-\boldsymbol{\mu})(\bar{\mathbf{x}}_i-\boldsymbol{\mu})'T_i, \tag{3}$$

where $\bar{\boldsymbol{X}}=(\bar{X}_1,\ldots,\bar{X}_j,\ldots,\bar{X}_p)'$ is the p-dimensional vector of the within-subject mean variables $\bar{X}_j$ with values $\bar{x}_{.ij}$, mean $\mathrm{M}(\bar{X}_j)=\mu_j$, and variance $\mathrm{Var}(\bar{X}_j)=\sigma^2_{\mathrm{B}j}=\mathrm{Var}_{\mathrm{B}}(X_j)$, i.e., the BS variance of

[2] In the balance case, where $T_i=T$ for all $i=1,\ldots,n$, the total number of observations is given by $N=nT$.

variable X_j, $j=1,\ldots,p$. Definition (3) highlights more clearly that the BS component refers to the between-individual mean differences and thus expresses the variability at the inter-individual level.

On the other hand, $\boldsymbol{X}_{\mathrm{W}}$ in (2) is the WS component; it represents the subjects' contribution at every specific time point through the differences between the individual vectors $\mathbf{x}_{ti}$ and the within-subject means $\overline{\mathbf{x}}_i$: $\mathbf{x}_{ti}-\overline{\mathbf{x}}_i=[x_{tij}-\overline{x}_{.ij}]'_{j=1,\ldots,p}$, for all $t=1,\ldots,T_i$ and $i=1,\ldots,n$. Let $\boldsymbol{X}_i=(X_{i1},\ldots,X_{ij},\ldots,X_{ip})'$ be the p-dimensional vector of the variables X_{ij} having values x_{tij}, with mean $\mathrm{M}(X_{ij})=\overline{x}_{.ij}$ and variance $\mathrm{Var}(X_{ij})=\sigma^2_{ij}$, for each $i=1,\ldots,n$. Each vector $\boldsymbol{X}_i$ has its own covariance matrix $\mathrm{V}(\boldsymbol{X}_i)$ expressing the variability of subject i over the T_i time points:

$$\mathrm{V}(\boldsymbol{X}_i)=\boldsymbol{\Sigma}_i=\frac{1}{T_i}\sum_{t=1}^{T_i}(\mathbf{x}_{tj}-\overline{\mathbf{x}}_i)(\mathbf{x}_{tj}-\overline{\mathbf{x}}_i)', \qquad i=1,\ldots,n.$$

Matrix $\boldsymbol{\Sigma}_{\mathrm{W}}$ in (1) is then defined as the pooled average of the covariance matrices $\mathrm{V}(\boldsymbol{X}_i)$:

$$\begin{aligned}\boldsymbol{\Sigma}_{\mathrm{W}}&=\frac{1}{N}\sum_{i=1}^{n}\boldsymbol{\Sigma}_iT_i=\frac{1}{N}\sum_{i=1}^{n}\left\{\frac{1}{T_i}\sum_{t=1}^{T_i}(\mathbf{x}_{tj}-\overline{\mathbf{x}}_i)(\mathbf{x}_{tj}-\overline{\mathbf{x}}_i)'\right\}T_i=\\&=\frac{1}{N}\sum_{i=1}^{n}\sum_{t=1}^{T_i}(\mathbf{x}_{tj}-\overline{\mathbf{x}}_i)(\mathbf{x}_{tj}-\overline{\mathbf{x}}_i)',\end{aligned} \tag{4}$$

so that $\boldsymbol{\Sigma}_{\mathrm{W}}$ is also said "pooled-within-subject" covariance matrix. In particular, the j-th element on the diagonal of $\boldsymbol{\Sigma}_{\mathrm{W}}$ is the WS variance of variable X_j, which is given by the pooled average of variances $\mathrm{Var}(X_{ij})=\sigma^2_{ij}$, i.e.: $\mathrm{Var}_{\mathrm{W}}(X_j)=\sigma^2_{\mathrm{W}j}=\frac{1}{N}\sum_{i=1}^{n}\sigma^2_{ij}T_i$, $j=1,\ldots,p$. Definition (4) highlights more clearly that the WS component refers to within-individual differences, i.e., individual differences at each time point with respect to their own within-subject means, and thus expresses the variability at the intra-individual level.

Due to the decomposition (1) and the covariance matrix meaning of $\boldsymbol{\Sigma}_{\mathrm{B}}$ and $\boldsymbol{\Sigma}_{\mathrm{W}}$, the BS and WS components in (2) can be described by two separate factor models, i.e., one applied to $\boldsymbol{\Sigma}_{\mathrm{B}}$ and the other to $\boldsymbol{\Sigma}_{\mathrm{W}}$ (Muthén, 1991, 1994). However, in a purely exploratory approach, factor analysis is usually carried out on correlation matrices instead of covariance matrices to account for the potentially not straight comparability of the original p variables X_j regarding magnitude, variability, and unit of measurement. In other words, instead of $\boldsymbol{\Sigma}_{\mathrm{T}}$, the starting point is the total correlation matrix $\mathbf{R}_{\mathrm{T}}$ of the

vector $\boldsymbol{X}$, which contains the "total" correlation coefficients $\rho\left(X_j, X_l\right)$ between the original variables, $j \neq l = 1,\dots,p$, not considering the two-level hierarchical data structure.

As known, $\mathbf{R}_\mathrm{T}$ can be expressed as the covariance matrix of the vector $\boldsymbol{Z}$ of standardized variables Z_j with values $z_{tij} = (x_{tij} - \mu_j)/\sigma_{\mathrm{T}j}$, where $\sigma_{\mathrm{T}j}$ is the total population standard deviation given as the square root of $\mathrm{Var}_\mathrm{T}(X_j)$, i.e., the j-th element on the diagonal of matrix $\mathbf{\Sigma}_\mathrm{T}$. Formally, let $\mathbf{D}_{\sigma_T}^{-1}$ be the $(p \times p)$ diagonal matrix with the reciprocals of $\sigma_{\mathrm{T}j}$:

$$\mathbf{D}_{\sigma_T}^{-1} = \mathrm{diag}\left\{\frac{1}{\sigma_{\mathrm{T}j}}\right\}_{j=1,\dots,p}.$$

Then: $\boldsymbol{Z} = \mathbf{D}_{\sigma_T}^{-1}(\boldsymbol{X} - \boldsymbol{\mu})$. By the properties of covariance matrices, it can be obtained:

$$\mathbf{R}_\mathrm{T} = \mathrm{V}(\boldsymbol{Z}) = \mathrm{V}[\mathbf{D}_{\sigma_T}^{-1}(\boldsymbol{X} - \boldsymbol{\mu})] = \mathbf{D}_{\sigma_T}^{-1}\,\mathrm{V}(\boldsymbol{X})\mathbf{D}_{\sigma_T}^{-1} = \mathbf{D}_{\sigma_T}^{-1}\mathbf{\Sigma}_\mathrm{T}\mathbf{D}_{\sigma_T}^{-1}. \tag{5}$$

Moreover, by using decomposition (1) in (5), it derives:

$$\mathbf{R}_\mathrm{T} = \mathbf{D}_{\sigma_T}^{-1}\mathbf{\Sigma}_\mathrm{T}\mathbf{D}_{\sigma_T}^{-1} = \mathbf{D}_{\sigma_T}^{-1}\left(\mathbf{\Sigma}_\mathrm{B} + \mathbf{\Sigma}_\mathrm{W}\right)\mathbf{D}_{\sigma_T}^{-1} = \mathbf{D}_{\sigma_T}^{-1}\mathbf{\Sigma}_\mathrm{B}\mathbf{D}_{\sigma_T}^{-1} + \mathbf{D}_{\sigma_T}^{-1}\mathbf{\Sigma}_\mathrm{W}\mathbf{D}_{\sigma_T}^{-1}. \tag{6}$$

Unlike the matrices $\mathbf{\Sigma}_\mathrm{B}$ and $\mathbf{\Sigma}_\mathrm{W}$ in (1), which preserve the meaning of covariance matrices, the two additive components $\mathbf{D}_{\sigma_T}^{-1}\mathbf{\Sigma}_\mathrm{B}\mathbf{D}_{\sigma_T}^{-1}$ and $\mathbf{D}_{\sigma_T}^{-1}\mathbf{\Sigma}_\mathrm{W}\mathbf{D}_{\sigma_T}^{-1}$ in (6) cannot be intended as correlation matrices because the elements in $\mathbf{\Sigma}_\mathrm{B}$ and $\mathbf{\Sigma}_\mathrm{W}$ are not divided by the right products of standard deviations. To have the correlation counterparts of $\mathbf{\Sigma}_\mathrm{B}$ and $\mathbf{\Sigma}_\mathrm{W}$, let $\mathbf{D}_{\sigma_B}^{-1}$ and $\mathbf{D}_{\sigma_W}^{-1}$ be two $(p \times p)$ diagonal matrices with the reciprocals of $\sigma_{\mathrm{B}j}$ and $\sigma_{\mathrm{W}j}$, respectively:

$$\mathbf{D}_{\sigma_B}^{-1} = \mathrm{diag}\left\{\frac{1}{\sigma_{\mathrm{B}j}}\right\}_{j=1,\dots,p} \qquad \text{and:} \qquad \mathbf{D}_{\sigma_W}^{-1} = \mathrm{diag}\left\{\frac{1}{\sigma_{\mathrm{W}j}}\right\}_{j=1,\dots,p},$$

where $\sigma_{\mathrm{B}j}$ is the square root of the BS variance $\mathrm{Var}_\mathrm{B}(X_j)$, the j-th element on the diagonal of $\mathbf{\Sigma}_\mathrm{B}$, and $\sigma_{\mathrm{W}j}$ is the square root of the WS variance, the j-th element on the diagonal of $\mathbf{\Sigma}_\mathrm{W}$. Then, two standardizations can be introduced similarly to the previous $\boldsymbol{Z} = \mathbf{D}_{\sigma_T}^{-1}(\boldsymbol{X} - \boldsymbol{\mu})$. The first standardization involves vector $\bar{\boldsymbol{X}}$ of the within-subject mean variables $\bar{X}_j$:

$$\bar{\boldsymbol{Z}} = \mathbf{D}_{\sigma_B}^{-1}(\bar{\boldsymbol{X}} - \boldsymbol{\mu}), \tag{7}$$

i.e., $\bar{\boldsymbol{Z}}$ contains the standardized within-subject means $\bar{Z}_j = \frac{\bar{X}_j - \mu_j}{\sigma_{\mathrm{B}j}}$ with values $\bar{z}_{.ij} = \frac{\bar{x}_{.ij} - \mu_j}{\sigma_{\mathrm{B}j}}$, for $i = 1, \ldots, n$ and $j = 1, \ldots, p$, for which it holds: $\mathrm{M}(\bar{\boldsymbol{Z}}) = \mathbf{0}$ and:

$$\mathrm{V}(\bar{\boldsymbol{Z}}) = \mathbf{D}_{\sigma_B}^{-1}\, \mathrm{V}(\bar{\boldsymbol{X}} - \boldsymbol{\mu})\mathbf{D}_{\sigma_B}^{-1} = \mathbf{D}_{\sigma_B}^{-1}\, \mathrm{V}(\bar{\boldsymbol{X}})\mathbf{D}_{\sigma_B}^{-1} = \mathbf{D}_{\sigma_B}^{-1}\boldsymbol{\Sigma}_{\mathrm{B}}\mathbf{D}_{\sigma_B}^{-1} = \mathbf{R}_{\mathrm{B}}\,. \tag{8}$$

In (8), $\mathbf{R}_{\mathrm{B}} = \mathrm{V}(\bar{\boldsymbol{Z}})$ is the BS correlation matrix; it contains the correlation coefficients $\rho(\bar{X}_j, \bar{X}_l)$ between the within-subject means or, equivalently, the correlation coefficients $\rho(\bar{Z}_j, \bar{Z}_l)$ between the standardized within-subject means, $j \neq l = 1, \ldots, p$.

The second standardization regards the n vectors $\boldsymbol{X}_i$ of the variables X_{ij}:

$$\tilde{\boldsymbol{Z}}_i = \mathbf{D}_{\sigma_W}^{-1}(\boldsymbol{X}_i - \bar{\mathbf{x}}_i)\,, \qquad i = 1, \ldots, n\,, \tag{9}$$

where each p-dimensional vector $\tilde{\boldsymbol{Z}}_i$ contains the standardized individual variables $\tilde{Z}_{ij} = \frac{X_{ij} - \bar{x}_{.ij}}{\sigma_{\mathrm{W}j}} = \frac{\tilde{X}_{ij}}{\sigma_{\mathrm{W}j}}$ with values $\tilde{z}_{tij} = \frac{x_{tij} - \bar{x}_{.ij}}{\sigma_{\mathrm{W}j}} = \frac{\tilde{x}_{tij}}{\sigma_{\mathrm{W}j}}$, for $t = 1, \ldots, T_i$ and $j = 1, \ldots, p$, for which it holds: $\mathrm{M}(\tilde{\boldsymbol{Z}}_i) = \mathbf{0}$ and:

$$\mathrm{V}(\tilde{\boldsymbol{Z}}_i) = \mathbf{D}_{\sigma_W}^{-1}\, \mathrm{V}(\boldsymbol{X}_i - \bar{\mathbf{x}}_i)\mathbf{D}_{\sigma_W}^{-1} = \mathbf{D}_{\sigma_W}^{-1}\, \mathrm{V}(\boldsymbol{X}_i)\mathbf{D}_{\sigma_W}^{-1} = \mathbf{D}_{\sigma_W}^{-1}\boldsymbol{\Sigma}_i\mathbf{D}_{\sigma_W}^{-1}\,, \tag{10}$$

for all $i = 1, \ldots, n$. From (10), the WS correlation matrix $\mathbf{R}_{\mathrm{W}}$ can then be obtained as the pooled average of $\mathrm{V}(\tilde{\boldsymbol{Z}}_i)$ over the n subjects:

$$\frac{1}{N}\sum_{i=1}^{n} \mathrm{V}(\tilde{\boldsymbol{Z}}_i)T_i = \mathbf{D}_{\sigma_W}^{-1}\left[\frac{1}{N}\sum_{i=1}^{n}\boldsymbol{\Sigma}_i T_i\right]\mathbf{D}_{\sigma_W}^{-1} = \mathbf{D}_{\sigma_W}^{-1}\boldsymbol{\Sigma}_{\mathrm{W}}\mathbf{D}_{\sigma_W}^{-1} = \mathbf{R}_{\mathrm{W}}\,. \tag{11}$$

In practice, the WS correlation matrix $\mathbf{R}_{\mathrm{W}}$ contains the correlation coefficients $\rho(\tilde{X}_j, \tilde{X}_l)$, $j \neq l = 1, \ldots, p$, between the within-subject-centered variables $\tilde{X}_j$, each of which assumes all the differences $\tilde{x}_{tij} = x_{tij} - \bar{x}_{.ij}$ for $t = 1, \ldots, T_i$ and $i = 1, \ldots, n$. Equivalently, $\mathbf{R}_{\mathrm{W}}$ contains the correlation coefficients $\rho(\tilde{Z}_j, \tilde{Z}_l)$, $j \neq l = 1, \ldots, p$, between the standardized individual variables $\tilde{Z}_j$, each of which assumes all the standardized individual values $\tilde{z}_{tij} = \frac{\tilde{x}_{tij}}{\sigma_{\mathrm{W}j}}$ for $t = 1, \ldots, T_i$ and $i = 1, \ldots, n$.

Now, using the two correlation matrices $\mathbf{R}_{\mathrm{B}}$ and $\mathbf{R}_{\mathrm{W}}$ the decomposition in (6) can be re-expressed as follows:

$$\begin{aligned}\mathbf{R}_{\mathrm{T}} &= \mathbf{D}_{\sigma_T}^{-1}\boldsymbol{\Sigma}_{\mathrm{T}}\mathbf{D}_{\sigma_T}^{-1} = \mathbf{D}_{\sigma_T}^{-1}\boldsymbol{\Sigma}_{\mathrm{B}}\mathbf{D}_{\sigma_T}^{-1} + \mathbf{D}_{\sigma_T}^{-1}\boldsymbol{\Sigma}_{\mathrm{W}}\mathbf{D}_{\sigma_T}^{-1} = \\ &= \mathbf{D}_{\sigma_T}^{-1}\mathbf{D}_{\sigma_B}\left(\mathbf{D}_{\sigma_B}^{-1}\boldsymbol{\Sigma}_{\mathrm{B}}\mathbf{D}_{\sigma_B}^{-1}\right)\mathbf{D}_{\sigma_B}\mathbf{D}_{\sigma_T}^{-1} + \mathbf{D}_{\sigma_T}^{-1}\mathbf{D}_{\sigma_W}\left(\mathbf{D}_{\sigma_W}^{-1}\boldsymbol{\Sigma}_{\mathrm{W}}\mathbf{D}_{\sigma_W}^{-1}\right)\mathbf{D}_{\sigma_W}\mathbf{D}_{\sigma_T}^{-1} = \\ &= \mathbf{D}_{\sigma_T}^{-1}\mathbf{D}_{\sigma_B}\mathbf{R}_{\mathrm{B}}\mathbf{D}_{\sigma_B}\mathbf{D}_{\sigma_T}^{-1} + \mathbf{D}_{\sigma_T}^{-1}\mathbf{D}_{\sigma_W}\mathbf{R}_{\mathrm{W}}\mathbf{D}_{\sigma_W}\mathbf{D}_{\sigma_T}^{-1}\ .\end{aligned} \tag{12}$$

By observing that:

$$\mathbf{D}_{\sigma_T}^{-1}\mathbf{D}_{\sigma_B} = \mathrm{diag}\left\{\frac{\sigma_{\mathrm{B}j}}{\sigma_{\mathrm{T}j}}\right\}_{j=1,\ldots,p} = \mathbf{D}_{\sigma_B/\sigma_T} \qquad \text{and:} \qquad \mathbf{D}_{\sigma_T}^{-1}\mathbf{D}_{\sigma_W} = \mathrm{diag}\left\{\frac{\sigma_{\mathrm{W}j}}{\sigma_{\mathrm{T}j}}\right\}_{j=1,\ldots,p} = \mathbf{D}_{\sigma_W/\sigma_T}\,,$$

the relation (12) can be finally written as:

$$\mathbf{R}_{\mathrm{T}} = \mathbf{D}_{\sigma_B/\sigma_T}\mathbf{R}_{\mathrm{B}}\mathbf{D}_{\sigma_B/\sigma_T} + \mathbf{D}_{\sigma_W/\sigma_T}\mathbf{R}_{\mathrm{W}}\mathbf{D}_{\sigma_W/\sigma_T}\ . \tag{13}$$

The decomposition (13) provides the theoretical justification, which seems to be lacking in the statistical literature, on which base also in the case of correlation matrices, two factor models can be applied separately, one to $\mathbf{R}_{\mathrm{B}}$ and the other to $\mathbf{R}_{\mathrm{W}}$. Specifically, concerning the BS component, the objective is to reproduce $\mathbf{R}_{\mathrm{B}}$ in (13) as best as possible by a small set of $q_B < p$ BS common factors according to the following BS factor model applied to the vector $\bar{\boldsymbol{Z}}$ in (7):

$$\bar{\boldsymbol{Z}} = \mathbf{A}_{\mathrm{B}}\boldsymbol{F}_{\mathrm{B}} + \boldsymbol{U}_{\mathrm{B}}\,, \tag{14}$$

where:

- $\mathbf{A}_{\mathrm{B}} = \left[a_{\mathrm{B}js}\right]_{\substack{j=1,\ldots,p\\ s=1,\ldots,q_B}}$ is the $(p\times q_B)$ BS factor loading matrix;
- $\boldsymbol{F}_{\mathrm{B}} = (F_{\mathrm{B}1},\ldots,F_{\mathrm{B}q_B})'$ is the vector containing $q_B < p$ BS common factors $F_{\mathrm{B}s}$, $s = 1,\ldots,q_B$, each having scores $f_{\mathrm{B}is}$ for $i = 1,\ldots,n$ (i.e., each BS factor assumes one score for each subject);
- $\boldsymbol{U}_{\mathrm{B}} = (U_{\mathrm{B}1},\ldots,U_{\mathrm{B}p})'$ is the vector of the p BS unique factors $U_{\mathrm{B}j}$, $j = 1,\ldots,p$, with values $u_{\mathrm{B}ij}$ for $i = 1,\ldots,n$.

Analogously, concerning the WS component, the objective is to reproduce $\mathbf{R}_{\mathrm{W}}$ in (13) as best as possible by a small set of $q_W < p$ WS common factors according to the following WS factor model applied to the p-dimensional vector $\tilde{\boldsymbol{Z}} = (\tilde{Z}_1,\ldots,\tilde{Z}_j,\ldots,\tilde{Z}_p)'$ of the standardized individual variables $\tilde{Z}_j$ previously described:

$$\tilde{\boldsymbol{Z}} = \mathbf{A}_{\mathrm{W}}\boldsymbol{F}_{\mathrm{W}} + \boldsymbol{U}_{\mathrm{W}}, \tag{15}$$

where:

- $\mathbf{A}_{\mathrm{W}} = \left[a_{\mathrm{W}js}\right]_{\substack{j=1,\ldots,p \\ s=1,\ldots,q_W}}$ is the $(p \times q_W)$ WS factor loading matrix;
- $\boldsymbol{F}_{\mathrm{W}} = (F_{\mathrm{W}1},\ldots,F_{\mathrm{W}q_W})'$ is the vector containing $q_W < p$ WS common factors $F_{\mathrm{W}s}$, $s=1,\ldots,q_W$, each having scores $f_{\mathrm{W}tis}$ for $t=1,\ldots,T_i$ and $i=1,\ldots,n$ (i.e., each WS factor assumes T_i scores for each subject);
- $\boldsymbol{U}_{\mathrm{W}} = (U_{\mathrm{W}1},\ldots,U_{\mathrm{W}p})'$ is the vector of the p WS unique factors $U_{\mathrm{W}j}$, $j=1,\ldots,p$, with values $u_{\mathrm{W}tij}$ for $t=1,\ldots,T_i$ and $i=1,\ldots,n$.

Standard assumptions concerning the factor models (14)-(15) are the following:

a) The BS and WS common factors are standardized so that $\mathrm{M}(\boldsymbol{F}_{\mathrm{B}}) = \mathbf{0}$ and $\mathrm{M}(\boldsymbol{F}_{\mathrm{W}}) = \mathbf{0}$, while the respective covariance matrices of $\boldsymbol{F}_{\mathrm{B}}$ and $\boldsymbol{F}_{\mathrm{W}}$:

   i) $\mathrm{V}(\boldsymbol{F}_{\mathrm{B}}) = \mathrm{M}(\boldsymbol{F}_{\mathrm{B}}\boldsymbol{F}_{\mathrm{B}}') = \boldsymbol{\Phi}_{\mathrm{B}}$
   ii) $\mathrm{V}(\boldsymbol{F}_{\mathrm{W}}) = \mathrm{M}(\boldsymbol{F}_{\mathrm{W}}\boldsymbol{F}_{\mathrm{W}}') = \boldsymbol{\Phi}_{\mathrm{W}}$

   are correlation matrices. Moreover, $\boldsymbol{F}_{\mathrm{B}}$ and $\boldsymbol{F}_{\mathrm{W}}$ are reciprocally uncorrelated so that:

   iii) $\mathrm{Cov}(\boldsymbol{F}_{\mathrm{B}},\boldsymbol{F}_{\mathrm{W}}) = \mathrm{M}(\boldsymbol{F}_{\mathrm{B}}\boldsymbol{F}_{\mathrm{W}}') = \mathbf{O}_{q_B \times q_W}$ or also: $\mathrm{Cov}(\boldsymbol{F}_{\mathrm{W}},\boldsymbol{F}_{\mathrm{B}}) = \mathrm{M}(\boldsymbol{F}_{\mathrm{W}}\boldsymbol{F}_{\mathrm{B}}') = \mathbf{O}_{q_W \times q_B}$,

   where $\mathbf{O}$ is the null matrix. Usually, the factor models (14)-(15) are simplified by introducing a further assumption on the BS and WS common factors, i.e., their uncorrelation. Accordingly, assumptions i)-ii) are re-expressed as:

   i') $\mathrm{V}(\boldsymbol{F}_{\mathrm{B}}) = \mathrm{M}(\boldsymbol{F}_{\mathrm{B}}\boldsymbol{F}_{\mathrm{B}}') = \mathbf{I}_{q_B}$
   ii') $\mathrm{V}(\boldsymbol{F}_{\mathrm{W}}) = \mathrm{M}(\boldsymbol{F}_{\mathrm{W}}\boldsymbol{F}_{\mathrm{W}}') = \mathbf{I}_{q_W}$,

   where $\mathbf{I}_{q_B}$ and $\mathbf{I}_{q_W}$ are two identity matrices of order q_B and q_W, respectively;

b) the unique factors are uncorrelated so that the covariance matrices of $\boldsymbol{U}_{\mathrm{B}}$ and $\boldsymbol{U}_{\mathrm{W}}$:

   i) $\mathrm{V}(\boldsymbol{U}_{\mathrm{B}}) = \mathrm{M}(\boldsymbol{U}_{\mathrm{B}}\boldsymbol{U}_{\mathrm{B}}') = \boldsymbol{\Gamma}_{\mathrm{B}} = \mathrm{diag}\{1-\gamma_{\mathrm{B}j}^2\}_{j=1,\ldots,p}$
   ii) $\mathrm{V}(\boldsymbol{U}_{\mathrm{W}}) = \mathrm{M}(\boldsymbol{U}_{\mathrm{W}}\boldsymbol{U}_{\mathrm{W}}') = \boldsymbol{\Gamma}_{\mathrm{W}} = \mathrm{diag}\{1-\gamma_{\mathrm{W}j}^2\}_{j=1,\ldots,p}$

   are diagonal matrices whose elements $1-\gamma_{\mathrm{B}j}^2$ and $1-\gamma_{\mathrm{W}j}^2$ are termed as "uniqueness." Moreover,

$\boldsymbol{U}_{\mathrm{B}}$ and $\boldsymbol{U}_{\mathrm{W}}$ are reciprocally uncorrelated so that:

iii) $\mathrm{Cov}(\boldsymbol{U}_{\mathrm{B}}, \boldsymbol{U}_{\mathrm{W}}) = \mathrm{M}(\boldsymbol{U}_{\mathrm{B}}\boldsymbol{U}'_{\mathrm{W}}) = \mathbf{O}_{q_B \times q_W}$ or also: $\mathrm{Cov}(\boldsymbol{U}_{\mathrm{W}}, \boldsymbol{U}_{\mathrm{B}}) = \mathrm{M}(\boldsymbol{U}_{\mathrm{W}}\boldsymbol{U}'_{\mathrm{B}}) = \mathbf{O}_{q_W \times q_B}$;

c) all the common factors and unique factors are uncorrelated:

i) $\mathrm{Cov}(\boldsymbol{F}_{\mathrm{B}}, \boldsymbol{U}_{\mathrm{B}}) = \mathrm{M}(\boldsymbol{F}_{\mathrm{B}}\boldsymbol{U}'_{\mathrm{B}}) = \mathbf{O}_{q_B \times p}$ or also: $\mathrm{Cov}(\boldsymbol{U}_{\mathrm{B}}, \boldsymbol{F}_{\mathrm{B}}) = \mathrm{M}(\boldsymbol{U}_{\mathrm{B}}\boldsymbol{F}'_{\mathrm{B}}) = \mathbf{O}_{p \times q_B}$

ii) $\mathrm{Cov}(\boldsymbol{F}_{\mathrm{W}}, \boldsymbol{U}_{\mathrm{W}}) = \mathrm{M}(\boldsymbol{F}_{\mathrm{W}}\boldsymbol{U}'_{\mathrm{W}}) = \mathbf{O}_{q_W \times p}$ or also: $\mathrm{Cov}(\boldsymbol{U}_{\mathrm{W}}, \boldsymbol{F}_{\mathrm{W}}) = \mathrm{M}(\boldsymbol{U}_{\mathrm{W}}\boldsymbol{F}'_{\mathrm{W}}) = \mathbf{O}_{p \times q_W}$

iii) $\mathrm{Cov}(\boldsymbol{F}_{\mathrm{B}}, \boldsymbol{U}_{\mathrm{W}}) = \mathrm{M}(\boldsymbol{F}_{\mathrm{B}}\boldsymbol{U}'_{\mathrm{W}}) = \mathbf{O}_{q_B \times p}$ or also: $\mathrm{Cov}(\boldsymbol{U}_{\mathrm{W}}, \boldsymbol{F}_{\mathrm{B}}) = \mathrm{M}(\boldsymbol{U}_{\mathrm{W}}\boldsymbol{F}'_{\mathrm{B}}) = \mathbf{O}_{p \times q_B}$

iv) $\mathrm{Cov}(\boldsymbol{F}_{\mathrm{W}}, \boldsymbol{U}_{\mathrm{B}}) = \mathrm{M}(\boldsymbol{F}_{\mathrm{W}}\boldsymbol{U}'_{\mathrm{B}}) = \mathbf{O}_{q_W \times p}$ or also: $\mathrm{Cov}(\boldsymbol{U}_{\mathrm{B}}, \boldsymbol{F}_{\mathrm{W}}) = \mathrm{M}(\boldsymbol{U}_{\mathrm{B}}\boldsymbol{F}'_{\mathrm{W}}) = \mathbf{O}_{p \times q_W}$.

Based on the factor models (14)-(15) and the relative assumptions, the two BS and WS correlation matrices $\mathbf{R}_{\mathrm{B}}$ and $\mathbf{R}_{\mathrm{W}}$ admit each a decomposition into two additive terms, the one depending on the common factors, the other on the unique factors. Specifically, using (14), we obtain for $\mathbf{R}_{\mathrm{B}}$:

$$\begin{aligned} \mathbf{R}_{\mathrm{B}} &= \mathrm{V}(\bar{\boldsymbol{Z}}) = \mathbf{A}_{\mathrm{B}}\,\mathrm{V}(\boldsymbol{F}_{\mathrm{B}})\mathbf{A}'_{\mathrm{B}} + \mathrm{V}(\boldsymbol{U}_{\mathrm{B}}) = \mathbf{A}_{\mathrm{B}}\boldsymbol{\Phi}_{\mathrm{B}}\mathbf{A}'_{\mathrm{B}} + \boldsymbol{\Gamma}_{\mathrm{B}} = \tilde{\mathbf{R}}_{\mathrm{B}} + \boldsymbol{\Gamma}_{\mathrm{B}} \\ &\underset{\substack{\uparrow \\ \text{assumption (ai')}}}{=} \mathbf{A}_{\mathrm{B}}\mathbf{A}'_{\mathrm{B}} + \boldsymbol{\Gamma}_{\mathrm{B}} = \tilde{\mathbf{R}}_{\mathrm{B}} + \boldsymbol{\Gamma}_{\mathrm{B}} \end{aligned} \tag{16}$$

where in (16), $\tilde{\mathbf{R}}_{\mathrm{B}} = \mathbf{A}_{\mathrm{B}}\boldsymbol{\Phi}_{\mathrm{B}}\mathbf{A}'_{\mathrm{B}}$ or $\tilde{\mathbf{R}}_{\mathrm{B}} = \mathbf{A}_{\mathrm{B}}\mathbf{A}'_{\mathrm{B}}$ is the so-called BS reduced correlation matrix whose elements on the diagonal are the BS communalities $\gamma^2_{\mathrm{B}j}$:

$$\gamma^2_{\mathrm{B}j} = \mathrm{Var}\left(\sum_{s=1}^{q_B} a_{\mathrm{B}js}F_{\mathrm{B}s}\right), \qquad j = 1, \dots, p \tag{17}$$

Each of such quantities in (17) provides the proportion of the variance $\mathrm{Var}(\bar{Z}_j) = 1$ explained by the q_B BS common factors $\boldsymbol{F}_{\mathrm{B}}$, while the uniqueness $1 - \gamma^2_{\mathrm{B}j} = \mathrm{Var}(U_{\mathrm{B}j})$ represents the proportion of $\mathrm{Var}(\bar{Z}_j)$ that cannot be reproduced by $\boldsymbol{F}_{\mathrm{B}}$. The total BS communality γ^2_{B} is the sum of $\gamma^2_{\mathrm{B}j}$ over the p variables:

$$\gamma^2_{\mathrm{B}} = \sum_{j=1}^{p} \gamma^2_{\mathrm{B}j}\,. \tag{18}$$

Considering that the total BS variance is given by: $V_{\mathrm{B}} = \mathrm{tr}(\mathbf{R}_{\mathrm{B}}) = p$, the ratio $\gamma^2_{\mathrm{B}} / p$ provides the proportion of the total BS variance reproduced by the q_B BS common factors.

Under the further assumption (ai′) of uncorrelation between the BS common factors, the communalities (17) can be re-expressed as:

$$\gamma^2_{\mathrm{B}j} = \mathrm{Var}\left(\sum_{s=1}^{q_B} a_{\mathrm{B}js} F_{\mathrm{B}s}\right) = \sum_{s=1}^{q_B} a^2_{\mathrm{B}js}\,\mathrm{Var}(F_{\mathrm{B}s}) = \sum_{s=1}^{q_B} a^2_{\mathrm{B}js}\,, \qquad j = 1,\dots,p\,. \tag{19}$$

Accordingly, the single $a^2_{\mathrm{B}js}$ can be directly interpreted as the proportion of the variance $\mathrm{Var}(\bar{Z}_j)$ reproduced by the s-th BS common factor $F_{\mathrm{B}s}$, $s = 1,\dots,q_B$. Moreover, the proportion of the total BS variance reproduced by the s-th BS common factor $F_{\mathrm{B}s}$ is given by:

$$\frac{V_{\mathrm{B}s}}{V_{\mathrm{B}}} = \frac{\sum_{j=1}^{p} a^2_{\mathrm{B}js}}{p}, \qquad s = 1,\dots,q_B\,. \tag{20}$$

As far as concerns the WS counterpart, similar results hold. From factor model (15), we obtain for $\mathbf{R}_{\mathrm{W}}$:

$$\begin{aligned} \mathbf{R}_{\mathrm{W}} &= \mathrm{V}(\tilde{\boldsymbol{Z}}) = \mathbf{A}_{\mathrm{W}}\,\mathrm{V}(\boldsymbol{F}_{\mathrm{W}})\mathbf{A}'_{\mathrm{W}} + \mathrm{V}(\boldsymbol{U}_{\mathrm{W}}) = \mathbf{A}_{\mathrm{W}}\boldsymbol{\Phi}_{\mathrm{W}}\mathbf{A}'_{\mathrm{W}} + \boldsymbol{\Gamma}_{\mathrm{W}} = \tilde{\mathbf{R}}_{\mathrm{W}} + \boldsymbol{\Gamma}_{\mathrm{W}} \\ &\underset{\substack{\uparrow \\ \text{assumption (aii}')}}{=} \mathbf{A}_{\mathrm{W}}\mathbf{A}'_{\mathrm{W}} + \boldsymbol{\Gamma}_{\mathrm{W}} = \tilde{\mathbf{R}}_{\mathrm{W}} + \boldsymbol{\Gamma}_{\mathrm{W}} \end{aligned} \tag{21}$$

where in (21), $\tilde{\mathbf{R}}_{\mathrm{W}} = \mathbf{A}_{\mathrm{W}}\boldsymbol{\Phi}_{\mathrm{W}}\mathbf{A}'_{\mathrm{W}}$ or $\tilde{\mathbf{R}}_{\mathrm{W}} = \mathbf{A}_{\mathrm{W}}\mathbf{A}'_{\mathrm{W}}$ is the WS reduced correlation matrix, which contains on the diagonal the WS communalities $\gamma^2_{\mathrm{W}j}$:

$$\gamma^2_{\mathrm{W}j} = \mathrm{Var}\left(\sum_{s=1}^{q_W} a_{\mathrm{W}js} F_{\mathrm{W}s}\right), \qquad j = 1,\dots,p\,. \tag{22}$$

Then, similarly to the BS communalities, $\gamma^2_{\mathrm{W}j}$ in (22) gives the proportion of the variance $\mathrm{Var}(\tilde{Z}_j) = 1$ explained by the q_W WS common factors $\boldsymbol{F}_{\mathrm{W}}$, while the uniqueness $1 - \gamma^2_{\mathrm{W}j} = \mathrm{Var}(U_{\mathrm{W}j})$ is the proportion of $\mathrm{Var}(\tilde{Z}_j)$ that cannot be reproduced by $\boldsymbol{F}_{\mathrm{W}}$. The total WS communality γ^2_{W} is given by:

$$\gamma^2_{\mathrm{W}} = \sum_{j=1}^{p} \gamma^2_{\mathrm{W}j}\,, \tag{23}$$

while, given that $V_{\mathrm{W}} = \mathrm{tr}(\mathbf{R}_{\mathrm{W}}) = p$ is the total WS variance, the ratio $\gamma^2_{\mathrm{W}} / p$ is the proportion of the total WS variance reproduced by the q_{W} WS common factors.

Under the further assumption (aii′) of uncorrelation between the WS common factors, the communalities (22) can be written as:

$$\gamma^2_{\mathrm{W}j} = \mathrm{Var}\left(\sum_{s=1}^{q_W} a_{\mathrm{W}js} F_{\mathrm{W}s}\right) = \sum_{s=1}^{q_W} a^2_{\mathrm{W}js}\,, \qquad j = 1,\dots,p\,. \tag{24}$$

So, the single $a_{\mathrm{W}js}^2$ is the proportion of the variance $\mathrm{Var}(\tilde{Z}_j)$ reproduced by the s-th WS common factor $F_{\mathrm{W}s}$, $s=1,\ldots,q_W$, while the proportion of the total WS variance reproduced by the s-th WS common factor $F_{\mathrm{W}s}$ is given by:

$$\frac{V_{\mathrm{W}s}}{V_{\mathrm{W}}}=\frac{\sum_{j=1}^{p}a_{\mathrm{W}js}^2}{p}, \qquad s=1,\ldots,q_W. \tag{25}$$

As a final consideration, similarly to the standard one-level EFA, the primary step in MEFA is computing the two factor loading matrices $\mathbf{A}_{\mathrm{B}}$ in (14) and $\mathbf{A}_{\mathrm{W}}$ in (15), whose elements, under the assumptions (ai′) and (aii′), can be straight interpreted as correlation coefficients between variables and BS and WS common factors, respectively. That also requires choosing the ideal numbers $q_B < p$ and $q_W < p$ of BS and WS common factors, and then determining the factor scores $f_{\mathrm{B}is}$ and $f_{\mathrm{W}tis}$. Given that two separate factor models are *de facto* applied to the BS and WS correlation matrices $\mathbf{R}_{\mathrm{B}}$ and $\mathbf{R}_{\mathrm{W}}$, standard methods of EFA (Finch, 2020) can be applied to derive all the quantities of interest.

1.2 References

Finch, W. (2020). Exploratory Factor Analysis. Thousand Oaks, CA: SAGE Publications, Inc. doi: 10.4135/9781544339900

Härnqvist, K. (1978). Primary mental abilities at collective and individual level. J. Educ. Psychol. 70, 706–716.

Hox, J. J. (1993). “Factor analysis of multilevel data: Gauging the Muthén model,” in: Advances in Longitudinal and Multivariate Analysis in the Behavioral Sciences, eds. Oud, J. H. L, and van Blokland-Vogelesang, R. A. W. (Nijmegen, NL: ITS), 141–156

Muthén, B. O. (1991). Multilevel factor analysis of class and student achievement components. J. Educ. Meas. 28, 338–354. doi: 10.1111/j.1745-3984.1991.tb00363.x

Muthén, B. O. (1994). Multilevel covariance structure analysis. Sociol. Method. Res. 22, 376–398. doi:10.1177/0049124194022003006

Reise, S. P., Ventura, J., Nuechterlein, K. H., and Kim K. H. (2005). An illustration of multilevel factor analysis. J. Pers. Assess. 84, 126–136. doi: 10.1207/s15327752jpa8402_02

2 Supplementary Figures and Tables

2.1 Supplementary Tables

Supplementary Table 1. Descriptive statistics (median and MAD) of the study ANS proxies within the total athlete set and the cyclist and shooter groups computed over all the epochs.

	Cyclist group		***Shooter group***		***Total athlete set***	
**Variables**	**Median**	**MAD**	**Median**	**MAD**	**Median**	**MAD**
HR	106.49	32.00	118.22	27.28	112.40	29.67
RR RMS	39.25	21.82	25.34	12.62	29.55	16.90
RR TP	358.20	354.33	159.52	152.40	223.55	217.24
RR LFa	140.73	140.73	67.80	66.39	95.87	95.68
RR HFa	14.85	14.83	6.24	6.24	8.05	8.05
RMSSD	3.27	2.61	2.16	1.44	2.51	1.95
AC	–2.74	2.21	–2.20	1.63	–2.55	2.00
DC	2.86	2.35	2.28	1.66	2.55	2.00
RR LFnu	62.13	27.41	76.65	15.46	69.80	20.81
RR HFnu	13.29	10.28	13.48	9.29	13.38	9.61
RR Ro	0.53	0.19	0.55	0.16	0.54	0.18
P0v	51.69	20.97	52.33	21.30	52.01	21.04

Supplementary Table 2. Descriptive statistics (median and MAD) of the study ANS proxies within the total athlete set computed at each epoch.

	**Epoch 1**		**Epoch 2**		**Epoch 3**		**Epoch 4**		**Epoch 5**	
Variables	**Median**	**MAD**	**Median**	**MAD**	**Median**	**MAD**	**Median**	**MAD**	**Median**	**MAD**
HR	60.16	7.83	78.03	11.61	84.72	6.81	100.20	7.07	137.12	9.31
RR RMS	55.08	15.25	47.48	14.41	49.77	15.07	29.72	9.26	19.26	4.74
RR TP	2833.50	1376.66	2067.62	1241.26	918.38	511.62	296.98	140.41	50.47	22.58
RR LFa	639.54	349.33	1058.26	509.44	721.78	392.39	113.16	96.90	4.54	4.47
RR HFa	833.15	703.88	168.38	154.04	65.69	52.43	6.56	6.56	2.53	1.71
RMSSD	10.63	3.44	8.24	2.21	5.80	1.35	2.57	0.37	1.15	0.29
AC	–8.54	2.90	–10.09	1.78	–6.32	1.95	–3.33	0.87	–1.40	0.26
DC	6.53	1.99	9.02	1.94	4.98	2.07	2.48	0.84	0.96	0.36
RR LFnu	38.86	14.97	89.11	7.66	86.47	5.17	81.87	7.95	45.08	33.20
RR HFnu	57.58	16.31	8.05	5.38	9.21	5.50	8.08	8.08	19.24	15.49
RR Ro	0.26	0.06	0.44	0.08	0.48	0.09	0.58	0.06	0.75	0.06
P0v	13.52	7.35	38.94	6.46	48.68	14.04	57.76	9.13	77.72	10.34

	**Epoch 6**		**Epoch 7**		**Epoch 8**		**Epoch 9**	
Variables	**Median**	**MAD**	**Median**	**MAD**	**Median**	**MAD**	**Median**	**MAD**
HR	155.01	7.63	165.77	5.75	138.79	7.80	105.60	10.83
RR RMS	11.53	2.33	2.15	0.67	39.02	12.51	39.54	14.01
RR TP	13.06	8.03	3.88	1.89	103.84	68.07	365.16	227.30
RR LFa	0.54	0.54	1.48	1.48	25.16	24.68	164.58	103.42
RR HFa	0.77	0.44	0.40	0.24	3.51	2.76	34.25	27.21
RMSSD	0.59	0.12	0.40	0.04	1.73	0.74	3.38	1.53
AC	–0.66	0.14	–0.50	0.12	–0.58	0.51	–3.11	1.51
DC	0.47	0.20	0.41	0.08	1.85	0.78	3.94	1.39
RR LFnu	12.16	12.16	37.47	28.88	69.34	16.83	75.11	13.11
RR HFnu	18.12	10.19	9.64	4.75	15.31	9.99	16.93	9.02
RR Ro	0.72	0.07	0.12	0.06	0.76	0.04	0.52	0.05
P0v	66.40	9.19	8.04	4.69	80.62	5.21	50.44	11.63

Supplementary Table 3. Descriptive statistics (median and MAD) of the study ANS proxies within the cyclist group computed at each epoch.

	Epoch 1		Epoch 2		Epoch 3		Epoch 4		Epoch 5	
Variables	**Median**	**MAD**	**Median**	**MAD**	**Median**	**MAD**	**Median**	**MAD**	**Median**	**MAD**
HR	54.05	5.48	71.28	7.34	82.67	4.88	97.95	2.86	135.49	9.44
RR RMS	60.35	7.84	61.92	15.26	56.72	17.33	33.38	5.87	19.08	4.82
RR TP	3565.09	988.85	3696.63	1732.59	1270.83	501.06	382.2	129.54	46.81	24.23
RR LFa	972.83	568.67	1267.57	706.04	858.59	302.90	119.21	100.53	2.92	2.78
RR HFa	1461.69	671.90	344.87	191.92	79.15	79.15	0.00	0.00	2.45	1.79
RMSSD	13.87	3.27	9.48	1.95	5.96	0.76	2.77	0.29	1.17	0.30
AC	–8.1	3.21	–10.32	1.82	–7.29	1.65	-3.97	1.2	–1.25	0.32
DC	6.28	2.15	10.45	0.86	5.04	1.68	2.36	0.68	0.82	0.45
RR LFnu	30.82	7.36	80.69	13.89	89.56	3.43	81.15	6.55	42.26	38.66
RR HFnu	67.92	6.10	16.86	10.66	8.01	4.95	0.00	0.00	20.24	16.61
RR Ro	0.23	0.04	0.4	0.07	0.46	0.07	0.58	0.06	0.74	0.08
P0v	8.93	3.90	34.78	11.66	49.31	14.87	57.89	9.72	75.43	10.84

	Epoch 6		Epoch 7		Epoch 8		Epoch 9	
Variables	**Median**	**MAD**	**Median**	**MAD**	**Median**	**MAD**	**Median**	**MAD**
HR	153.78	6.17	165.76	4.05	135.95	7.18	97.58	8.91
RR RMS	11.9	1.79	2.04	0.44	59.56	13.50	48.88	6.44
RR TP	14.17	8.97	3.6	1.01	134.45	61.08	860.15	524.08
RR LFa	0.07	0.07	0.26	0.26	83.98	65.06	437.70	290.96
RR HFa	0.43	0.33	0.19	0.13	7.54	7.04	68.87	46.14
RMSSD	0.53	0.13	0.36	0.05	2.26	0.78	5.03	1.47
AC	–0.63	0.12	–0.47	0.09	–0.51	0.51	–4.43	1.76
DC	0.39	0.14	0.36	0.13	2.61	0.80	5.25	1.34
RR LFnu	4.68	4.68	15.73	15.73	65.74	18.21	72.04	11.04
RR HFnu	21.64	13.96	7.21	4.05	12.58	6.51	17.53	9.87
RR Ro	0.74	0.07	0.11	0.06	0.74	0.06	0.51	0.04
P0v	67.04	5.35	4.04	2.33	78.57	6.82	42.17	10.62

Supplementary Table 4. Descriptive statistics (median and MAD) of the study ANS proxies within the shooter group computed at each epoch.

	Epoch 1		Epoch 2		Epoch 3		Epoch 4		Epoch 5	
Variables	**Median**	**MAD**	**Median**	**MAD**	**Median**	**MAD**	**Median**	**MAD**	**Median**	**MAD**
HR	69.63	5.65	86.87	10.70	90.40	12.10	101.76	13.57	137.66	8.20
RR RMS	39.55	11.15	43.73	4.70	45.62	16.74	24.75	8.28	20.21	5.80
RR TP	1559.99	840.47	1776.76	524.99	737.63	486.45	188.50	128.36	51.02	24.75
RR LFa	412.04	173.13	751.20	529.38	558.56	430.27	107.11	85.34	13.25	11.80
RR HFa	175.55	152.24	54.78	40.87	34.18	34.18	16.87	16.87	2.83	1.66
RMSSD	8.21	2.40	7.31	1.99	5.29	2.41	2.36	0.63	1.12	0.11
AC	–8.58	2.32	–9.36	2.47	–5.18	2.12	–3.22	1.02	–1.43	0.30
DC	7.03	2.08	7.91	2.21	4.86	2.97	2.48	1.03	1.21	0.41
RR LFnu	55.64	21.68	94.66	3.16	85.05	7.35	82.59	12.11	64.65	29.19
RR HFnu	23.56	19.44	4.44	1.56	11.13	7.18	9.05	9.05	18.23	14.98
RR Ro	0.27	0.07	0.46	0.05	0.49	0.08	0.58	0.06	0.75	0.04
P0v	20.41	8.09	40.84	3.60	48.08	13.32	56.18	9.81	80.00	7.40

	Epoch 6		Epoch 7		Epoch 8		Epoch 9	
Variables	**Median**	**MAD**	**Median**	**MAD**	**Median**	**MAD**	**Median**	**MAD**
HR	157.97	10.99	165.78	8.14	144.90	9.78	116.07	9.32
RR RMS	11.46	3.55	2.89	0.95	34.23	4.70	23.59	5.54
RR TP	10.04	5.63	5.86	2.75	35.57	11.85	215.39	130.34
RR LFa	2.88	2.70	3.36	1.82	13.74	13.05	90.16	32.67
RR HFa	0.89	0.50	0.59	0.21	2.87	2.14	19.15	15.88
RMSSD	0.66	0.10	0.41	0.03	1.10	0.15	2.12	0.52
AC	–0.75	0.20	–0.53	0.11	–0.67	0.53	–1.79	0.73
DC	0.51	0.25	0.42	0.09	1.30	0.27	3.07	1.03
RR LFnu	62.94	25.47	65.88	11.88	72.93	18.93	82.17	11.51
RR HFnu	14.58	8.33	13.12	4.18	17.84	12.28	11.26	7.99
RR Ro	0.68	0.08	0.16	0.06	0.78	0.04	0.56	0.08
P0v	65.46	10.69	12.00	4.73	82.46	5.13	53.19	12.65

Supplementary Table 5. Descriptive statistics (median and MAD) of the study ANS proxies within the total athlete set and the cyclist and shooter groups considered at rest (epoch 1) and in the following comparisons (along with non-parametric tests): stand – rest (ΔEp2-Ep1), peak – rest (ΔEp7-Ep1), recovery last phase – peak (ΔEp9-Ep7).

	**Epoch 1**		**ΔEp2-Ep1**			**ΔEp7-Ep1**			**ΔEp9-Ep7**		
Total	**Med**	**MAD**	**Med**	**MAD**	**Sig.**	**Med**	**MAD**	**Sig.**	**Med**	**MAD**	**Sig.**
HR	60.16	7.83	16.55	6.37	***,†††	105.26	8.67	***,†††	–59.56	10.17	***,†††
RR RMS	55.08	15.25	–0.34	15.92		–53.78	14.53	***,†††	36.42	13.44	***,†††
RR TP	2833.50	1376.66	–180.78	1450.70		–2831.08	1376.26	***,†††	362.56	228.50	***,†††
RR LFa	639.54	349.33	272.68	595.28	*,†	–638.67	349.60	***,†††	162.67	106.87	***,†††
RR HFa	833.15	703.88	–403.30	403.43	***,†††	–832.80	703.99	***,†††	33.77	27.23	***,†††
RMSSD	10.63	3.44	–2.18	2.30	**,††	–10.22	3.49	***,†††	3.03	1.53	***,†††
AC	–8.54	2.90	–0.31	3.54		8.07	2.74	***,†††	–2.65	1.66	***,†††
DC	6.53	1.99	0.98	3.40		–6.19	2.14	***,†††	3.41	1.44	***,†††
RR LFnu	38.86	14.97	35.20	19.96	***,†††	–13.70	20.83		34.67	26.70	***,†††
RR HFnu	57.58	16.31	–32.76	19.24	***,†††	–42.17	19.80	***,†††	3.70	9.01	
RR Ro	0.26	0.06	0.17	0.06	***,†††	–0.11	0.10	***,††	0.41	0.08	***,†††
P0v	13.52	7.35	23.03	7.32	***,†††	–4.50	9.22	*,†	39.29	10.02	***,†††
Cyclists	**Med**	**MAD**	**Med**	**MAD**	**Sig.**	**Med**	**MAD**	**Sig.**	**Med**	**MAD**	**Sig.**
HR	54.05	5.48	17.56	7.14	***,†††	109.90	8.15	***,†††	–72.23	5.04	***,†††
RR RMS	60.35	7.84	7.11	19.52		–58.26	7.48	***,†††	47.26	6.62	***,†††
RR TP	3565.09	988.85	154.14	3251.85		–3552.18	976.92	***,†††	856.55	522.72	***,†††
RR LFa	972.83	568.67	289.59	572.82		–972.37	569.75	***,†††	437.70	291.22	***,†††
RR HFa	1461.69	671.90	–846.41	487.12	**,††	–1459.89	670.15	***,†††	68.74	46.13	***,†††
RMSSD	13.87	3.27	–4.10	3.16	*,††	–13.56	3.28	***,†††	4.62	1.43	***,†††
AC	–8.10	3.21	–1.01	4.46		7.13	2.91	***,†††	–3.90	1.65	***,†††
DC	6.28	2.15	1.19	5.50		–5.83	2.20	***,†††	4.77	1.57	***,†††
RR LFnu	30.82	7.36	38.74	21.41	***,†††	–26.19	18.68		57.96	14.77	***,†††
RR HFnu	67.92	6.10	–41.65	19.75	***,†††	–57.31	6.16	***,†††	5.76	12.08	†
RR Ro	0.23	0.04	0.17	0.12	**,†††	–0.10	0.09	**,††	0.42	0.03	***,†††
P0v	8.93	3.90	26.98	6.16	***,†††	–3.45	6.61		37.57	8.52	***,†††
Shooters	**Med**	**MAD**	**Med**	**MAD**	**Sig.**	**Med**	**MAD**	**Sig.**	**Med**	**MAD**	**Sig.**
HR	69.63	5.65	13.84	6.04	***,†††	94.87	10.25	***,†††	–50.86	4.96	***,†††
RR RMS	39.55	11.15	–1.87	7.27		–38.02	13.99	***,†††	20.71	6.50	***,†††
RR TP	1559.99	840.47	–235.54	995.56		–1557.66	842.83	***,†††	212.30	139.01	***,†††
RR LFa	412.04	173.13	255.78	621.60		–406.23	178.75	***,†††	85.82	33.43	***,†††
RR HFa	175.55	152.24	–116.92	126.57	**,††	–174.97	152.43	***,†††	18.26	16.26	***,†††
RMSSD	8.21	2.40	–0.56	1.82		–7.80	2.43	***,†††	1.75	0.76	***,†††
AC	–8.58	2.32	–0.18	2.85		8.20	2.35	***,†††	–1.50	1.10	**,††
DC	7.03	2.08	0.78	2.27		–6.57	2.26	***,†††	2.64	1.16	**,†††
RR LFnu	55.64	21.68	18.97	16.31	***,††	–8.10	16.11		21.91	17.60	**,††
RR HFnu	23.56	19.44	–16.19	15.50	***,††	–13.33	24.34	*,†	1.82	7.05	
RR Ro	0.27	0.07	0.18	0.05	***,††	–0.14	0.12	*	0.40	0.09	***,†††
P0v	20.41	8.09	19.34	6.15	***,†††	–11.13	10.70		39.68	14.30	***,†††

Legend.
Columns labeled with "ΔEpt-Epv" intend that (paired) differences between values at epochs t and v were first computed for each proxy X (i.e., $D = X_t - X_v$, with $t > v$, $t, v = 1, \dots, T = 9$) and then summarized through the median and MAD (Median Absolute Deviation).
Columns labeled with "Sig." report the results of the Wilcoxon signed-rank (WSR) test and the sign test for the paired differences considered in "ΔEpt-Epv." Let θ be the median of variable D, regarded as epoch effect. By those tests, the null hypothesis of zero epoch effect on the distribution of variable D is tested against the presence of epoch effect, i.e., $H_0\colon \theta = 0$ vs. $H_1\colon \theta \neq 0$. Significance level codes:

- Wilcoxon signed-rank test: *significant at 0.05 level, **significant at 0.01 level, ***significant at 0.001 level
- Sign test: †significant at 0.05 level, ††significant at 0.01 level, †††significant at 0.001 level.

Note: In its first column, Supplementary Table 5 contains the median and MAD of the 12 ANS proxies "at rest" (epoch 1) computed for the whole athlete set and within cyclist and shooter groups. Supplementary Table 5 also includes several pairwise comparisons of interest (i.e., stand-rest, peak-rest, and recovery last phase-peak) tested through the WSR and the sign tests. Numerous comparisons prove significant, among which several are strongly significant (i.e., both *p*-values are < 0.001). Moreover, specific trends appear by looking at the signs of the differences. For instance, HR median values at epochs 2 ("stand") and 7 ("peak") are clearly higher than epoch 1 (positive differences). In contrast, the HR median value is smaller at epoch 9 ("recovery last phase") compared to epoch 7 (negative difference). Again, median values of proxies such as RR RMS, RR TP, RR LFa, RR HFa, RMSSD, DC, RR HFnu, RR Ro, and P0v tend to decrease from epoch 1 to 7 while AC increases. The opposite schema is instead observed from epoch 7 to epoch 9. All that indicates there exist trend patterns that are worth examining further.

Supplementary Table 6. Cyclists' and shooters' individual heart rates (HR) at each epoch expressed as percentages of their maximal heart rate recorded at rest and stand and during the bicycle stress test.

Cyclists									
**ID**	**HR.1%**	**HR.2%**	**HR.3%**	**HR.4%**	**HR.5%**	**HR.6%**	**HR.7%**	**HR.8%**	**HR.9%**
1001	*33.91*	40.20	48.36	56.30	81.56	**92.00**	100	82.52	43.64
1002	*33.04*	38.03	50.96	59.58	82.78	**91.78**	100	80.22	59.18
1003	*38.98*	54.62	52.35	63.84	79.55	**91.42**	100	89.24	64.20
1004	*29.10*	40.80	48.95	59.54	78.17	**91.63**	100	75.96	45.21
1005	*32.61*	55.25	46.93	52.82	80.00	**91.96**	100	69.00	51.07
1006	*32.23*	38.26	56.98	70.59	87.54	**93.05**	100	82.31	46.84
1007	*26.54*	46.22	50.11	59.30	75.48	**95.33**	100	81.33	61.88
1008	*32.49*	37.47	44.50	59.44	85.14	**94.59**	100	79.17	58.04
1009	*36.97*	47.02	57.19	66.28	74.09	**92.44**	100	84.54	63.68
1010	*22.44*	27.60	54.65	67.25	79.79	**95.50**	100	80.35	57.46
1011	*34.03*	42.51	47.06	57.38	83.73	**96.32**	100	89.30	67.58
1012	*33.14*	43.97	53.44	59.10	74.68	**89.11**	100	83.10	56.84
1013	*29.92*	41.25	42.64	56.72	86.75	**97.07**	100	77.08	57.65
1014	*37.40*	55.41	54.80	60.27	85.23	**94.40**	100	69.04	57.23
1015	*32.37*	43.81	49.82	58.92	81.89	**94.21**	100	83.23	59.24
**Mean**	*32.34*	43.49	50.58	60.49	81.09	**93.93**	100	80.43	56.65
**sd**	4.19	7.53	4.35	4.64	4.25	2.18	0	5.92	7.06
**Median**	*32.61*	42.51	50.11	59.44	81.56	**93.05**	100	81.33	57.65
**MAD**	1.42	4.25	3.18	2.06	3.39	1.42	0	2.16	4.23
**Shooters**									
**ID**	**HR.1%**	**HR.2%**	**HR.3%**	**HR.4%**	**HR.5%**	**HR.6%**	**HR.7%**	**HR.8%**	**HR.9%**
2001	*39.56*	52.74	58.94	66.31	80.39	**93.00**	100	84.14	72.76
2002	*38.29*	52.95	60.71	71.06	81.80	**94.61**	100	83.95	67.12
2003	*52.04*	62.05	64.40	73.31	89.61	**97.42**	100	89.71	80.14
2004	*52.64*	72.09	68.43	71.70	89.02	**98.08**	100	92.42	65.50
2005	*42.74*	50.87	55.33	60.68	78.76	**91.68**	100	83.77	71.52
2006	*32.84*	36.45	43.42	45.80	75.59	**93.12**	100	79.23	62.80
2007	*52.71*	60.49	63.86	71.26	84.44	**95.68**	100	86.90	71.44
2008	*38.59*	52.40	47.82	60.54	85.59	**97.35**	100	84.67	64.09
2009	*33.54*	42.49	40.24	55.22	83.16	**95.05**	100	85.13	63.90
2010	*36.80*	44.01	52.63	67.29	79.25	**94.19**	100	84.67	68.84
2011	*44.55*	60.20	58.37	65.38	85.16	**94.53**	100	89.89	75.15
2012	*36.52*	45.69	50.29	62.36	73.23	**89.82**	100	87.53	65.97
2013	*39.34*	43.75	46.78	52.32	82.01	**95.53**	100	87.92	70.85
2014	*41.30*	54.72	53.61	60.35	81.28	**93.68**	100	85.93	69.66
2015	*51.59*	58.50	56.13	68.39	85.43	**94.95**	100	86.81	73.79
**Mean**	*42.20*	52.63	54.73	63.46	82.31	**94.58**	100	86.18	69.57
**sd**	6.96	9.24	8.05	7.86	4.53	2.19	0	3.16	4.81
**Median**	*39.56*	52.74	55.33	65.38	82.01	**94.61**	100	85.93	69.66
**MAD**	3.18	7.46	5.38	5.03	3.15	1.07	0	1.79	3.69

Exercise intensity levels based on the guidelines in Pelliccia et al. (2021, table 4):

**low intensity<br>HR% < 55**	**moderate intensity<br>55 ≤ HR% < 75**	**high intensity<br>75 ≤ HR% ≤ 90**	**very high intensity<br>HR% > 90**

Note: Individual percentages of maximal heart rate at each epoch are given by HR.*t* % = (HR.*t* / HR_{max}) 100%, where HR.*t* is the heart rate at epoch *t* (*t* = 1,…, 9) and HR_{max} is the individual maximal heart rate recorded during the bicycle stress test. For each athlete, 100% corresponds to HR_{max}; the minimum HR.*t* % reached over the epochs is written in italics; the highest HR.*t* % smaller than 100% is written in bold.

Supplementary Table 7. Quantile regression models of the three quartiles of each ANS-BS indicator against sex and age: *P*-values of the significance tests for null effects of sex, age, and their interaction.

	**1st Quartile**			**Median**			**3rd Quartile**		
**ANS-BS-Ind**	*sex*	*age*	*sex-by-age*	*sex*	*age*	*sex-by-age*	*sex*	*age*	*sex-by-age*
AMP-BS-Ind	0.479	0.051	0.550	0.297	0.107	0.332	0.473	0.175	0.449
FRE-BS-Ind	0.333	0.564	0.316	0.260	0.716	0.339	0.484	0.652	0.523

Note. The significance tests are based on the procedure described in Koenker (2005) with standard errors estimated by bootstrap with 1000 repetitions. Sex is included in the quantile regression models as a dummy variable, with 0 = female and 1 = male. No significant results are found.

Supplementary Table 8. Quantile regression models of the three quartiles of each ANS-WS indicator against sex and age: *P*-values of the significance tests for null effects of sex, age, and their interaction.

	**Amplitude WS indicator**								
	**1st Quartile**			**Median**			**3rd Quartile**		
**Epochs**	*sex*	*age*	*sex-by-age*	*sex*	*age*	*sex-by-age*	*sex*	*age*	*sex-by-age*
Epoch1	0.989	0.376	0.997	0.624	0.120	0.716	0.886	0.774	0.997
Epoch2	0.309	0.168	0.285	0.120	0.076	0.141	0.478	0.546	0.478
Epoch3	0.517	0.589	0.754	0.979	0.920	0.950	0.450	0.818	0.490
Epoch4	0.936	0.467	0.855	0.534	0.191	0.548	0.280	0.291	0.455
Epoch5	0.817	0.252	0.781	0.952	0.380	0.834	0.852	0.921	0.753
Epoch6	0.973	0.486	0.889	0.400	0.182	0.425	0.283	0.215	0.464
Epoch7	0.826	0.332	0.726	0.635	0.059	0.617	0.627	0.096	0.772
Epoch8	0.921	0.581	0.881	0.495	0.565	0.473	0.777	0.990	0.929
Epoch9	0.473	0.949	0.434	0.494	0.565	0.512	0.493	0.989	0.532
	**Signal Self-Similarity WS indicator**								
	**1st Quartile**			**Median**			**3rd Quartile**		
**Epochs**	*sex*	*age*	*sex-by-age*	*sex*	*age*	*sex-by-age*	*sex*	*age*	*sex-by-age*
Epoch1	0.935	0.922	0.912	0.732	0.927	0.729	0.544	0.363	0.531
Epoch2	0.494	0.837	0.625	0.512	0.597	0.686	0.144	0.214	0.139
Epoch3	0.885	0.551	0.834	0.954	0.803	0.897	0.945	0.752	0.784
Epoch4	0.913	0.839	0.791	0.46	0.661	0.548	0.490	0.934	0.590
Epoch5	0.898	0.718	0.995	0.352	0.936	0.402	0.768	0.965	0.911
Epoch6	0.889	0.247	0.900	0.238	0.185	0.253	0.084	0.168	0.106
Epoch7	0.531	0.695	0.506	0.600	0.872	0.558	0.993	0.463	0.933
Epoch8	0.545	0.616	0.619	0.549	0.625	0.686	0.556	0.994	0.629
Epoch9	0.964	0.795	0.983	0.854	0.828	0.932	0.550	0.817	0.725
	**Oscillatory WS indicator**								
	**1st Quartile**			**Median**			**3rd Quartile**		
**Epochs**	*sex*	*age*	*sex-by-age*	*sex*	*age*	*sex-by-age*	*sex*	*age*	*sex-by-age*
Epoch1	0.493	0.408	0.603	0.936	0.709	0.789	0.981	0.441	0.685
Epoch2	0.208	0.464	0.296	0.273	0.375	0.376	0.992	0.936	0.999
Epoch3	0.945	0.593	0.936	0.692	0.582	0.757	0.395	0.820	0.516
Epoch4	0.976	0.976	0.927	0.989	0.840	0.800	0.786	0.665	0.773
Epoch5	0.377	0.307	0.422	0.088	0.081	0.129	0.765	0.444	0.792
Epoch6	0.693	0.641	0.983	0.837	0.642	0.835	0.392	0.624	0.447
Epoch7	0.922	0.383	0.876	0.727	0.399	0.789	0.545	0.776	0.564
Epoch8	0.569	0.328	0.615	0.903	0.331	0.885	0.560	0.717	0.404
Epoch9	0.431	0.963	0.608	0.445	0.672	0.521	0.635	0.931	0.753

Note. The significance tests are based on the procedure described in Koenker (2005) with standard errors estimated by bootstrap with 1000 repetitions. Sex is included in the quantile regression models as a dummy variable, with 0 = female and 1 = male. No significant results are found.

Supplementary Table 9. Bootstrap confidence intervals at 95% confidence level for the total median scores of the three ANS-WS indicators at each epoch (the table refers to the first column of panels in Figure 6).

**Epochs**	**Amplitude WS indicator**			**Mt**	**Signal self-similarity WS indicator**			**Mt**	**Oscillatory WS indicator**			**Mt**
	Median	95%-Low CL	95%-Upper CL		Median	95%-Low CL	95%-Upper CL		Median	95%-Low CL	95%-Upper CL	
Epoch1	84.60	81.34	87.56	1,2	17.28	10.35	19.82	1	15.17	10.86	20.96	1
Epoch2	85.03	79.16	87.90	1,2	41.29	38.69	46.71	2,3,9	75.94	66.02	87.77	2,3,4
Epoch3	72.07	66.95	78.02	3,8	41.22	36.46	48.48	2,3,9	76.44	66.91	82.82	2,3,4
Epoch4	49.23	43.24	55.77	4,8,9	53.60	50.75	58.02	4,9	69.61	60.31	81.76	2,3,4
Epoch5	33.90	30.33	37.83	5,6	80.44	77.39	84.37	5,6,8	32.45	28.25	34.64	5,6,7
Epoch6	20.47	15.66	37.71	5,6	72.82	68.84	78.12	5,6	23.28	19.08	37.19	5,6,7
Epoch7	7.91	6.25	12.58	7	5.82	5.21	8.32	7	41.60	33.05	50.84	5,6,7,8,9
Epoch8	48.63	42.85	67.80	3,4,8,9	87.21	83.42	91.45	5,8	50.23	45.01	59.93	7,8,9
Epoch9	56.96	51.93	63.05	4,8,9	47.22	44.88	51.01	2,3,4,9	59.61	50.07	67.34	7,8,9

Legend. CL = Confidence Limit.

For each ANS-WS indicator, a green cell denotes the largest computed median (along with its C.I.) over the nine epochs, while a gray cell denotes the smallest computed median (with its C.I.) over the nine epochs (CL stands for confidence limit).

Columns labeled "Mt" contain the results of the non-parametric bootstrap median test carried out for each ANS-WS indicator and all the distinct pairwise comparisons between the median scores of two different epochs. Such pairwise comparisons are $\frac{9\times 8}{2} = 36$ in all for each ANS-WS indicator. Where the bootstrap C.I.s concerning the median scores of two epochs overlap, such medians are not significantly different in the athlete population. Conversely, where two bootstrap C.I.s do not overlap, the medians significantly differ at 0.05. Accordingly, this procedure implicitly gives a significance test for the null hypothesis of the equality of two medians at the 0.05 nominal significance level. Formally, let θ_t be the effect of epoch t on the population median $Med_t(Y)$ of the generic ANS-WS indicator Y, with $t = 1, \dots, 9$. By the Mt procedure, the null hypothesis $H_0: Med_t(Y) = Med_v(Y)$ is tested at the 0.05 significance level against the alternative hypothesis $H_1: Med_t(Y) \neq Med_v(Y)$, for each $t \neq v$. Numbers 1 to 9 in the three columns "Mt" express the test results obtained in the 36 distinct pairwise comparisons for every ANS-WS indicator. Precisely, within each "Mt" column, where the same number appears in a comparison between two epochs, then the corresponding two C.I.s overlap, and H_0 is accepted. Vice versa, where a number appears only once in a comparison between two epochs, then their corresponding C.I.s do not overlap, and H_0 is rejected at the 0.05 level in favor of H_1.

Supplementary Table 10. Bootstrap confidence intervals at 95% confidence level for the within-group median scores of the three ANS-WS indicators at each epoch (the table refers to the second column of panels in Figure 6).

Epochs	Groups	Amplitude WS indicator			Signal self-similarity WS indicator			Oscillatory WS indicator		
		Median	95%-Low CL	95%-Upper CL	Median	95%-Low CL	95%-Upper CL	Median	95%-Low CL	95%-Upper CL
Epoch1	Cycl	88.58	85.56	92.38	17.15	9.58	22.52	5.85	3.25	10.85
	Shoot	82.12	79.93	85.31	19.32	15.86	23.48	21.81	16.02	25.86
Epoch2	Cycl	88.55	81.00	94.03	34.03	26.90	44.13	75.20	60.18	89.07
	Shoot	84.39	80.32	86.95	43.84	42.68	46.40	78.49	70.48	89.27
Epoch3	Cycl	75.14	65.47	80.87	41.48	35.05	46.01	89.76	81.67	95.27
	Shoot	71.68	68.45	80.42	40.96	34.74	54.61	69.24	58.88	84.84
Epoch4	Cycl	41.49	32.38	50.82	64.38	61.33	70.78	88.32	82.35	92.52
	Shoot	52.69	47.05	60.04	51.38	47.14	55.15	60.16	50.34	69.65
Epoch5	Cycl	20.18	15.32	24.79	76.87	70.78	83.49	39.34	28.93	53.01
	Shoot	45.60	41.42	66.59	81.18	69.63	83.67	32.05	28.44	42.21
Epoch6	Cycl	12.62	9.11	19.44	72.67	66.00	80.70	19.15	13.25	29.02
	Shoot	34.50	28.78	61.46	72.96	67.35	78.54	35.82	27.75	51.53
Epoch7	Cycl	3.87	3.21	4.23	4.06	3.24	5.09	39.99	31.17	52.31
	Shoot	15.84	12.06	23.14	6.94	5.31	11.41	42.20	33.34	54.04
Epoch8	Cycl	48.82	37.44	58.27	84.56	74.71	92.29	73.67	62.50	89.21
	Shoot	48.45	43.31	71.20	88.39	87.45	89.97	41.34	33.75	51.38
Epoch9	Cycl	69.84	60.69	76.99	43.09	36.58	49.68	68.22	60.53	83.50
	Shoot	48.35	42.63	71.14	49.52	46.35	62.62	55.29	48.02	71.83

Legend. CL = Confidence Limit.

For each epoch and ANS-WS indicator, bootstrap C.I.s that are colored in light blue (Amplitude), yellow (Signal Self-Similarity), and green (Oscillatory), respectively, denote non-overlapping intervals in the comparison between the cyclist and shooter groups. Then, by the same remarks below in Supplementary Table 9, non-overlapping intervals indicate within-group median scores significantly different at the 0.05 level.

Supplementary Table 11. P-values of the ATS-based test for the pairwise comparisons between every two consecutive epochs in the whole athlete set (the table refers to the first column of panels in Figure 6).

	**AMP-WS-Ind**				**SSS-WS-Ind**				**OSC-WS-Ind**			
	**p-value**		**FDR adj.**		**p-value**		**FDR adj.**		**p-value**		**FDR adj.**	
**Epochs**	incr.	decr.	incr.	decr.	incr.	decr.	incr.	decr.	incr.	decr.	incr.	decr.
Ep1.2	0.423	0.577	1.000	0.769	<0.001	1.000	**<0.001**	1.000	<0.001	1.000	**<0.001**	1.000
Ep2.3	1.000	<0.001	1.000	**<0.001**	0.265	0.735	0.424	1.000	0.039	0.961	0.104	1.000
Ep3.4	1.000	<0.001	1.000	**<0.001**	0.068	0.932	0.136	1.000	0.989	0.011	1.000	**0.043**
Ep4.5	1.000	<0.001	1.000	**<0.001**	<0.001	1.000	**<0.001**	1.000	1.000	<0.001	1.000	**<0.001**
Ep5.6	1.000	<0.001	1.000	**<0.001**	0.992	0.008	1.000	**0.021**	0.972	0.028	1.000	0.076
Ep6.7	1.000	<0.001	1.000	**<0.001**	1.000	<0.001	1.000	**<0.001**	<0.001	1.000	**0.001**	1.000
Ep7.8	<0.001	1.000	**<0.001**	1.000	<0.001	1.000	**<0.001**	1.000	0.183	0.817	0.293	1.000
Ep8.9	0.006	0.994	**0.022**	1.000	1.000	<0.001	1.000	**<0.001**	0.141	0.859	0.282	1.000

Note. For each ANS-WS indicator Y, by the ATS-based test, the null hypothesis H_{0t}: $F_t(y) = F_{t+1}(y)$, for all y, is tested against the two alternatives: (a) H_{1t}: $F_t(y) > F_{t+1}(y)$ (increasing – incr.), and: (b) H_{1t}: $F_t(y) < F_{t+1}(y)$ (decreasing – decr.), for at least one y, for all $t = 1, \ldots, 8$, where $F_t(y)$ is the population cumulative distribution of Y at epoch t in the whole athlete set. The FDR p-value adjustment (columns labeled "FDR adj.") is then applied to preserve the nominal significance level associated with the overall "no epoch effect" null hypothesis: H_0^E: $F_1(y) = \ldots = F_t(y) = \ldots = F_9(y)$, for all y.

Supplementary Table 12. P-values of the WSR test for the pairwise comparisons between every two consecutive epochs in the whole athlete set (the table refers to the first column of panels in Figure 6).

	**AMP-WS-Ind**				**SSS-WS-Ind**				**OSC-WS-Ind**			
	**p-value**		**FDR adj.**		**p-value**		**FDR adj.**		**p-value**		**FDR adj.**	
**Epochs**	incr.	decr.	incr.	decr.	incr.	decr.	incr.	decr.	incr.	decr.	incr.	decr.
Ep1.2	0.184	0.808	0.490	1.000	<0.001	1.000	**<0.001**	1.000	<0.001	1.000	**0.002**	1.000
Ep2.3	1.000	<0.001	1.000	**<0.001**	0.318	0.666	0.509	1.000	0.065	0.936	0.174	1.000
Ep3.4	1.000	<0.001	1.000	**<0.001**	0.119	0.886	0.238	1.000	0.984	0.016	1.000	0.064
Ep4.5	1.000	<0.001	1.000	**<0.001**	<0.001	1.000	**<0.001**	1.000	1.000	<0.001	1.000	**0.003**
Ep5.6	1.000	<0.001	1.000	**<0.001**	0.983	0.017	1.000	**0.045**	0.943	0.050	1.000	0.134
Ep6.7	1.000	<0.001	1.000	**<0.001**	1.000	<0.001	1.000	**<0.001**	0.001	0.999	**0.004**	1.000
Ep7.8	<0.001	1.000	**0.002**	1.000	<0.001	1.000	**<0.001**	1.000	0.138	0.856	0.220	1.000
Ep8.9	0.002	0.999	**0.010**	1.000	1.000	<0.001	1.000	**<0.001**	0.128	0.868	0.220	1.000

Note. For each ANS-WS indicator Y, by the Wilcoxon Signed-Rank (WSR) test, the null hypothesis H_{0t}: $\theta_{t,t+1} = 0$ is tested against the two alternatives: (a) H_{1t}: $\theta_{t,t+1} < 0$ (increasing – incr.), and: H_{1t}: $\theta_{t,t+1} > 0$ (decreasing – decr.), for all $t = 1, \ldots, 8$, where $\theta_{t,t+1}$ is the median of the difference $Y_t - Y_{t+1}$ of Y between two consecutive epochs. The FDR p-value adjustment (columns labeled "FDR adj.") is then applied to preserve the nominal significance level associated with the overall "no epoch effect" null hypothesis: H_0^E: $\theta_{1,2} = \ldots = \theta_{t,t+1} = \ldots = \theta_{8,9} = 0$.

Supplementary Table 13. P-values of the BA permutation test for comparing cyclists and shooters at each epoch based on the three ANS-WS indicators (the table refers to the second column of panels in Figure 6).

	**AMP-WS-Ind**		**SSS-WS-Ind**		**OSC-WS-Ind**	
**Epochs**	p-value	FDR adj.	p-value	FDR adj.	p-value	FDR adj.
Epoch1	0.225	0.328	0.600	0.784	0.015	**0.034**
Epoch2	0.255	0.328	0.259	0.599	0.687	0.879
Epoch3	0.746	0.746	0.610	0.784	0.013	**0.034**
Epoch4	0.072	0.130	0.247	0.599	0.010	**0.034**
Epoch5	<0.001	**<0.001**	0.596	0.784	0.781	0.879
Epoch6	<0.001	**<0.001**	0.811	0.845	0.003	**0.027**
Epoch7	<0.001	**<0.001**	0.150	0.599	0.894	0.894
Epoch8	0.428	0.482	0.845	0.845	0.021	**0.038**
Epoch9	0.070	0.130	0.266	0.599	0.054	0.081

Note. For each ANS-WS indicator Y, by the Bowman-Azzalini (BA) test, the null hypothesis $H_{0t}: f_{tC}(y) = f_{tS}(y)$, for all y, is tested with fixed epoch t against the alternative hypothesis $H_{1t}: f_{tC}(y) \neq f_{tS}(y)$, for at least one y, where $f_{tC}(y)$ and $f_{tS}(y)$ are the population density functions within the cyclist (C) and shooter (S) groups at the epoch t, with $t = 1, \dots, 9$. The FDR p-value adjustment (columns labeled "FDR adj.") is then applied to preserve the nominal significance level associated with the overall "no group effect" null hypothesis: $H_0^G: f_{tC}(y) = f_{tS}(y)$ for all t and y. The relevant FDR-adjusted p-values concerning the three ANS-WS indicators are reported in the panels displayed in Supplementary Figures 4-6.

Supplementary Table 14. P-values of the JT permutation test for comparing cyclists and shooters at each epoch based on the three ANS-WS indicators (the table refers to the second column of panels in Figure 6).

	**Amplitude WS indicator**					
	**p-value**			**FDR-adjusted p-value**		
**Epochs**	**2-sided**	**C ↓ S**	**C ↑ S**	**2-sided**	**C ↓ S**	**C ↑ S**
Epoch1	0.089	0.959	0.041	0.161	0.995	0.179
Epoch2	0.118	0.947	0.060	0.165	0.995	0.179
Epoch3	0.902	0.459	0.551	0.902	0.826	0.993
Epoch4	0.128	0.066	0.935	0.165	0.149	1.000
Epoch5	<0.001	<0.001	1.000	**0.002**	**<0.001**	1.000
Epoch6	<0.001	<0.001	1.000	**0.002**	**<0.001**	1.000
Epoch7	<0.001	<0.001	1.000	**0.002**	**0.001**	1.000
Epoch8	0.848	0.597	0.403	0.902	0.896	0.906
Epoch9	0.008	0.995	0.004	**0.019**	0.995	**0.038**
	**Signal Self-Similarity WS indicator**					
	**p-value**			**FDR-adjusted p-value**		
**Epochs**	**2-sided**	**C ↓ S**	**C ↑ S**	**2-sided**	**C ↓ S**	**C ↑ S**
Epoch1	0.587	0.286	0.732	0.959	0.758	0.942
Epoch2	0.926	0.470	0.553	0.959	0.791	0.942
Epoch3	0.412	0.808	0.199	0.926	0.883	0.896
Epoch4	0.251	0.883	0.119	0.754	0.883	0.896
Epoch5	0.708	0.337	0.659	0.959	0.758	0.942
Epoch6	0.793	0.631	0.389	0.959	0.812	0.942
Epoch7	0.032	0.015	0.988	0.292	0.139	0.988
Epoch8	0.959	0.527	0.487	0.959	0.791	0.942
Epoch9	0.184	0.095	0.918	0.754	0.428	0.988
	**Oscillatory WS indicator**					
	**p-value**			**FDR-adjusted p-value**		
**Epochs**	**2-sided**	**C ↓ S**	**C ↑ S**	**2-sided**	**C ↓ S**	**C ↑ S**
Epoch1	0.005	0.002	0.999	**0.023**	**0.014**	0.999
Epoch2	0.482	0.250	0.776	0.563	0.601	0.998
Epoch3	0.005	0.997	0.003	**0.023**	0.997	**0.025**
Epoch4	0.014	0.994	0.009	**0.041**	0.997	**0.030**
Epoch5	0.563	0.740	0.268	0.563	0.997	0.482
Epoch6	0.045	0.021	0.982	0.081	0.096	0.999
Epoch7	0.553	0.267	0.742	0.563	0.601	0.998
Epoch8	0.018	0.990	0.010	**0.041**	0.997	**0.030**
Epoch9	0.178	0.919	0.084	0.267	0.997	0.188

Note. For each ANS-WS indicator Y, by the Jonckheere-Terpstra (JT) permutation test, the null hypothesis $H_{0t}\colon \tau_{tC} = \tau_{tS}$ is tested with fixed epoch t against the two ordered alternatives: (a) $H_{1t}\colon \tau_{tC} < \tau_{tS}$ ($C \downarrow S$), and: (b) $H_{1t}\colon \tau_{tC} > \tau_{tS}$ ($C \uparrow S$), where τ_{tC} and τ_{tS} are the population effects of the C and S groups, respectively, on Y, at the epoch t, with $t = 1, \ldots, 9$. For completeness, results concerning the two-sided alternative $H_{1t}\colon \tau_{tC} \neq \tau_{tS}$ at the epoch t are also provided. The FDR p-value adjustment is then applied to preserve the nominal significance level associated with the overall "no group effect" null hypothesis: $H_0^G\colon \tau_{tC} = \tau_{tS}$ for all t. The relevant FDR-adjusted p-values concerning the three ANS-WS indicators are reported in the panels displayed in Supplementary Figures 4-6.

Supplementary Table 15. *P*-values of the KS bootstrap test for comparing cyclists and shooters at each epoch based on the three ANS-WS indicators (the table refers to the second column of panels in Figure 6).

	**Amplitude WS indicator**					
	***p*-value**			**FDR-adjusted *p*-value**		
**Epochs**	**2-sided**	**C↓S**	**C↑S**	**2-sided**	**C↓S**	**C↑S**
Epoch1	0.363	0.918	0.178	0.467	1.000	0.534
Epoch2	0.173	1.000	0.096	0.260	1.000	0.432
Epoch3	0.894	0.720	0.535	0.894	1.000	0.963
Epoch4	0.135	0.067	0.920	0.243	0.151	1.000
Epoch5	<0.001	<0.001	1.000	**<0.001**	**<0.001**	1.000
Epoch6	<0.001	<0.001	1.000	**<0.001**	**<0.001**	1.000
Epoch7	<0.001	<0.001	1.000	**<0.001**	**<0.001**	1.000
Epoch8	0.615	0.499	0.332	0.692	0.898	0.747
Epoch9	0.021	1.000	0.012	**0.047**	1.000	0.108
	**Signal Self-Similarity WS indicator**					
	***p*-value**			**FDR-adjusted *p*-value**		
**Epochs**	**2-sided**	**C↓S**	**C↑S**	**2-sided**	**C↓S**	**C↑S**
Epoch1	0.631	0.329	0.717	0.811	0.592	0.922
Epoch2	0.336	0.169	0.321	0.756	0.507	0.922
Epoch3	0.588	0.906	0.304	0.811	0.911	0.922
Epoch4	0.144	0.911	0.062	0.689	0.911	0.558
Epoch5	0.613	0.322	0.508	0.811	0.592	0.922
Epoch6	0.907	0.524	0.518	0.907	0.674	0.922
Epoch7	0.153	0.072	1.000	0.689	0.507	1.000
Epoch8	0.901	0.512	0.709	0.907	0.674	0.922
Epoch9	0.295	0.143	0.918	0.756	0.507	1.000
	**Oscillatory WS indicator**					
	***p*-value**			**FDR-adjusted *p*-value**		
**Epochs**	**2-sided**	**C↓S**	**C↑S**	**2-sided**	**C↓S**	**C↑S**
Epoch1	0.008	0.001	1.000	**0.040**	**0.009**	1.000
Epoch2	0.889	0.496	0.733	0.889	0.974	1.000
Epoch3	0.016	0.897	0.007	**0.043**	1.000	**0.036**
Epoch4	0.024	0.892	0.012	**0.043**	1.000	**0.036**
Epoch5	0.605	0.909	0.280	0.778	1.000	0.504
Epoch6	0.009	0.007	0.905	**0.040**	**0.032**	1.000
Epoch7	0.888	0.541	0.889	0.889	0.974	1.000
Epoch8	0.022	1.000	0.008	**0.043**	1.000	**0.036**
Epoch9	0.059	0.528	0.035	0.089	0.974	0.079

Note. For each ANS-WS indicator Y, by the Kolmogorov-Smirnov (KS) bootstrap test, the null hypothesis $H_{0t}\colon F_{tC}(y) = F_{tS}(y)$, for all y, is tested with fixed epoch t against the two alternative hypotheses: (a) $H_{1t}\colon F_{tC}(y) > F_{tS}(y)$ ($C \downarrow S$), and (b) $H_{1t}\colon F_{tC}(y) < F_{tS}(y)$ ($C \uparrow S$), for at least one y, where $F_{tC}(y)$ and $F_{tS}(y)$ are the population cumulative distribution functions within the cyclist (C) and shooter (S) groups at the epoch t, with $t = 1, \dots, 9$. The FDR p-value adjustment is then applied to preserve the nominal significance level associated with the overall "no group effect" null hypothesis: $H_0^G\colon F_{tC}(y) = F_{tS}(y)$ for all t and y. The relevant FDR-adjusted p-values concerning the three ANS-WS indicators are reported in the panels displayed in Supplementary Figures 4-6.

Supplementary Table 16. P-values of the Studentized Wilcoxon Rank-Sum (StWRS) permutation test for comparing cyclists and shooters at each epoch based on the three ANS-WS indicators (the table refers to the second column of panels in Figure 6).

	**Amplitude WS indicator**					
	***p*-value**			**FDR-adjusted *p*-value**		
**Epochs**	**2-sided**	**C↓S**	**C↑S**	**2-sided**	**C↓S**	**C↑S**
Epoch1	0.077	0.963	0.035	0.138	0.997	0.159
Epoch2	0.111	0.948	0.059	0.167	0.997	0.176
Epoch3	0.883	0.438	0.560	0.883	0.789	1.000
Epoch4	0.141	0.070	0.933	0.182	0.157	1.000
Epoch5	<0.001	<0.001	1.000	**<0.001**	**<0.001**	1.000
Epoch6	<0.001	<0.001	1.000	**<0.001**	**<0.001**	1.000
Epoch7	<0.001	<0.001	1.000	**<0.001**	**<0.001**	1.000
Epoch8	0.828	0.582	0.416	0.883	0.873	0.936
Epoch9	0.007	0.997	0.003	**0.015**	0.997	**0.023**
	**Signal Self-Similarity WS indicator**					
	***p*-value**			**FDR-adjusted *p*-value**		
**Epochs**	**2-sided**	**C↓S**	**C↑S**	**2-sided**	**C↓S**	**C↑S**
Epoch1	0.547	0.285	0.728	0.945	0.748	0.935
Epoch2	0.931	0.470	0.535	0.945	0.788	0.935
Epoch3	0.391	0.811	0.204	0.880	0.876	0.920
Epoch4	0.245	0.876	0.130	0.735	0.876	0.920
Epoch5	0.677	0.333	0.665	0.945	0.748	0.935
Epoch6	0.756	0.622	0.377	0.945	0.799	0.935
Epoch7	0.025	0.011	0.990	0.229	0.099	0.990
Epoch8	0.945	0.525	0.485	0.945	0.788	0.935
Epoch9	0.173	0.081	0.910	0.735	0.364	0.990
	**Oscillatory WS indicator**					
	***p*-value**			**FDR-adjusted *p*-value**		
**Epochs**	**2-sided**	**C↓S**	**C↑S**	**2-sided**	**C↓S**	**C↑S**
Epoch1	0.004	0.002	0.998	**0.022**	**0.016**	0.998
Epoch2	0.476	0.246	0.756	0.524	0.598	0.971
Epoch3	0.005	0.998	0.003	**0.022**	0.998	**0.023**
Epoch4	0.016	0.993	0.011	**0.045**	0.998	**0.032**
Epoch5	0.524	0.742	0.267	0.524	0.998	0.481
Epoch6	0.050	0.027	0.974	0.091	0.122	0.998
Epoch7	0.523	0.266	0.741	0.524	0.598	0.971
Epoch8	0.020	0.991	0.008	**0.045**	0.998	**0.032**
Epoch9	0.198	0.902	0.100	0.297	0.998	0.225

Note. For each ANS-WS indicator Y, by the Studentized Wilcoxon Rank-Sum (StWRS) permutation test, the null hypothesis $H_{0t}: \theta_{t,C-S} = 0$ is tested with fixed epoch t against the two ordered alternatives: (a) $H_{1t}: \theta_{t,C-S} < 0$ ($C \downarrow S$), and: (b) $H_{1t}: \theta_{t,C-S} > 0$ ($C \uparrow S$), where $\theta_{t,C-S}$ is the median of the difference $Y_C - Y_S$ at the epoch t, with $t = 1, \ldots, 9$, between Y in cyclists (Y_C) and shooters (Y_S). For completeness, results concerning the two-sided alternative $H_{1t}: \theta_{t,C-S} \neq 0$ at the epoch t are also provided. The FDR p-value adjustment is then applied to preserve the nominal significance level associated with the overall "no group effect" null hypothesis: $H_0^G: \theta_{t,C-S} = 0$ for all t. The relevant FDR-adjusted p-values concerning the three ANS-WS indicators are reported in the panels displayed in Supplementary Figures 4-6.

Supplementary Table 17. Evaluation of the sensitivity level of the three ANS-WS indicators in detecting the rest-stand postural change (epochs 1-2) in the whole athlete set: Number of significant results concerning the 95% bootstrap C.I.s in Supplementary Table 9, the ATS-based test in Supplementary Table 11, and the WSR test in Supplementary Table 12 (row labeled "Ep1.2").

**ANS-WS indicators**	**95% bootstrap C.I.s**	**ATS-based test**	**WSR test**	**Average percentage of significant results**	**Sensitivity level**
AMP-WS-Ind	no	no	no	0%	**null**
SSS-WS-Ind	yes	yes	yes	100%	**strong**
OSC-WS-Ind	yes	yes	yes	100%	**strong**

Legend. no = not significant; yes = significant at the 0.05 level.

Sensitivity level of the three ANS-WS indicators to rest-stand postural change:

null: 0% ⊢⊣ 20%	weak: 20% ⊣ 40%	medium: 40% ⊣ 75%	strong: 75% ⊣ 100%

(based on the average percentage of significant results).

Supplementary Table 18. Evaluation of the sensitivity level of the three ANS-WS indicators in detecting the step changes in the exercise fraction (epochs 2-9) in the whole athlete set: Number of significant results concerning the 95% bootstrap C.I.s in Supplementary Table 9, the ATS-based test in Table 11, and the WSR test in Table 12 (rows labeled from "Ep2.3" to "Ep.8.9").

**ANS-WS indicators**	**95% bootstrap C.I.s**	**ATS-based test**	**WSR test**	**Average percentage of significant results**	**Sensitivity level**
AMP-WS-Ind	5 / 7	7 / 7	7 / 7	90.48%	**strong**
SSS-WS-Ind	5 / 7	5 / 7	5 / 7	71.43%	**medium**
OSC-WS-Ind	1 / 7	3 / 7	2 / 7	28.57%	**weak**

Legend. The evaluation is based on 7 pairwise comparisons between consecutive epochs from epoch 2 to epoch 9. Therefore, for each considered test, the cells contain the number of significant results out of the 7 total pairwise comparisons.

Sensitivity level of the three ANS-WS indicators to changes in the exercise fraction:

null: 0% ⊢⊣ 20%	weak: 20% ⊣ 40%	medium: 40% ⊣ 75%	strong: 75% ⊣ 100%

(based on the average percentage of significant results).

Supplementary Table 19. Evaluation of the sensitivity level of the three ANS-WS indicators in detecting sports specialties differences (cyclists vs. shooters): Number of significant results concerning the overall ATS-based tests for the group or group-by-interaction effects (second column, Figure 6), the 95% bootstrap C.I.s in Supplementary Table 10, the BA test in Supplementary Table 13, the JT test in Supplementary Table 14, the KS test in Supplementary Table 15, and the StWRS test in Supplementary Table 16.

**ANS-WS indicators**	**Overall ATS-based tests for group or group-by-epoch effects**	**95% bootstrap C.I.s(*)**	**BA test(*)**	**JT test(*)**	**KS test(*)**	**StWRS test(*)**	**Average percentage of significant results**	**Sensitivity level**
AMP-WS-Ind	2 / 2	4 / 9	3 / 9	4 / 9	4 / 9	4 / 9	44.68%	**medium**
SSS-WS-Ind	0 / 2	1 / 9	0 / 9	0 / 9	0 / 9	0 / 9	2.13%	**null**
OSC-WS-Ind	2 / 2	4 / 9	5 / 9	4 / 9	5 / 9	4 / 9	51.06%	**medium**

(*)*Legend.* The evaluation is based on comparing cyclists and shooters at each of the 9 epochs. Therefore, for each considered test, the cells contain the number of significant results out of the total number of 9 comparisons.

Sensitivity level of the three ANS-WS indicators to sports specialties differences:

null: 0% ⊢⊣ 20%	weak: 20% ⊣ 40%	medium: 40% ⊣ 75%	strong: 75% ⊣ 100%

(based on the average percentage of significant results).

2.2 Supplementary Figures

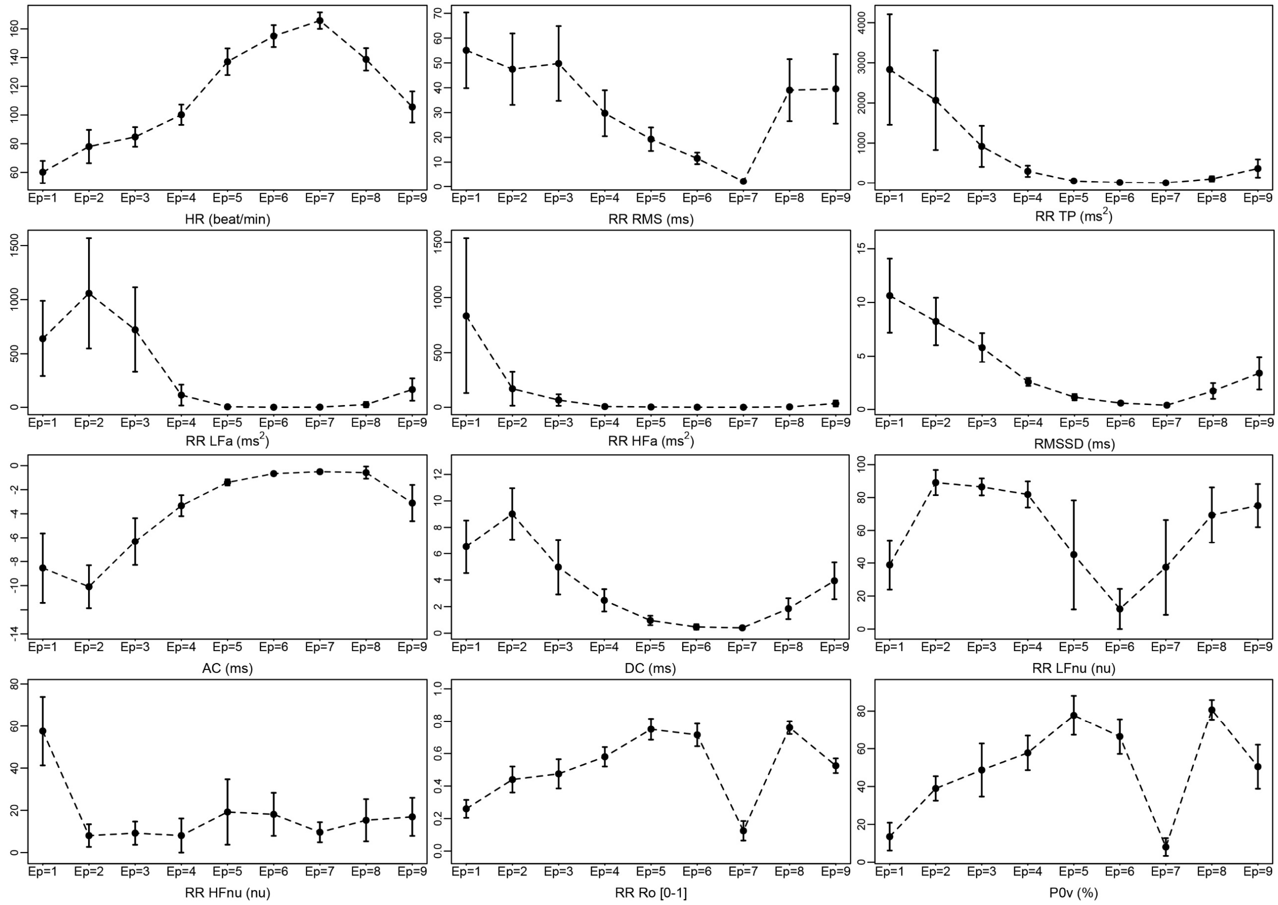

Supplementary Figure 1. Median profile plots of the study ANS proxies computed for all the athletes over the nine epochs.

Note: Error bars around the median are given by ± MAD (Median Absolute Deviation). Numerical data are reported in Supplementary Table 1, with the median and MAD computed over all the epochs, and in Supplementary Table 2, with the median and MAD computed at each epoch.

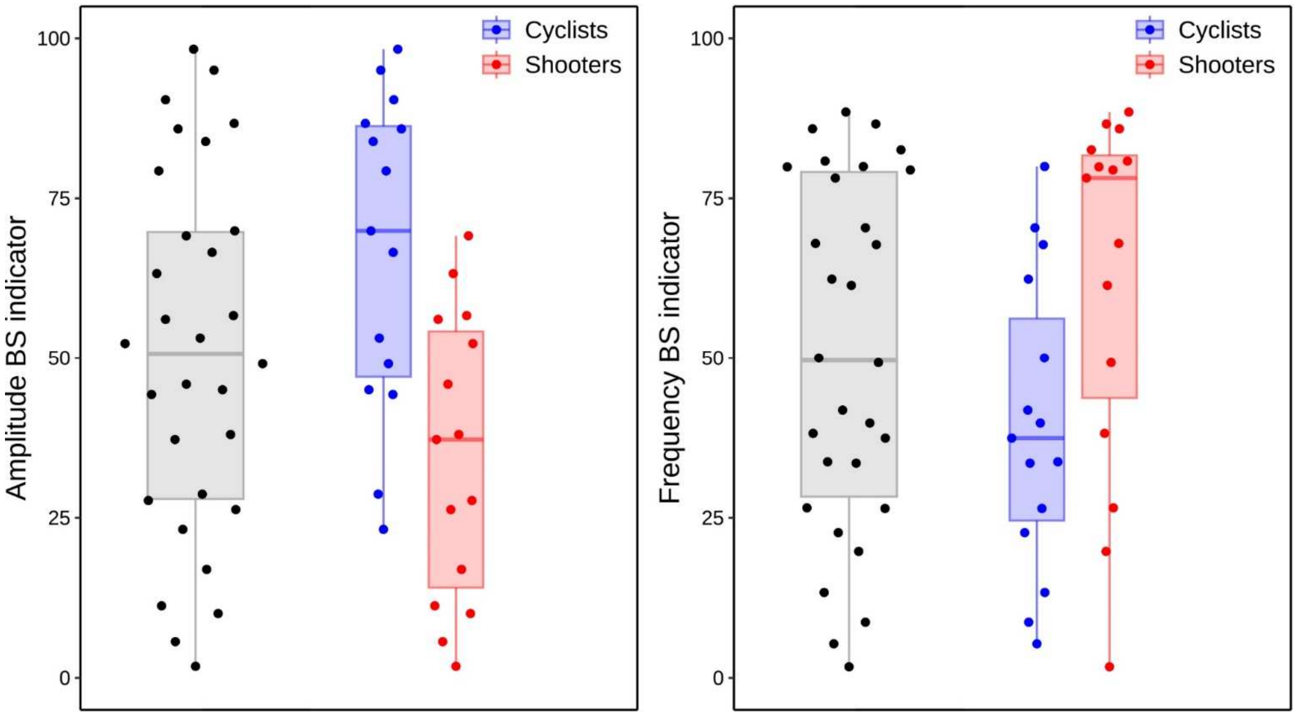

Supplementary Figure 2. Total and within-group beeswarm plots (along with box plots on the background) of the amplitude and frequency BS indicator distributions.

Note: Given the limited number of athletes, the beeswarm plot (i.e., a dot plot version with non-overlapping points) is an appropriate tool for displaying the ANS indicator distributions. Nevertheless, each beeswarm plot embodies a box plot in the background to provide a clearer picture of the main distribution characteristics. The black beeswarm plot in each panel refers to the whole athlete set.

Meaning of the ANS-BS indicator scores (based on the factor loadings in Table 3 in the text):

– Amplitude BS indicator (first panel): *Low amplitude levels* (i.e., lower scores close to 0) correspond to the athletes having the lowest RR RMS, RR TP, RR LFa, RR HFa, RMSSD, and DC average values and the highest AC average values on the entire exercise; *high amplitude levels* (i.e., higher scores close to 100) correspond to the athletes having the highest RR RMS, RR TP, RR LFa, RR HFa, RMSSD, and DC average values and the lowest AC average values on the entire exercise.
– Frequency BS indicator (second panel): *Low frequency levels* (i.e., lower scores close to 0) denote the athletes with the lowest RR LFnu, RR Ro, and P0v average values and the highest RR HFnu average values on the entire exercise; *high frequency levels* (i.e., higher scores close to 100) denote the athletes with the highest RR LFnu, RR Ro, and P0v average values and the lowest RR HFnu average values on the entire exercise.

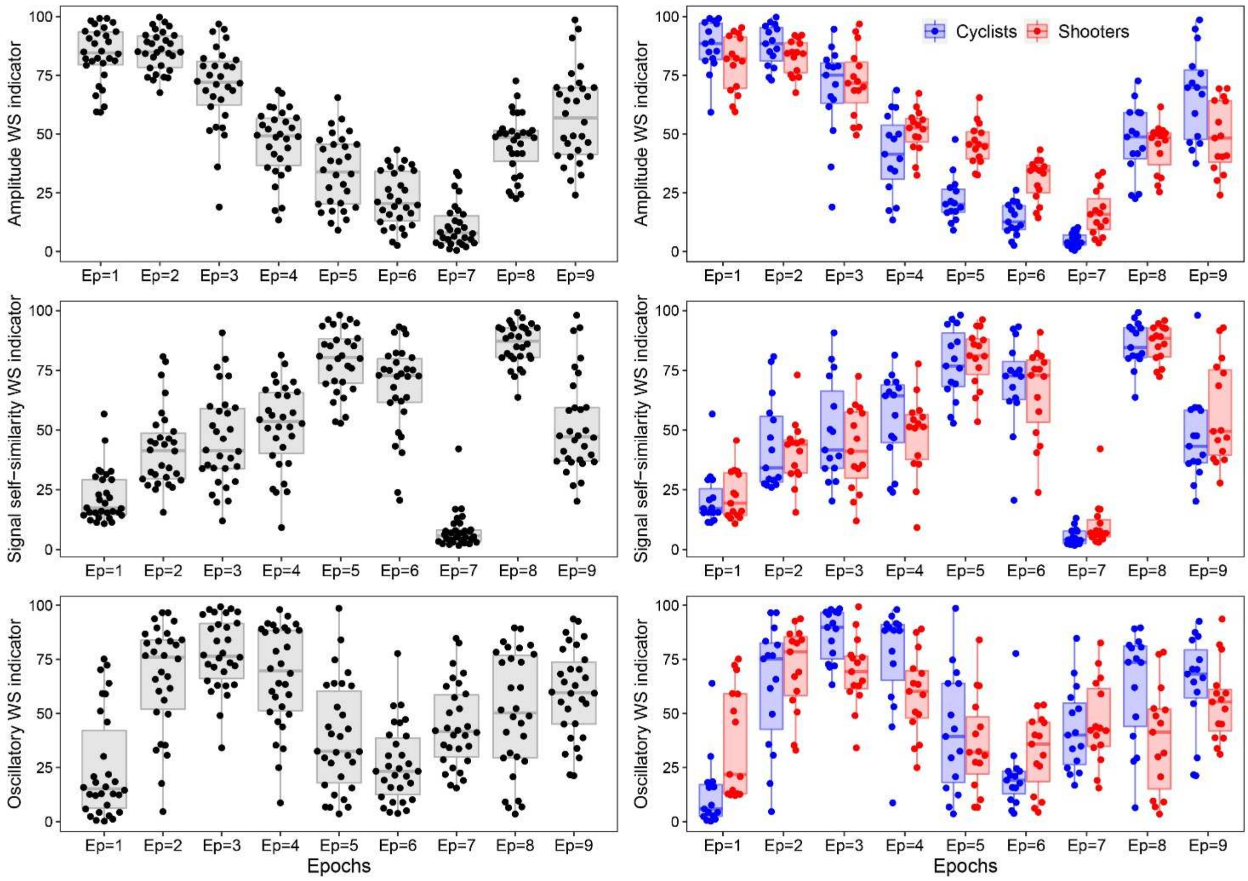

Supplementary Figure 3. Total (first column of panels) and within-group (second column) beeswarm plots (along with box plots on the background) of the distributions of the amplitude, signal self-similarity, and oscillatory WS indicators at each epoch.

Note: Each beeswarm plot embodies a box plot in the background to provide a clearer picture of the main ANS-WS distribution characteristics at each epoch.

Meaning of the ANS-WS indicator scores (based on the factor loadings in Table 4 in the text):

– Amplitude WS indicator: *Low amplitude levels* (i.e., lower scores close to 0) correspond to the athletes having the lowest RR RMS, RR TP, RR LFa, RR HFa, RMSSD, and DC (within-athletes centered) values and the highest AC and HR (within-athletes centered) values; *high amplitude levels* (i.e., higher scores close to 100) correspond to the athletes having the opposite characteristics;
– Signal self-similarity WS indicator: *Low signal self-similarity levels* (i.e., lower scores close to 0) denote the athletes with the lowest RR Ro and P0v (within-athletes centered) values; *high signal self-similarity levels* (i.e., higher scores close to 100) denote the athletes with the opposite characteristics;
– Oscillatory WS indicator: *Low oscillatory levels* (i.e., lower scores close to 0) denote the athletes with the lowest RR LFnu (within-athletes centered) values and the highest RR HFnu (within-athletes centered) values; *high oscillatory levels* (i.e., higher scores close to 100) denote the athletes with the opposite characteristics.

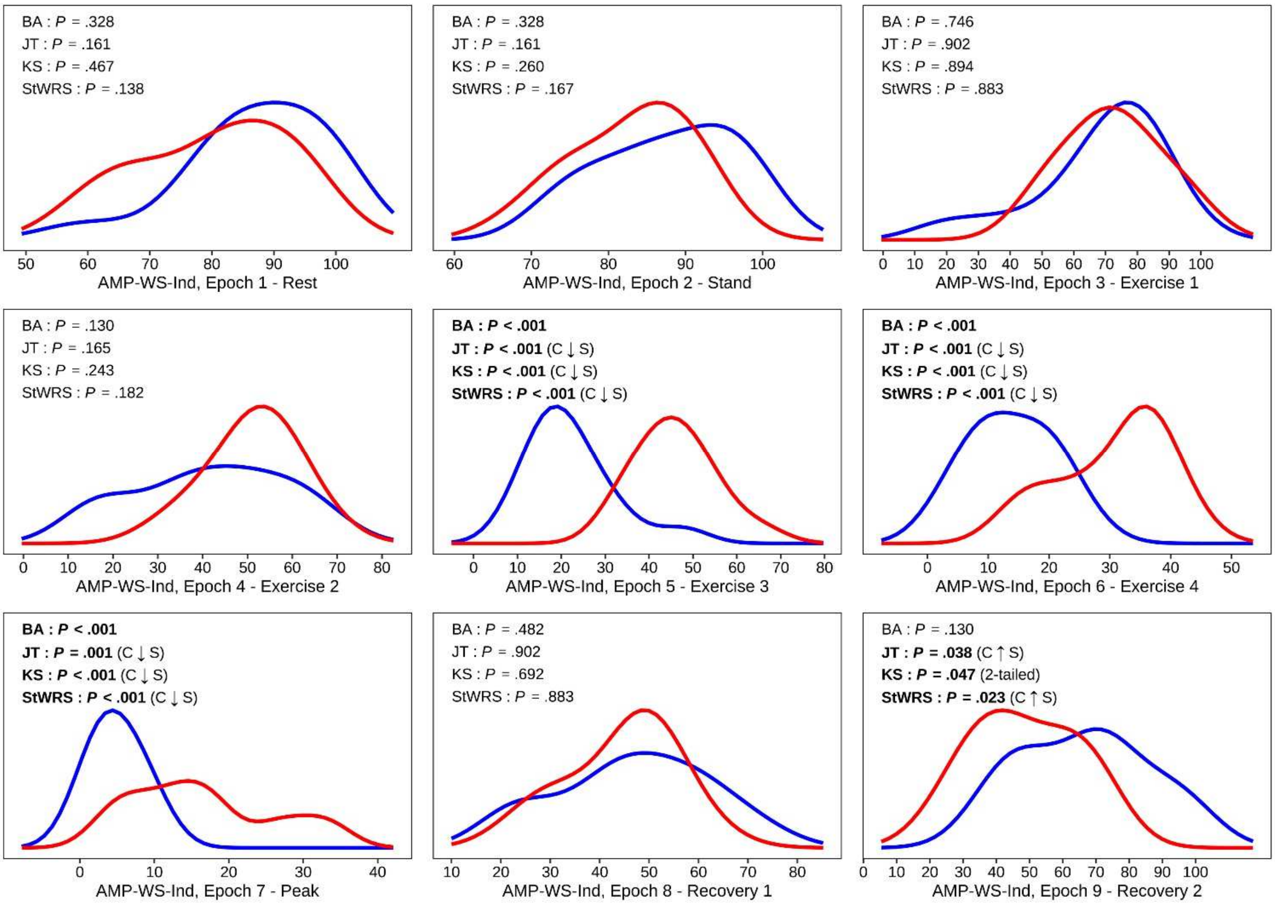

Supplementary Figure 4. Panel plot of the within-group estimated density curves with the BA method of the amplitude WS indicator distribution at each epoch.

Note: The meaning of the four statistical tests reported in each panel is given in Figure 1 in the text. Significant results at the 0.05 level are written in bold.

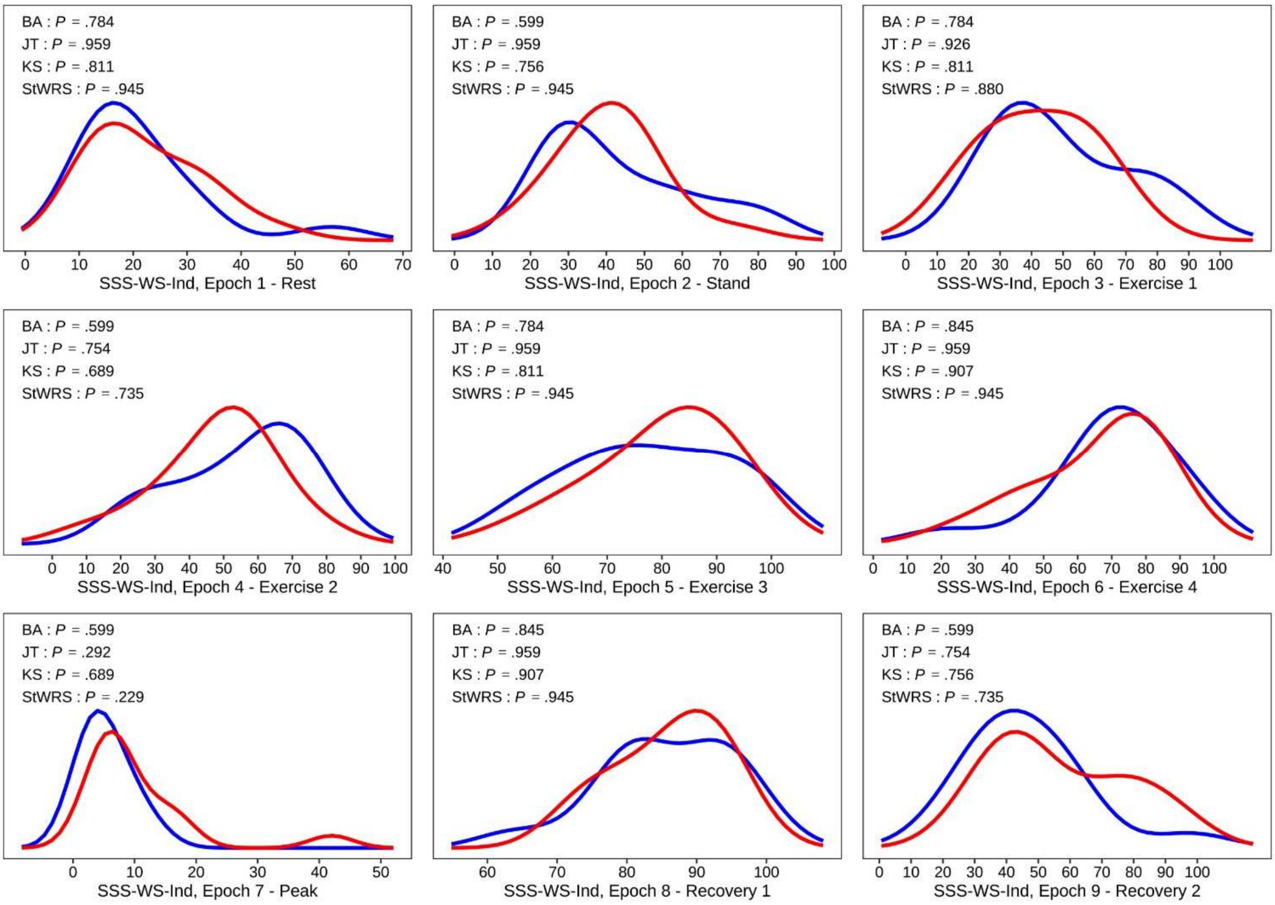

Supplementary Figure 5. Panel plot of the within-group estimated density curves with the BA method of the signal self-similarity WS indicator distribution at each epoch.

Note: The meaning of the four statistical tests reported in each panel is given in Figure 1 in the text. Significant results at the 0.05 level are written in bold.

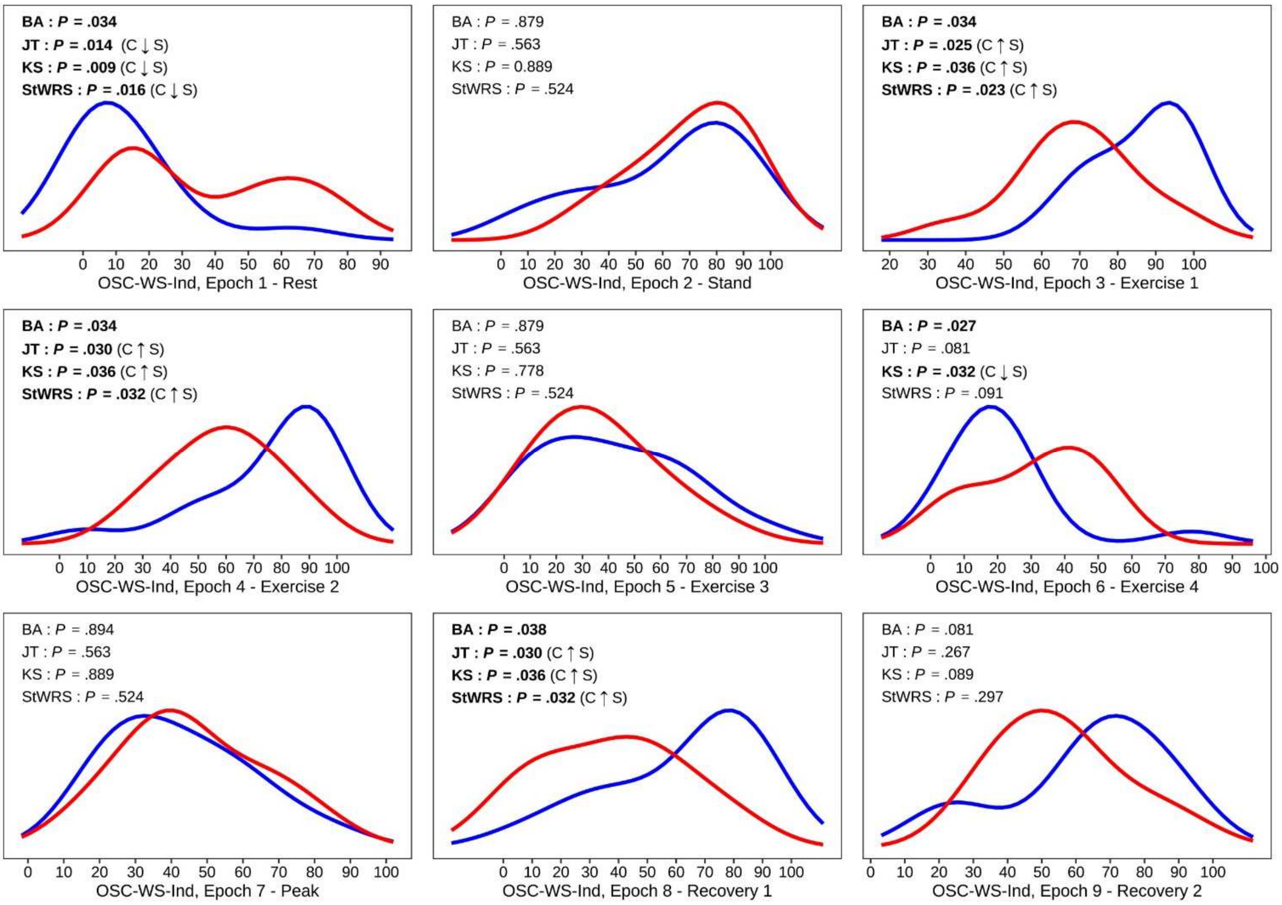

Supplementary Figure 6. Panel plot of the within-group estimated density curves with the BA method of the oscillatory WS indicator distribution at each epoch.

Note: The meaning of the four statistical tests reported in each panel is given in Figure 1 in the text. Significant results at the 0.05 level are written in bold.
